# Shifting burden of nasopharyngeal carcinoma: global patterns and forecasts to 2050 from the GBD 2021

**DOI:** 10.3389/fonc.2025.1687320

**Published:** 2026-01-06

**Authors:** Enhui Zhou, Feifei Xu, Tianjiao Zhou, Jingyu Zhang, Fan Song, Jianxiang Li, Hongliang Yi, Qingliang Wang, Weijun Huang

**Affiliations:** 1 Department of Otorhinolaryngology Head and Neck Surgery, Shanghai Sixth People’s Hospital Affiliated to Shanghai Jiao Tong University School of Medicine, Shanghai, China; 2 Shanghai Key Laboratory of Sleep Disordered Breathing, Shanghai, China; 3 Otolaryngology Institute of Shanghai Jiao Tong University, Shanghai, China; 4 Department of Otorhinolaryngology Head and Neck Surgery, Shanghai General Hospital Affiliated to Shanghai Jiao Tong University School of Medicine, Shanghai, China; 5 Department of Otolaryngology, Shanghai Sixth People’s Hospital Fujian, Jinjiang, China; 6 Department of Hepatobiliary-Pancreatic-Splenic Surgery, The Third Affiliated Hospital, Sun Yat-sen University, Guangzhou, China

**Keywords:** Bayesian age-period-cohort model, global burden of disease, incidence, mortality, nasopharyngeal carcinoma

## Abstract

**Background:**

Nasopharyngeal carcinoma (NPC) exhibits pronounced geographical variation, with a high burden in specific regions. We assessed global, regional, and national trends in NPC burden from 1990 to 2021 and projected estimates to 2050.

**Methods:**

We analyzed data from the Global Burden of Disease (GBD) 2021 study for 204 countries and territories across 21 regions. Age-standardized incidence rate (ASIR), age-standardized death rate (ASDR), and age-standardized disability-adjusted life-years (DALYs) rate were estimated, and temporal trends were assessed using estimated annual percentage change (EAPC) by socio-demographic index (SDI), sex, and age group. Future burden from 2022 to 2050 was projected using a Bayesian age–period–cohort model.

**Results:**

In 2021, there were 118,878 incident cases, 75,359 deaths, and 2,490,191 DALYs due to NPC globally. The global ASIR was 1.38 per 100,000 population (EAPC −1.06), the ASDR was 0.87 (EAPC −2.17), and the age-standardized DALYs rate was 28.91 (EAPC −2.29). Incidence rate peaked at ages 65–69 years (4.23 per 100,000 population), with consistently higher rates in males than in females. East Asia had the highest regional ASIR, and Japan bore the highest age-specific disease burden. Projections indicate continued declines in apparent ASDR and DALYs rates globally to 2050, but rising ASIR in males, particularly in East Asia and high-middle SDI regions.

**Conclusions:**

Over the past three decades, global mortality and disability burden from NPC have decreased, whereas incidence has increased in selected populations. The projected rise in ASIR among males in high-risk regions highlights the need for targeted interventions, equitable resource allocation, and sustained surveillance to mitigate future burden.

## Background

Nasopharyngeal carcinoma (NPC) is an epithelial cancer of the head and neck that originates in the mucosal lining of the nasopharynx. In 2020, NPC caused approximately, 133,000 cases and 80,000 deaths globally, with over 75% occurring in Southeast Asia and southern China (1), and men affected three times more often than women (2). In addition to its clear association with Epstein–Barr virus (EBV) infection, environmental factors and genetic susceptibility are thought to contribute to a higher risk of developing NPC (3), including well-documented dietary exposures such as salted fish in endemic regions (4). According to the World Health Organization (WHO) pathological classification, NPC is divided into keratinizing squamous cell carcinoma and non-keratinizing carcinoma. While early-stage NPC can be effectively treated, the response to treatment for advanced NPC remains unsatisfactory, with an overall five-year survival rate of 38%−63%, largely due to the high rate of early misdiagnosis (over 70% are initially diagnosed at stage III or IV) (5). Therefore, a thorough understanding of cross-regional socio-demographic patterns of NPC can inform medical policies and enhance governmental awareness to promote early diagnosis and management. Previous studies on the global burden of NPC have been limited by sociological factors, such as population structure, sex disparities, and regional economic variations (6). Although several studies have examined national NPC burden and highlighted policy needs for high-risk regions and younger populations, the data in these studies were only updated to 2019 (7, 8). These constraints hinder a thorough understanding of the current NPC global impact.

The Global Burden of Disease Study (GBD) 2021 database provides a valuable resource for assessing the global burden of diseases and injuries (9), using standardized methodologies to ensure consistency and comparability across populations and temporal trends (10). More comprehensive global statistics may be necessary to further verify the true burden of NPC. Disparities in NPC incidence and mortality persist worldwide, highlighting the need for governments and policymakers to raise awareness about prevention and ensure judicious allocation of medical resources. This study used the latest GBD data from 1990 to 2021 to assess the global burden and temporal trends of NPC, offering insights for tailored approaches to alleviate the global impact of the disease.

## Methods

### Data source

Data relevant to the keywords were retrieved and downloaded from the GBD Results Tool on the Global Burden of Disease (GBD) database website (https://ghdx.healthdata.org/gbd-2021). We obtained data on the sex, age, incidence, deaths, and disability-adjusted life-years (DALYs) for nasopharyngeal carcinoma (NPC) from 1990 to 2021 globally, across 21 GBD regions and 204 countries and territories. As this study was a secondary analysis of a publicly available database, patient and public involvement was not applicable.

### Measures and definitions

Age-standardized rates (ASRs) were calculated for per 100,000 population. Temporal trends were analyzed these trends using a log-linear regression model, with the natural logarithm of the ASR as the dependent variable and calendar year as the independent variable (11):


y=α+βx+ϵ

where y=ln(ASR) and x=calendar year. The estimated annual percentage change (EAPC) was calculated from the β coefficient using the formula (12):


$$\text{EAPC}=100\times (e^{\beta }-1)$$

An EAPC value greater than zero indicates an increasing trend in age-standardized rates, whereas an EAPC value less than zero indicates a decreasing trend. In addition to estimating EAPC using log-linear regression, we further applied joinpoint regression to statistically identify significant changes in temporal trends. In addition to estimating EAPC using log-linear regression, we further applied joinpoint regression to statistically identify significant changes in temporal trends. For each segment, the annual percent change (APC) was computed, and the average annual percent change (AAPC) summarized the overall trend across the full study period. A total of 204 countries or territories and 21 GBD regions were selected in this study. The socio-demographic index (SDI) was estimated as a geometric mean of 0–1 indices of lag distributed income per capita, mean education of those ages 15 and older, and total fertility rate in females under 25 years (6). The SDI was scaled from 0 to 1, with higher values indicating higher socioeconomic levels, according to which regions were categorized into five SDI levels (low, low-middle, middle, high-middle, and high). For age group, we categorized the age groups into 5-year intervals starting from <5 to ≥95 years. We defined the aging-related ratio as the ratio of disease burden in individuals aged over 65 years to that under 65 years. Future trends (2022-2050) in age-standardized incidence, mortality, and DALY rates were projected using a Bayesian age-period-cohort (BAPC) modeling framework (13). This approach integrates temporal patterns in age, calendar period, and birth cohort while accounting for uncertainty through Bayesian inference. The model was implemented using the BAPC and INLA packages in R, following forecasting strategies established in recent GBD analyses. 95% uncertainty intervals were visualized as shaded bands around the projected trajectories in the figures.

### Statistical analysis

The statistical analyses were conducted using software R (version 4.4.1). Joinpoint analyses were conducted using the Joinpoint Regression Program (Version 5.4.0; National Cancer Institute), with differences between groups evaluated using the “Advanced analysis” – “Pairwise comparison procedure”. The data visualization was performed by using packages including ggplot2, heatmap, and so on. Data on incidence, deaths, DALYs, age-standardized incidence rate (ASIR), age-standardized death rate (ASDR), and age-standardized DALYs rate were presented with corresponding 95% uncertainty intervals (UIs). EAPCs were calculated with 95% confidence intervals (CIs). Values were displayed using bar charts and categorized by age, sex, and region subgroups. Annual trends were visualized using line graphs, and regional differences were depicted using heatmaps. Fitted curves were used to illustrate the associations and annual trends between SDI and disease burden indicators. Trends were described as “increasing” or “decreasing” when the slope was statistically significant; otherwise, they were described as “stable.” Statistical significance was defined as a two-sided *p* < 0.05.

## Results

### Global burden and temporal trends of NPC

Globally, the incident cases, deaths, and DALYs of NPC were 118,878 (95% UI: 104,836–135,884), 75,359 (95% UI: 67,515–83,706), and 2,490,191 (95% UI: 2,213,998–2,778,564), respectively, with an age-standardized incidence rate (ASIR), age-standardized death rate (ASDR), and age-standardized DALYs rate of 1.38 (1.22–1.58), 0.87 (0.78–0.97), and 28.91 (25.69–32.24), respectively (**Table 1**; **Supplementary Figures S1**-**S3**). However, the EAPCs of ASIR, ASDR, and age-standardized DALYs rate were −1.06 (-1.28 to -0.84), -2.17 (-2.37 to -1.98), and -2.29 (-2.49 to -2.09), respectively.

**Table 1:** Global burden of nasopharyngeal carcinoma and temporal trends from 1990 to 2021 by gender and regions.

Location	Incidence						Deaths						DALYs					
	1990		2021		1990-2021		1990		2021		1990-2021		1990		2021		1990-2021	
	Number	ASR (per 100,000)	Number	ASR (per 100,000)	EAPC (%)	AAPC (%)	Number	ASR (per 100,000)	Number	ASR (per 100,000)	EAPC (%)	AAPC (%)	Number	ASR (per 100,000)	Number	ASR (per 100,000)	EAPC (%)	AAPC (%)
Global	76256 (68714 - 83570)	1.735 (1.563 - 1.9)	118878 (104836 - 135884)	1.382 (1.22 - 1.579)	-1.058 (-1.276to-0.84)	-0.729 (-0.824 to -0.635)	64933 (58545 - 71096)	1.517 (1.369 - 1.66)	75359 (67515 - 83706)	0.871 (0.78 - 0.966)	-2.171 (-2.365to-1.977)	-1.799 (-1.885 to -1.713)	2361827 (2125353 - 2588450)	52.224 (46.993 - 57.284)	2490191 (2213998 - 2778564)	28.907 (25.689 - 32.235)	-2.287 (-2.486to-2.087)	-1.911 (-1.981 to -1.840)
Gender																		
Female	24936 (21366 - 28360)	1.102 (0.946 - 1.254)	32395 (27948 - 38077)	0.74 (0.639 - 0.871)	-1.6 (-1.811to-1.39)	-1.265 (-1.358 to -1.171)	21082 (18147 - 23917)	0.95 (0.818 - 1.077)	21422 (19066 - 24203)	0.478 (0.426 - 0.541)	-2.6 (-2.82to-2.38)	-2.179 (-2.298 to -2.059)	764133 (657465 - 871657)	33.119 (28.481 - 37.782)	701417 (624270 - 798171)	16.12 (14.342 - 18.384)	-2.71 (-2.931to-2.489)	-2.290 (-2.403 to -2.177)
Male	51320 (44302 - 57944)	2.418 (2.091 - 2.721)	86483 (73983 - 101789)	2.058 (1.764 - 2.416)	-0.846 (-1.065to-0.626)	-0.546 (-0.653 to -0.440)	43851 (37811 - 49404)	2.144 (1.848 - 2.412)	53937 (47076 - 61333)	1.299 (1.136 - 1.474)	-1.999 (-2.184to-1.813)	-1.638 (-1.755 to -1.521)	1597695 (1380769 - 1798792)	72.125 (62.347 - 81.308)	1788775 (1552540 - 2053374)	42.264 (36.695 - 48.458)	-2.099 (-2.289to-1.908)	-1.739 (-1.857 to -1.621)
SDI regions																		
Low SDI	3413 (2797 - 4065)	1.215 (0.996 - 1.446)	5948 (4838 - 7360)	0.925 (0.761 - 1.129)	-1.087 (-1.234to-0.94)	-0.887 (-0.992 to -0.782)	3322 (2715 - 3944)	1.215 (0.994 - 1.442)	5617 (4570 - 6912)	0.913 (0.751 - 1.114)	-1.118 (-1.26to-0.976)	-0.929 (-1.038 to -0.819)	129546 (106256 - 153557)	41.558 (34.023 - 49.475)	217575 (174821 - 272204)	30.292 (24.578 - 37.467)	-1.249 (-1.384to-1.114)	-1.016 (-1.106 to -0.926)
Low-middle SDI	8877 (7609 - 10362)	1.198 (1.031 - 1.399)	15717 (13901 - 17756)	0.968 (0.861 - 1.091)	-0.763 (-0.886to-0.64)	-0.674 (-0.775 to -0.573)	8516 (7317 - 9928)	1.184 (1.019 - 1.384)	14599 (12930 - 16491)	0.925 (0.824 - 1.042)	-0.872 (-0.985to-0.758)	-0.779 (-0.885 to -0.673)	327415 (280590 - 380157)	40.497 (34.732 - 47.226)	522924 (457946 - 593572)	30.784 (27.1 - 34.865)	-0.951 (-1.06to-0.842)	-0.877 (-1.011 to -0.743)
Middle SDI	30883 (27533 - 34643)	2.499 (2.236 - 2.8)	44245 (38358 - 51082)	1.584 (1.374 - 1.826)	-1.831 (-2.128to-1.533)	-1.458 (-1.537 to -1.378)	27071 (24252 - 30232)	2.301 (2.062 - 2.566)	28840 (25418 - 32900)	1.044 (0.923 - 1.188)	-2.965 (-3.212to-2.717)	-2.535 (-2.664 to -2.407)	1000200 (893743 - 1113965)	76.215 (68.179 - 85.013)	935643 (821058 - 1065446)	33.133 (29.124 - 37.664)	-3.088 (-3.342to-2.834)	-2.659 (-2.777 to -2.541)
High-middle SDI	24071 (20655 - 27723)	2.305 (1.981 - 2.653)	41734 (33972 - 51213)	2.383 (1.937 - 2.93)	-0.361 (-0.668to-0.053)	0.108 (-0.046 - 0.262)	19893 (17137 - 22771)	1.935 (1.669 - 2.212)	19752 (16571 - 23679)	1.039 (0.873 - 1.247)	-2.597 (-2.832to-2.361)	-2.011 (-2.128 to -1.893)	703109 (604593 - 805685)	66.62 (57.268 - 76.201)	631285 (524546 - 761529)	34.84 (29.045 - 41.643)	-2.722 (-2.977to-2.466)	-2.080 (-2.201 to -1.958)
High SDI	8981 (8668 - 9333)	0.882 (0.851 - 0.917)	11181 (10415 - 12023)	0.706 (0.661 - 0.757)	-0.873 (-0.979to-0.768)	-0.694 (-0.813 to -0.576)	6104 (5881 - 6355)	0.585 (0.564 - 0.609)	6505 (5986 - 7037)	0.351 (0.325 - 0.379)	-1.872 (-1.948to-1.795)	-1.658 (-1.740 to -1.575)	200614 (192993 - 209206)	19.886 (19.135 - 20.743)	181317 (168521 - 195018)	11.106 (10.343 - 11.918)	-2.112 (-2.19to-2.034)	-1.881 (-1.980 to -1.782)
GBD regions																		
Central Asia	199 (177 - 225)	0.376 (0.333 - 0.429)	364 (305 - 439)	0.404 (0.34 - 0.485)	0.19 (-0.009-0.39)	0.237 (0.082 - 0.394)	191 (169 - 216)	0.37 (0.327 - 0.429)	336 (284 - 405)	0.384 (0.326 - 0.461)	0.069 (-0.116-0.254)	0.120 (-0.026 - 0.266)	7394 (6539 - 8432)	12.942 (11.472 - 14.646)	12238 (10175 - 15023)	13.038 (10.859 - 15.98)	-0.068 (-0.248-0.113)	0.031 (-0.173 - 0.236)
Central Europe	594 (561 - 633)	0.409 (0.385 - 0.436)	809 (718 - 916)	0.448 (0.4 - 0.51)	0.312 (-0.084-0.71)	0.298 (0.143 - 0.455)	563 (530 - 601)	0.386 (0.363 - 0.412)	720 (640 - 812)	0.37 (0.33 - 0.42)	-0.145 (-0.52-0.232)	-0.153 (-0.297 to -0.010)	18570 (17459 - 19811)	12.881 (12.096 - 13.763)	20909 (18617 - 23648)	11.869 (10.545 - 13.461)	-0.289 (-0.683-0.106)	-0.284 (-0.445 to -0.123)
Eastern Europe	896 (827 - 1034)	0.331 (0.305 - 0.382)	1007 (888 - 1149)	0.324 (0.286 - 0.371)	-0.472 (-0.692to-0.251)	0.127 (-0.594 - 0.854)	874 (805 - 1005)	0.321 (0.296 - 0.37)	962 (850 - 1103)	0.299 (0.264 - 0.343)	-0.66 (-0.894to-0.426)	-0.032 (-0.720 - 0.660)	29840 (27507 - 34571)	11.29 (10.43 - 13.071)	30301 (26559 - 34795)	10.169 (8.906 - 11.709)	-0.785 (-1.026to-0.543)	-0.157 (-0.889 - 0.581)
Australasia	273 (244 - 305)	1.218 (1.082 - 1.36)	316 (246 - 395)	0.763 (0.591 - 0.958)	-1.683 (-1.874to-1.49)	-1.525 (-1.968 to -1.081)	114 (103 - 126)	0.501 (0.452 - 0.554)	115 (91 - 143)	0.24 (0.189 - 0.298)	-2.5 (-2.58to-2.421)	-2.387 (-2.652 to -2.121)	3583 (3210 - 3983)	16.064 (14.381 - 17.865)	3311 (2596 - 4110)	7.729 (6.004 - 9.627)	-2.463 (-2.546to-2.38)	-2.354 (-2.641 to -2.066)
High-income Asia Pacific	851 (807 - 893)	0.418 (0.396 - 0.439)	1484 (1358 - 1599)	0.418 (0.386 - 0.452)	-0.146 (-0.498-0.207)	-0.037 (-0.364 - 0.291)	744 (704 - 782)	0.367 (0.347 - 0.385)	1350 (1214 - 1454)	0.312 (0.287 - 0.335)	-0.833 (-1.142to-0.524)	-0.537 (-0.740 to -0.334)	23956 (22710 - 25156)	11.714 (11.101 - 12.299)	29562 (27252 - 31603)	8.577 (8.012 - 9.247)	-1.311 (-1.618to-1.003)	-1.011 (-1.191 to -0.831)
High-income North America	2175 (2106 - 2251)	0.691 (0.669 - 0.714)	2633 (2498 - 2760)	0.513 (0.488 - 0.537)	-1.094 (-1.16to-1.027)	-0.972 (-1.178 to -0.765)	1131 (1089 - 1170)	0.347 (0.335 - 0.358)	1201 (1123 - 1265)	0.202 (0.191 - 0.213)	-1.879 (-1.964to-1.793)	-1.765 (-1.932 to -1.599)	35158 (34029 - 36300)	11.291 (10.936 - 11.66)	34681 (32890 - 36373)	6.483 (6.16 - 6.794)	-1.918 (-2.004to-1.831)	-1.807 (-1.989 to -1.626)
Southern Latin America	167 (147 - 189)	0.36 (0.317 - 0.407)	152 (121 - 189)	0.185 (0.147 - 0.231)	-1.832 (-1.967to-1.696)	-2.094 (-2.386 to -1.800)	159 (140 - 180)	0.347 (0.305 - 0.392)	134 (107 - 169)	0.159 (0.127 - 0.2)	-2.185 (-2.326to-2.043)	-2.461 (-2.802 to -2.118)	4992 (4413 - 5645)	10.617 (9.393 - 12.012)	3803 (3042 - 4758)	4.725 (3.776 - 5.931)	-2.293 (-2.424to-2.163)	-2.579 (-2.854 to -2.304)
Western Europe	3510 (3348 - 3694)	0.719 (0.685 - 0.759)	3184 (2872 - 3558)	0.485 (0.441 - 0.542)	-1.353 (-1.478to-1.229)	-1.300 (-1.655 to -0.943)	2547 (2429 - 2685)	0.491 (0.468 - 0.517)	1893 (1709 - 2121)	0.236 (0.214 - 0.263)	-2.455 (-2.514to-2.396)	-2.402 (-2.580 to -2.223)	78663 (75054 - 82813)	16.239 (15.468 - 17.125)	50535 (45877 - 56461)	7.333 (6.68 - 8.183)	-2.684 (-2.748to-2.619)	-2.586 (-2.806 to -2.366)
Andean Latin America	36 (31 - 40)	0.154 (0.136 - 0.172)	79 (64 - 99)	0.13 (0.105 - 0.162)	-0.498 (-0.601to-0.394)	-0.432 (-0.917 - 0.055)	35 (31 - 40)	0.16 (0.142 - 0.179)	75 (61 - 93)	0.126 (0.102 - 0.156)	-0.721 (-0.832to-0.61)	-0.668 (-1.164 to -0.169)	1229 (1086 - 1387)	4.701 (4.143 - 5.284)	2158 (1730 - 2675)	3.466 (2.772 - 4.298)	-0.981 (-1.1to-0.862)	-0.918 (-1.385 to -0.450)
Caribbean	124 (112 - 136)	0.457 (0.415 - 0.5)	282 (239 - 332)	0.53 (0.449 - 0.62)	0.563 (0.441-0.684)	0.558 (0.339 - 0.778)	120 (108 - 131)	0.455 (0.413 - 0.497)	268 (226 - 316)	0.499 (0.423 - 0.588)	0.392 (0.285-0.499)	0.394 (0.216 - 0.572)	3822 (3394 - 4189)	13.391 (11.953 - 14.681)	7869 (6642 - 9391)	14.895 (12.598 - 17.741)	0.429 (0.316-0.541)	0.556 (0.443 - 0.670)
Central Latin America	219 (208 - 231)	0.228 (0.216 - 0.24)	480 (420 - 549)	0.187 (0.164 - 0.214)	-0.943 (-1.074to-0.811)	-0.682 (-0.804 to -0.560)	210 (199 - 221)	0.231 (0.219 - 0.243)	441 (385 - 503)	0.174 (0.152 - 0.199)	-1.213 (-1.345to-1.081)	-0.965 (-1.079 to -0.851)	7433 (7054 - 7838)	6.858 (6.523 - 7.201)	13360 (11675 - 15312)	5.128 (4.482 - 5.875)	-1.248 (-1.371to-1.124)	-1.006 (-1.128 to -0.883)
Tropical Latin America	220 (206 - 238)	0.198 (0.185 - 0.214)	614 (565 - 669)	0.237 (0.218 - 0.258)	0.254 (-0.176-0.686)	0.544 (0.166 - 0.923)	202 (189 - 219)	0.189 (0.176 - 0.204)	537 (493 - 586)	0.207 (0.189 - 0.225)	-0.027 (-0.458-0.406)	0.272 (-0.059 - 0.605)	8015 (7472 - 8661)	6.721 (6.264 - 7.254)	18744 (17262 - 20431)	7.245 (6.675 - 7.901)	-0.057 (-0.496-0.384)	0.202 (-0.173 - 0.578)
North Africa and Middle East	1703 (1480 - 1903)	0.832 (0.724 - 0.938)	3435 (2955 - 3982)	0.64 (0.552 - 0.739)	-0.956 (-0.993to-0.919)	-0.836 (-0.892 to -0.781)	1577 (1368 - 1768)	0.813 (0.707 - 0.92)	2768 (2376 - 3191)	0.555 (0.477 - 0.639)	-1.362 (-1.402to-1.322)	-1.223 (-1.308 to -1.138)	60297 (52169 - 67213)	26.494 (22.939 - 29.628)	96626 (82631 - 112038)	17.241 (14.773 - 19.9)	-1.511 (-1.545to-1.476)	-1.385 (-1.433 to -1.338)
South Asia	9944 (8573 - 11504)	1.388 (1.195 - 1.608)	17031 (14913 - 19127)	1.028 (0.902 - 1.154)	-1.16 (-1.303to-1.017)	-0.952 (-1.085 to -0.819)	9559 (8239 - 11056)	1.375 (1.181 - 1.591)	15784 (13862 - 17729)	0.977 (0.861 - 1.098)	-1.285 (-1.42to-1.15)	-1.079 (-1.216 to -0.942)	373203 (321842 - 430997)	47.582 (41.007 - 55.072)	567907 (495773 - 640475)	32.962 (28.881 - 37.083)	-1.361 (-1.487to-1.236)	-1.175 (-1.307 to -1.042)
East Asia	46602 (39765 - 53748)	4.634 (3.955 - 5.328)	68039 (55299 - 83521)	3.409 (2.771 - 4.19)	-1.499 (-1.892to-1.104)	-0.987 (-1.180 to -0.795)	38835 (33115 - 44796)	4.08 (3.483 - 4.68)	32616 (26795 - 39615)	1.516 (1.25 - 1.833)	-3.801 (-4.096to-3.505)	-3.179 (-3.311 to -3.047)	1403841 (1194223 - 1621286)	133.453 (113.609 - 154.099)	1024646 (837975 - 1254065)	49.004 (40.124 - 59.481)	-3.865 (-4.188to-3.541)	-3.208 (-3.313 to -3.103)
Oceania	35 (25 - 47)	0.973 (0.691 - 1.302)	74 (52 - 105)	0.796 (0.563 - 1.131)	-0.612 (-0.695to-0.53)	-0.631 (-0.790 to -0.472)	33 (23 - 44)	0.951 (0.676 - 1.265)	68 (47 - 98)	0.773 (0.548 - 1.107)	-0.628 (-0.713to-0.542)	-0.647 (-0.839 to -0.454)	1171 (803 - 1591)	30.097 (21.022 - 40.644)	2426 (1670 - 3532)	24.376 (16.888 - 35.225)	-0.645 (-0.736to-0.554)	-0.660 (-0.863 to -0.457)
Southeast Asia	6209 (5433 - 7106)	2.03 (1.773 - 2.321)	13661 (11880 - 15506)	1.889 (1.648 - 2.143)	-0.387 (-0.451to-0.323)	-0.236 (-0.314 to -0.158)	5608 (4897 - 6406)	1.935 (1.691 - 2.21)	11177 (9797 - 12662)	1.599 (1.412 - 1.811)	-0.756 (-0.812to-0.701)	-0.622 (-0.697 to -0.547)	206433 (180099 - 235782)	62.807 (54.843 - 71.854)	376855 (327440 - 429623)	50.768 (44.297 - 57.718)	-0.839 (-0.902to-0.776)	-0.688 (-0.800 to -0.576)
Central Sub-Saharan Africa	109 (84 - 140)	0.403 (0.312 - 0.523)	252 (182 - 353)	0.366 (0.265 - 0.515)	-0.328 (-0.405to-0.251)	-0.315 (-0.401 to -0.230)	107 (83 - 139)	0.414 (0.322 - 0.537)	243 (176 - 341)	0.372 (0.27 - 0.528)	-0.356 (-0.427to-0.285)	-0.344 (-0.425 to -0.263)	3932 (3043 - 5033)	12.924 (9.979 - 16.714)	8990 (6469 - 12702)	11.488 (8.316 - 16.137)	-0.385 (-0.458to-0.313)	-0.393 (-0.498 to -0.288)
Eastern Sub-Saharan Africa	1573 (1245 - 1885)	1.647 (1.308 - 1.976)	3220 (2365 - 4262)	1.408 (1.05 - 1.842)	-0.71 (-0.784to-0.636)	-0.505 (-0.570 to -0.441)	1527 (1206 - 1829)	1.649 (1.31 - 1.978)	3018 (2226 - 3992)	1.387 (1.039 - 1.817)	-0.756 (-0.826to-0.686)	-0.557 (-0.622 to -0.491)	60552 (47578 - 72380)	56.009 (44.094 - 67.236)	121544 (88099 - 162332)	46.696 (34.463 - 61.776)	-0.804 (-0.879to-0.73)	-0.587 (-0.645 to -0.530)
Southern Sub-Saharan Africa	171 (146 - 201)	0.551 (0.468 - 0.652)	357 (315 - 400)	0.552 (0.49 - 0.615)	-0.057 (-0.301-0.187)	0.011 (-0.098 - 0.120)	164 (140 - 195)	0.552 (0.467 - 0.656)	340 (301 - 381)	0.546 (0.486 - 0.606)	-0.108 (-0.368-0.152)	-0.073 (-0.482 - 0.338)	5823 (4998 - 6863)	17.217 (14.678 - 20.458)	11590 (10099 - 13170)	16.895 (14.845 - 19.017)	-0.105 (-0.364-0.155)	-0.145 (-0.481 - 0.193)
Western Sub-Saharan Africa	647 (502 - 795)	0.616 (0.483 - 0.753)	1405 (954 - 1860)	0.532 (0.374 - 0.693)	-0.57 (-0.684to-0.456)	-0.494 (-0.564 to -0.424)	631 (494 - 773)	0.618 (0.488 - 0.755)	1312 (905 - 1726)	0.522 (0.374 - 0.672)	-0.644 (-0.753to-0.536)	-0.567 (-0.634 to -0.500)	23920 (18588 - 29546)	20.741 (16.204 - 25.557)	52137 (34799 - 69490)	17.653 (12.122 - 23.257)	-0.615 (-0.73to-0.501)	-0.536 (-0.609 to -0.463)

ASR, age-standardized rate; EAPC, estimated annual percentage change; AAPC, average annual percent change.

**Figure 1:**
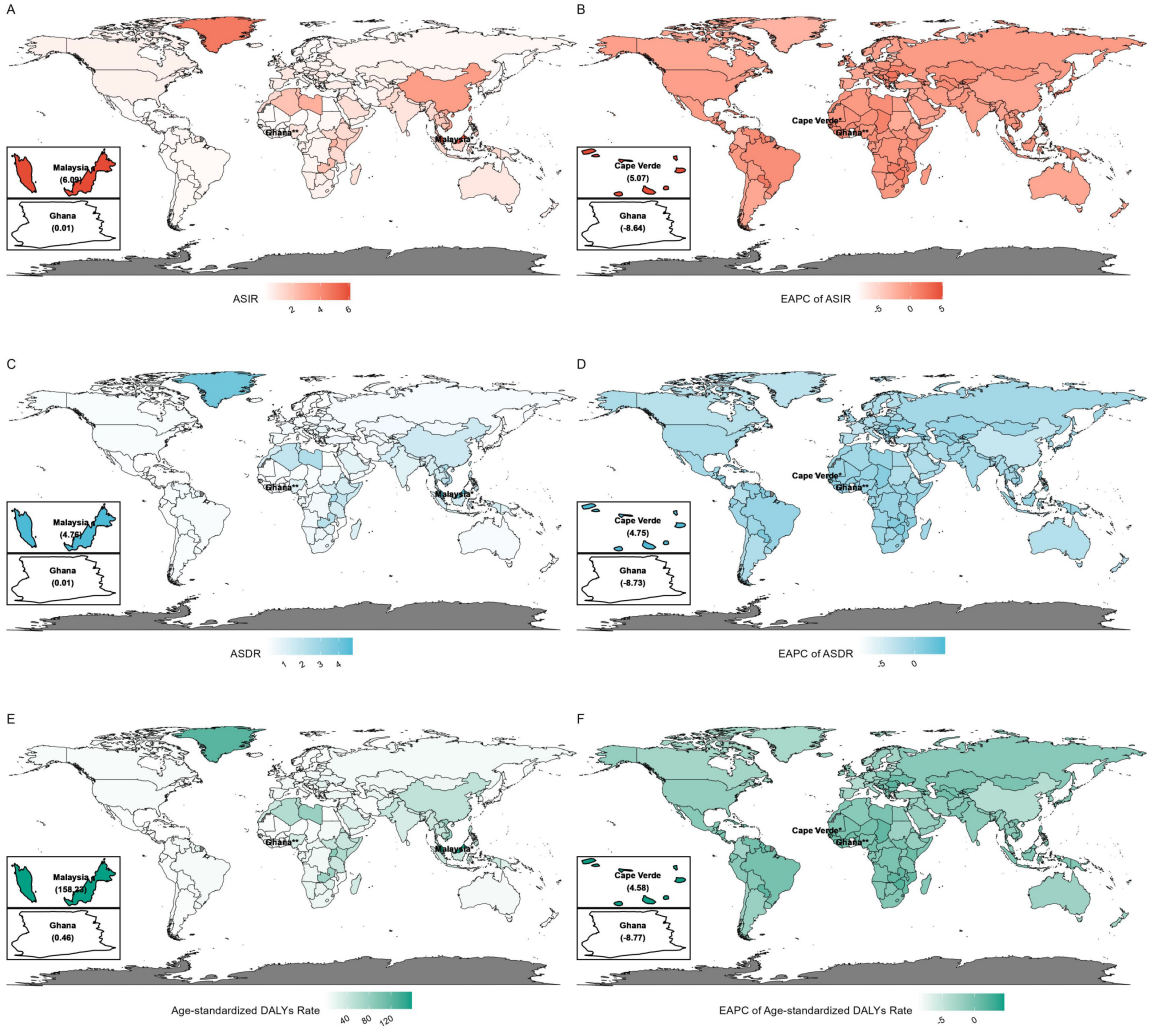
Global distribution and temporal trends of nasopharyngeal carcinoma burden in 2021. **(A)** Age-standardized incidence rate (ASIR); **(B)** estimated annual percentage change (EAPC) of ASIR; **(C)** age-standardized death rate (ASDR); **(D)** EAPC of ASDR; **(E)** age-standardized DALY rate; **(F)** EAPC of age-standardized DALYs rate.

As shown in **Table 1**, the ASIR was highest in East Asia at 3.41 (2.77–4.19), whereas the highest ASDR was observed in Southeast Asia at 1.60 (1.41–1.81). The highest EAPCs of ASIR, ASDR, and age-standardized DALYs rate were observed in the Caribbean at 0.56 (0.44–0.68), 0.39 (0.29–0.50), and 0.43 (0.32–0.54), respectively. **Figure 1** presents the global distribution of ASIR, ASDR, age-standardized DALYs rate, and their corresponding EAPCs. At the national level, Malaysia exhibited the highest ASIR at 6.09 (5.21–7.19), ASDR at 4.76 (4.10–5.62), and age-standardized DALYs rate at 158.23 (134.38–187.14). The highest EAPCs for ASIR, ASDR, and age-standardized DALYs rate were observed in Cape Verde at 5.07 (3.55–6.62), 4.75 (3.22–6.32), and 4.58 (3.02–6.15), respectively (**Supplementary Table S1**).

### Burden and temporal trends of NPC by SDI regions

**Figure 2:**
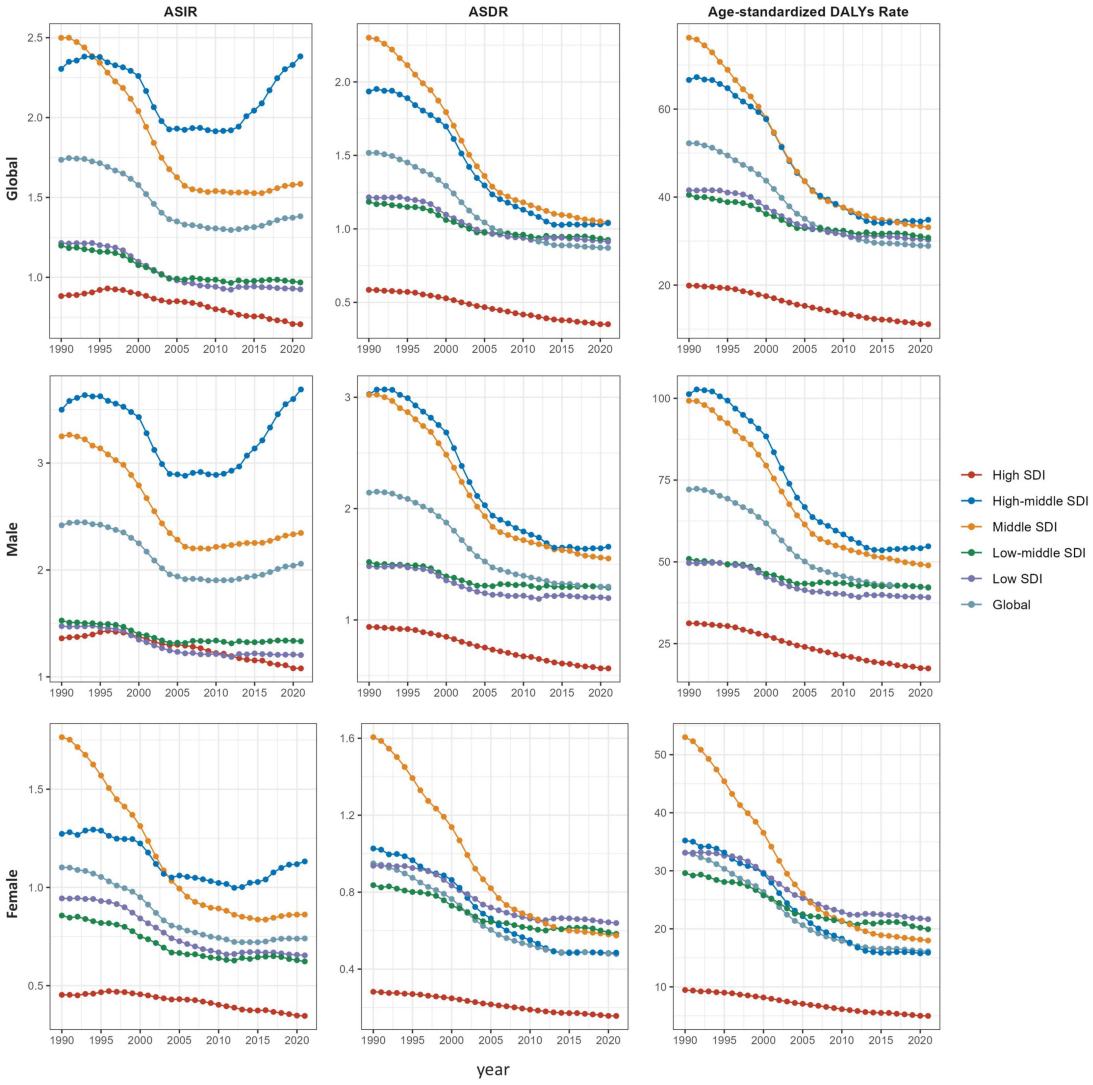
Trends in age-standardized rates of nasopharyngeal carcinoma by socio-demographic index (SDI) level and sex, 1990–2021.

In 2021, the highest global ASIR (2.38, 1.94–2.93) and age-standardized DALYs rate (34.84, 29.05–41.64) for NPC were observed in high-middle SDI regions in 2021(**Figure 2**). Middle SDI regions had the highest ASDR (1.04, 0.92–1.19) but the lowest EAPCs for ASDR and age-standardized DALYs rate at −2.97 (−3.21 to −2.72) and −3.09 (−3.34 to −2.83), respectively. By contrast, high-SDI regions exhibited the lowest ASIR, ASDR, and age-standardized DALYs rate in 2021 at 0.71 (0.66-0.76), 0.35 (0.33–0.38), and 11.11 (10.34–11.92), respectively.

Across the SDI spectrum, East Asia consistently presented higher ASIRs. Age-standardized DALYs rates showed a significant negative correlation with SDI (R = −0.16, *p* = 0.024) (**Supplementary Figure S4**).

### Burden and temporal trends of NPC by gender and age

In 2021, the ASIR, ASDR, and age-standardized DALYs rate in males were 2.06 (1.76-2.42), 1.30 (1.14-1.47), and 42.26 (36.70-48.46), respectively, with EAPCs of -0.85 (-1.07 to -0.63), -2.00 (-2.18 to -1.81), and -2.10 (-2.29 to -1.91). In females, the ASIR, ASDR, and age-standardized DALYs rate were 0.74 (0.64–0.87), 0.48 (0.43–0.54), and 16.12 (14.34–18.38), respectively, with EAPCs of −1.60 (−1.91 to −1.39), −2.60 (−2.82 to −2.38), and −2.71 (−2.93 to −2.49) (**Table 1**).

Significant sex differences in AAPCs were observed across all measures within each SDI region (*p* < 0.001) (**Supplementary Table S2**; **Supplementary Figures S5**-**S7**). The lowest ASIR, ASDR, and age-standardized DALYs rates occurred in high-SDI regions for both sexes. However, high-middle SDI regions showed a clear upward incidence trend, with an APC of 1.64 (95% CI: 1.30–1.99, *p* < 0.01) in females during 2013–2021 and APC 2.74 (95% CI: 2.57–2.92, *p* < 0.01) in males during 2012–2021 (**Figure 2**; **Supplementary Figures S5**-**S7**; **Supplementary Table S3**). ASDR and age-standardized DALYs rates in females showed significant negative correlations with SDI (R = −0.23 and −0.25, respectively; both *p* < 0.001) (**Supplementary Figure S8**).

Globally, the highest incidence rate was observed in individuals aged 65–69 years at 4.23 (3.66–4.90), the highest death rate was in those aged 85–89 years at 4.17 (3.55–4.66), and the highest DALYs rate in individuals aged 55–59 years at 90.17 (80.03–100.91) (**Supplementary Table S4**). From 1990 to 2021, the global rates of NPC incidence, death, and DALYs rates generally declined across age groups (**Supplementary Figure S9**).

**Figure 3:**
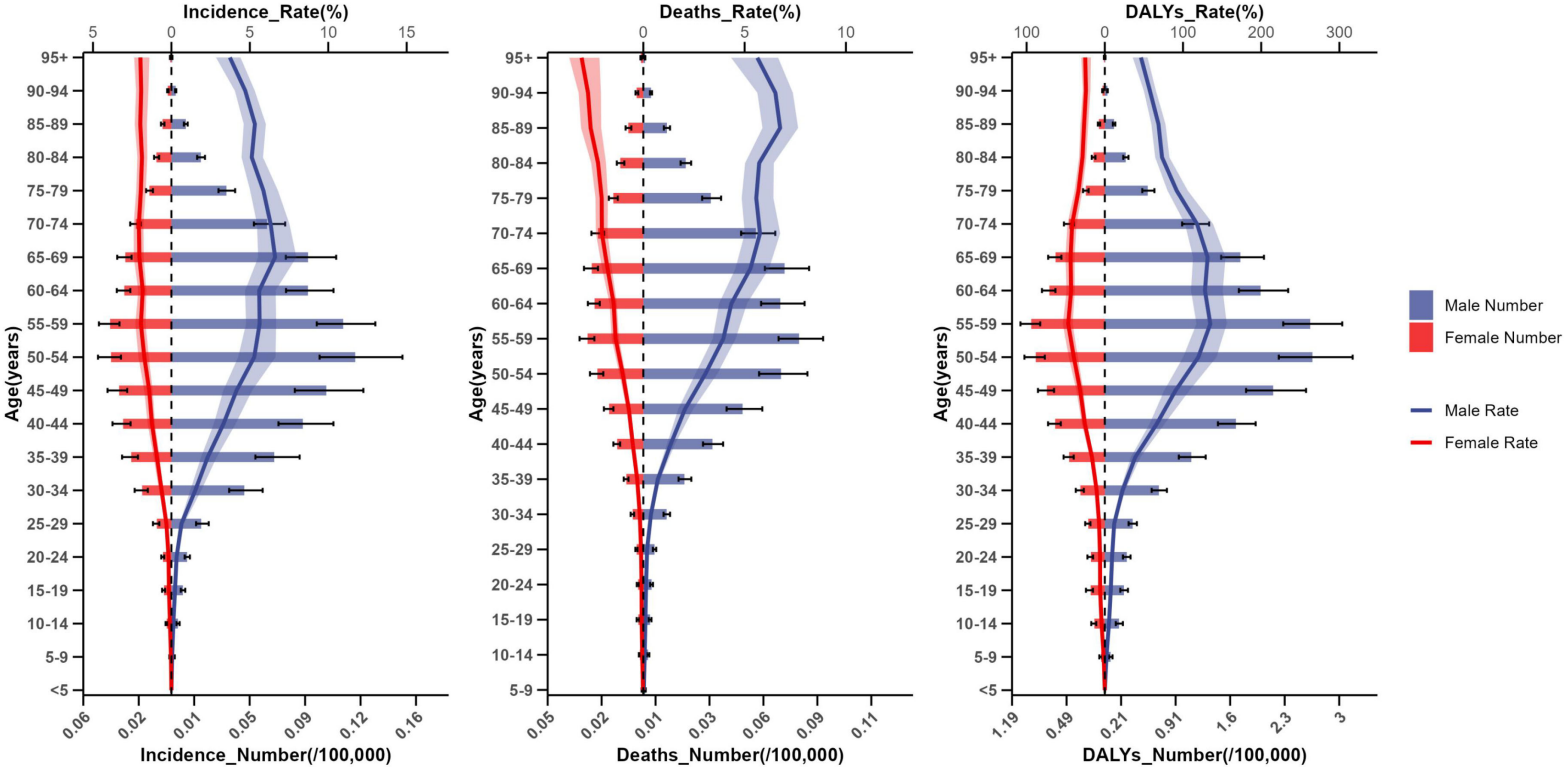
Number and rate for incidence, deaths, and disability-adjusted life years (DALYs) of nasopharyngeal carcinoma across different gender and age groups in 2021.

When stratified by sex, males showed the highest incidence rate at ages 65–69 years (6.61, 5.53–7.97), the highest death rate at ages 85–95 years (6.75, 5.88–7.64), and the highest DALYs rate at ages 55–59 years (134.92, 117.18–155.93). In females, the highest incidence rate was observed at ages 70–74 years (2.07, 1.78–2.40), the highest death rate at ages ≥95 years (3.05, 2.17–3.65), and the highest DALYs rate at ages 55–59 years (46.82, 41.11–53.65) (**Figure 3**).

From 1990 to 2021, the ratio of male-to-female ratio of NPC incidence increased significantly among individuals aged 50–54 years (*p* < 0.05), while the ratios for deaths and DALYs increased significantly among those aged 25–54 years, except for ages 40–44 years (*p* < 0.05) (**Supplementary Figure S10**). Furthermore, Japan exhibited the highest aging-related ratios for incidence (3.94), deaths (5.11 times), and DALYs (4.44 times) between 1990 and 2021 (**Supplementary Figure S11**).

### Predictions of NPC from 2022 to 2050

**Figure 4:**
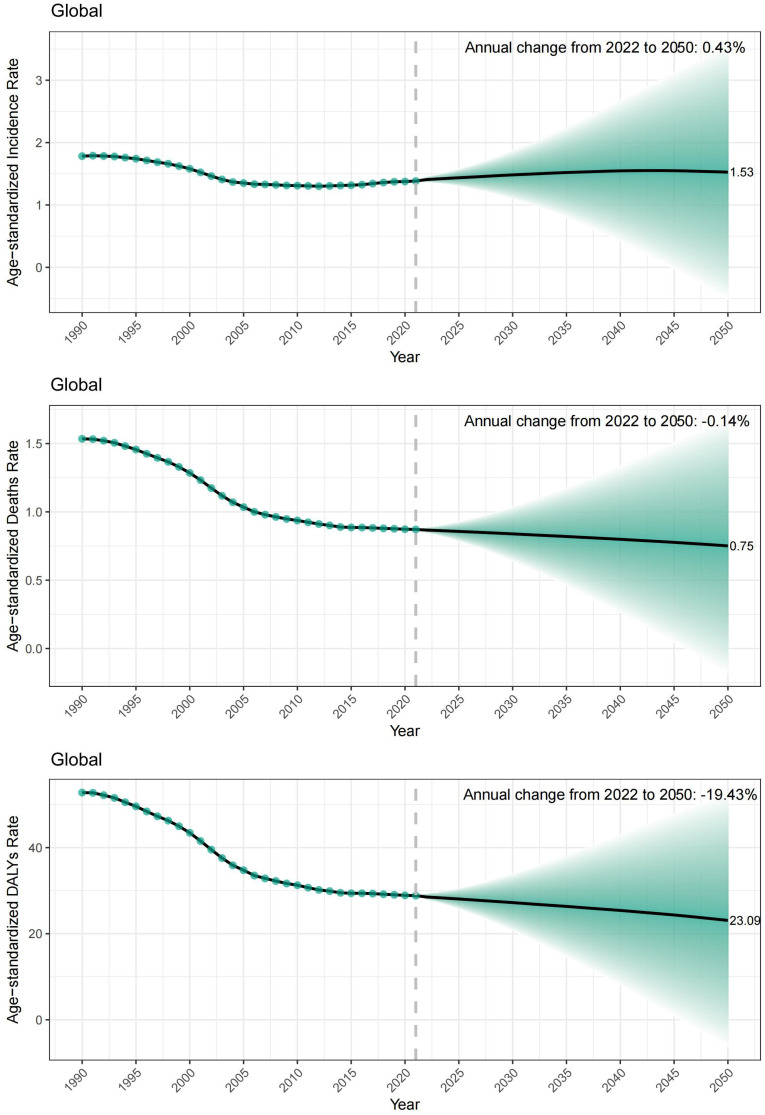
Observed and projected trends in nasopharyngeal carcinoma burden, 1990–2050. Green dots indicate global estimates from 1990 to 2021; the dotted line marks the start of projections (2022); shaded areas represent the 95% uncertainty intervals (UI).

We projected ASIR, ASDR, and age-standardized DALYs rate for NPC over the next 30 years by using a Bayesian age–period–cohort (BAPC) model. The results indicate that the global ASIR is expected to reach 1.53 per 100,000 population by 2050, representing an annual increase of 0.43% (**Figure 4**). This increasing trend is primarily driven by males, whose ASIR is projected to increase by male ASIR, which increasing 0.6% annually, whereas the annual change in ASIR among females is projected to be −0.1% in female (**Supplementary Figure S12**).

In contrast, the ASDR and age-standardized DALYs rates are estimated to decline to 0.75 and 23.09 per 100,000 population, respectively, by 2050, with annual decreases of −0.14% and −1.94%, respectively.

## Discussion

Using the latest GBD 1990–2021 data, this study offers a comprehensive global assessment of NPC burden across SDI, age, and sex, identifies key epidemiological shifts, and projects trends through 2050. Although the ASIR of NPC in 2021 appears to have decreased significantly compared with 1990, previous studies consistently reported similar declines across children, young adults, middle-aged, and older populations (7, 14). Our results indicate that the ASIR trend is predominantly driven by males, which may contribute to a continued global increase in the future. Consistent with the previous studies, the ASDR and age-standardized DALYs rate of NPC have declined over the past decades (2, 15). Furthermore, the present research projected the continuation of these decreasing trends in the future. Overall, the significant decline is likely attributed to advancements in diagnostic and therapeutic technologies. In part, although the EBV infection remains widespread, advances in detection technology have substantially improved the sensitivity and predictive value of tests for anti-EBV antibodies and plasma or nasopharyngeal EBV DNA, including through fragment size and methylation-based analysis, even in asymptomatic period (16, 17). Moreover, substantial investment in EBV-based screening and heightened awareness within health systems have enabled earlier detection of NPC (17). Additionally, the oral–nasopharyngeal microenvironment plays an increasingly recognized role in NPC development (18). Alterations in the oral–nasopharyngeal microbiome may influence local immune responses and facilitate EBV reactivation, thereby elevating NPC risk (19). Conversely, improvements in oral health and microbial balance may help attenuate these pathways, providing a plausible mechanism linking environmental changes to reductions in NPC incidence (20). High-SDI regions tend to have a lower burden of oral disorders, potentially contributing to reduced NPC incidence (21). At the 2022 World Health Assembly, member states adopted a global strategy targeting universal oral health coverage for all populations by 2030 (22). Together, these efforts create a critical opportunity to strengthen care in a historically overlooked field and support broader NPC prevention (23). Furthermore, advances in diagnostic imaging technology, accompanied by intensity-modulated radiotherapy (IMRT) and optimization of chemotherapy regimens, have significantly improved early diagnosis and survival rates for NPC (24, 25).

Our findings highlight enduring sex disparities in NPC, with males experiencing a disproportionately higher morbidity globally and in future estimates. Furthermore, the decreasing burden trend in males was far slower than in females (26). After adjustment for major risk factors, previous studies have consistently documented sex disparities in cancer incidence and survival (27). These differences may reflect sex-specific risk exposures, including older age at diagnosis, more advanced stage at presentation, more aggressive tumor biology, and suboptimal treatment (28, 29). Risky behaviors such as tobacco and alcohol use (30), which are more prevalent among males, may further contribute to these disparities. Moreover, sex hormones and sex chromosomes are potential mechanisms driving female survival advantages (31).

A comprehensive analysis of 2021 GBD-observed NPC still showed regional variations (8). Significant differences in the cancer spectrum among regions can be attributed to living habits and unhealthy diets. Findings from the International Tobacco Control (ITC) Project indicate that Malaysia has a high level of cigarette tax avoidance or evasion (39.7%) (32), which is higher than in other Asian countries and may in turn contribute to higher smoking rates (37.5% in 15–64 years males) (33). Malaysia has introduced measures to reduce tobacco use and secondhand smoke exposure in line with the Framework Convention on Tobacco Control, ratified in 2005 (34). Greenland faces comparable challenges, with harmful alcohol use and rising obesity driven by dietary shifts from marine to domestic animal products and decreased physical activity (35, 36). The government has addressed these public health issues through its preventive programs, Inuuneritta I and Inuuneritta II (37).

Moreover, despite overall declines in morbidity and mortality, NPC incidence and mortality remain disproportionately high in East Asia. Regions with high ASIR and high-ASDR regions are therefore more likely to prioritize disease research and attract greater governmental and health-system attention, facilitating public awareness and advancement of screening technologies. Similar measures may be needed in the Caribbean, which is projected to experience an increasing NPC burden by 2021. In this region, much of the cancer burden is driven by tobacco (including SHS) and alcohol use, and a steady increase in metabolic diseases (30, 38). To address these challenges, the first edition of the Latin America and Caribbean Code Against Cancer was developed in collaboration with the Pan-American Health Organization. This code includes evidence-based, individual-level cancer prevention recommendations targeted at the general population (39). Cape Verde has exhibited a continuous increasing trend in NPC prevalence, however, this phenomenon has not attracted widespread attention. As in many African countries, the absence of a national screening program, insufficient attention, and lack of dedicated cancer treatment facilities (unavailability and high cost of high sensitivity screening tests), make cancer screening far more difficult than it should be, contributing to the region’s high mortality rates (40, 41). With changes in EBV exposure patterns, including improved oral and upper respiratory hygiene, lower childhood infection risk (42), and overall improvements in living and socioeconomic conditions (43), several historically high-incidence regions, such as Singapore, have experienced declines in NPC rates. Beyond smoking and alcohol, long-term dietary and environmental improvements—such as reduced consumption of Cantonese-style salted fish following widespread refrigeration and better air quality—may also have contributed to declining NPC incidence among younger cohorts in endemic regions (44, 45).

Although early diagnosis, behavioral change, and technological advances have reduced the burden of NPC, recurrence or metastasis (R/M) and population ageing remain major barriers to cure, with 20–30% of patients with advanced disease experiencing treatment failure due to R/M (46). Immunotherapy, which enhances antitumor immunity by modulating immune effector cells, has shown safety and efficacy in recent clinical studies (47). Furthermore, a higher age-related ratio indicates that older adults may have a more significant impact on the incidence and mortality rate of NPC. This age-driven burden has become more prominent in Japan and Singapore. Japan has undergone pronounced population ageing over recent decades, reflected in a substantial rise in life expectancy—from 79.4 years in 1990 to 85.2 years in 2021 (48). Singapore faces a similar ageing challenge (49). Population aging may increase cancer-related mortality and complicate treatment because of coexisting conditions, posing substantial challenges to both medical and community care systems. Preparation for an aging—and increasingly super-aged—society is therefore crucial, not only from healthcare and resource-planning perspectives but also from broader socioeconomic perspectives.

The present study uses the latest datasets to update and project evidence on the global NPC burden of NPC from 1990 to 2050. These findings provide valuable insights for estimating future disease trends and informing strategies aimed at improving health outcomes.

This study also has limitations. First, GBD estimates rely on modeling assumptions and data harmonization, which may introduce uncertainty (50). Second, cancer registry coverage remains incomplete in several low-SDI regions, potentially affecting estimate accuracy despite correction procedures (6). Third, long-term projections based on the BAPC inherent uncertainty, as future demographic and epidemiological changes may diverge from historical patterns. These factors should be considered when interpreting the results.

## Conclusion

Although global incidence, mortality, and disability burden from nasopharyngeal carcinoma have declined since 1990, the past decade has been marked by a rising ASIR, particularly among males. East Asia, high-middle socio-demographic index regions, and aging populations continue to experience disproportionately high burden. Strengthening standardized management strategies and implementing targeted prevention efforts in these settings are essential to mitigate future increases in NPC burden.

## Data Availability

The original contributions presented in the study are included in the article/**Supplementary Material**. Further inquiries can be directed to the corresponding authors.

## Supplementary material

Supplementary Table 1 Global burden of nasopharyngeal carcinoma and temporal trends from 1990 to 2021 across 204 countries or territories.

Supplementary Table 2 Average annual percent change (AAPC) estimates derived from joinpoint regression using group comparison across the five socio-demographic index (SDI) regions, stratified by sex.

Supplementary Table 3 Age-standardized incidence, mortality, and disability-adjusted life years (DALYs) rates across SDI regions, 1990–2023, with annual percentage changes (APC) by sex.

Supplementary Table 4 Peak age groups and corresponding incidence, deaths, and disability-adjusted life years (DALYs) rates in 2021 across major regions.

Supplementary Figure 1 Annual percent change (APC) and average annual percent change (AAPC) in age-standardized incidence rate (ASIR) at the global level and across five SDI regions, both sexes combined, 1990–2021. ASIR, age-standardized incidence rate; APC, annual percent change; AAPC, average annual percent change. *, P value <0.01.

Supplementary Figure 2 Annual percent change (APC) and average annual percent change (AAPC) in age-standardized death rate (ASDR) for the global level and five SDI regions, both sexes combined, 1990–2021. ASDR, age-standardized death rate; APC, annual percent change; AAPC, average annual percent change. *, P value <0.01.

Supplementary Figure 3 Annual percent change (APC) and average annual percent change (AAPC) in age-standardized DALYs rate for the global level and five SDI regions, both sexes combined, 1990–2021. DALYs, Disability-adjusted life year; APC, annual percent change; AAPC, average annual percent change. *, P value <0.01.

Supplementary Figure 4 Disease burden of nasopharyngeal carcinoma by socio-demographic index (SDI) across 204 countries or territories in 2021. ASIR, age-standardized incidence rate; ASDR, age-standardized death rate; DALYs, disability-adjusted life years; SDI, Socio-demographic Index.

Supplementary Figure 5 Annual percent change (APC) and average annual percent change (AAPC) in age-standardized incidence rate across five SDI regions, by sex, 1990–2021. Joinpoint regression was conducted using a sex-stratified group comparison approach, with male and female trends modelled concurrently. * P value of APC <0.01; ** P value of AAPC between female and males <0.01. APC, annual percent change; AAPC, average annual percent change

Supplementary Figure 6 Annual percent change (APC) and average annual percent change (AAPC) in age-standardized death rate across five SDI regions, by sex, 1990–2021. Joinpoint regression was conducted using a sex-stratified group comparison approach, with male and female trends modelled concurrently. * P value of APC <0.01; ** P value of AAPC between female and males <0.01. APC, annual percent change; AAPC, average annual percent change

Supplementary Figure 7 Annual percent change (APC) and average annual percent change (AAPC) in age-standardized DALYs rate across five SDI regions, by sex, 1990–2021. Joinpoint regression was conducted using a sex-stratified group comparison approach, with male and female trends modelled concurrently. * P value of APC <0.01; ** P value of AAPC between female and males <0.01. DALYs, Disability-adjusted life year; APC, annual percent change; AAPC, average annual percent change.

Supplementary Figure 8 Disease burden of nasopharyngeal carcinoma by different gender across 204 countries or territories and socio-demographic index level in 2021. ASIR, age-standardized incidence rate; ASDR, age-standardized death rate; DALYs, disability-adjusted life years; SDI, Socio-demographic Index.

Supplementary Figure 9 Number and rate for incidence, death, and disability-adjusted life years (DALYs) of nasopharyngeal carcinoma across different age groups in 2021. DALYs, disability-adjusted life years

Supplementary Figure 10 Rate ratio for male to female in incidence, death, and disability-adjusted life years (DALYs) of nasopharyngeal carcinoma across different age groups in 1990, 2000, 2010, and 2021. DALYs, disability-adjusted life years.

Supplementary Figure 11 Aging-related ratio for incidence, death, and disability-adjusted life years (DALYs) of nasopharyngeal carcinoma by gender in 204 countries or territories. DALYs, disability-adjusted life years.

Supplementary Figure 12 The temporal and predicted trends for disease burden of nasopharyngeal carcinoma by different gender from 1990 to 2050. Blue spots represent the male levels from 1990 to 2021. Red spots represent the female levels from 1990 to 2021. The dotted line represents the beginning of the prediction (from 2022). Shading indicates the upper and lower limits of the 95% UI. DALYs, disability-adjusted life years
