# Supplementary material for: Shifting burden of nasopharyngeal carcinoma: global patterns and forecasts to 2050 from the GBD 2021
Source: Front Oncol. 2026 Jan 6;15:1687320. doi: 10.3389/fonc.2025.1687320 (PMC12815733; doi:10.3389/fonc.2025.1687320)
Supplement: Supplementary Table 1 — Global burden of nasopharyngeal carcinoma and temporal trends from 1990 to 2021 across 204 countries or territories. [file Table1.docx]

**Shifting Burden of Nasopharyngeal Carcinoma:**

**Global Patterns and Forecasts to 2050 from the GBD 2021**

**Supplementary Tables**

[Table S1. Global Burden of nasopharyngeal carcinoma and temporal trends from 1990 to 2021 across 204 countries or territories 2](#_Toc24810)

[Table S2. Average annual percent change (AAPC) estimates derived from joinpoint regression using group comparison across the five SDI regions, stratified by sex 48](#_Toc9340)

[Table S3. Age-standardised incidence, mortality, and disability-adjusted life years (DALY) rates across SDI regions, 1990–2023, with annual percentage changes (APC) by sex 50](#_Toc5239)

[Table S4. Peak age group and corresponding incidence, deaths, and DALYs rates in 2021 for major regions 59](#_Toc23651)

**Table S1. Global Burden of nasopharyngeal carcinoma and temporal trends from 1990 to 2021 across 204 countries or territories**

| **GBD regions** | **Location** | **Incidence** | | | | | |
| --- | --- | --- | --- | --- | --- | --- | --- |
|  |  | **1990** | | **2021** | | **1990-2021** | |
|  |  | **Number** | **ASR (per 100,000)** | **Number** | **ASR (per 100,000)** | **EAPC (%)** | **AAPC (%)** |
| **Central Asia** | Armenia | 6 (5 - 7) | 0.195 (0.16 - 0.232) | 12 (10 - 14) | 0.289 (0.237 - 0.35) | 1.569 (1.161-1.978) | 1.363  ( 0.843 - 1.886 ) |
|  | Azerbaijan | 10 (7 - 15) | 0.181 (0.128 - 0.249) | 17 (11 - 24) | 0.152 (0.104 - 0.218) | -0.471 (-0.644to-0.297) | -0.581  ( -0.852 to -0.309 ) |
|  | Georgia | 21 (16 - 27) | 0.336 (0.254 - 0.438) | 24 (19 - 30) | 0.466 (0.364 - 0.58) | 1.381 (0.852-1.913) | 1.143  ( 0.463 - 1.827 ) |
|  | Kazakhstan | 57 (51 - 68) | 0.408 (0.362 - 0.487) | 82 (66 - 102) | 0.43 (0.343 - 0.536) | -0.031 (-0.306-0.245) | 0.213  ( -0.193 - 0.620 ) |
|  | Kyrgyzstan | 12 (9 - 16) | 0.382 (0.285 - 0.508) | 26 (18 - 36) | 0.46 (0.317 - 0.64) | 1.142 (0.903-1.38) | 0.594  ( 0.418 - 0.770 ) |
|  | Mongolia | 5 (3 - 8) | 0.418 (0.267 - 0.644) | 12 (7 - 17) | 0.411 (0.262 - 0.598) | -0.129 (-0.362-0.105) | -0.045  ( -0.397 - 0.308 ) |
|  | Tajikistan | 21 (15 - 29) | 0.65 (0.447 - 0.924) | 32 (21 - 46) | 0.436 (0.287 - 0.634) | -1.582 (-1.747to-1.417) | -1.268  ( -1.516 to -1.019 ) |
|  | Turkmenistan | 10 (9 - 12) | 0.428 (0.377 - 0.479) | 23 (17 - 30) | 0.484 (0.368 - 0.633) | 0.435 (-0.05-0.922) | 0.477  ( -0.154 - 1.113 ) |
|  | Uzbekistan | 56 (40 - 79) | 0.406 (0.285 - 0.581) | 138 (98 - 193) | 0.454 (0.321 - 0.635) | 0.282 (0.113-0.451) | 0.348  ( 0.045 - 0.653 ) |
| **Central Europe** | Albania | 10 (8 - 14) | 0.439 (0.333 - 0.584) | 16 (11 - 23) | 0.432 (0.296 - 0.618) | 0.399 (0.088-0.711) | -0.199  ( -0.638 - 0.242 ) |
|  | Bosnia and Herzegovina | 6 (5 - 7) | 0.124 (0.105 - 0.146) | 9 (7 - 12) | 0.169 (0.127 - 0.219) | 1.636 (1.264-2.009) | 1.031  ( 0.734 - 1.328 ) |
|  | Bulgaria | 34 (27 - 41) | 0.302 (0.247 - 0.366) | 58 (44 - 78) | 0.544 (0.411 - 0.72) | 1.444 (1.129-1.76) | 2.054  ( 1.432 - 2.679 ) |
|  | Croatia | 25 (21 - 30) | 0.403 (0.342 - 0.476) | 23 (18 - 28) | 0.318 (0.256 - 0.391) | -0.694 (-1.145to-0.242) | -0.728  ( -1.719 - 0.274 ) |
|  | Czechia | 68 (56 - 81) | 0.53 (0.441 - 0.631) | 60 (42 - 82) | 0.377 (0.263 - 0.517) | -1.082 (-1.374to-0.79) | -1.025  ( -1.241 to -0.808 ) |
|  | Hungary | 71 (61 - 81) | 0.531 (0.46 - 0.606) | 87 (67 - 111) | 0.563 (0.447 - 0.71) | 0.066 (-0.376-0.511) | 0.144  ( -0.222 - 0.513 ) |
|  | Montenegro | 1 (1 - 1) | 0.119 (0.101 - 0.142) | 1 (1 - 1) | 0.127 (0.099 - 0.161) | 0.246 (0.113-0.379) | 0.281  ( -0.012 - 0.574 ) |
|  | North Macedonia | 6 (5 - 7) | 0.303 (0.25 - 0.369) | 10 (8 - 14) | 0.33 (0.245 - 0.445) | 0.465 (0.116-0.815) | 0.323  ( -0.095 - 0.741 ) |
|  | Poland | 162 (153 - 172) | 0.381 (0.359 - 0.404) | 220 (197 - 243) | 0.366 (0.327 - 0.405) | -0.135 (-0.578-0.309) | -0.211  ( -0.741 - 0.321 ) |
|  | Romania | 101 (82 - 123) | 0.376 (0.306 - 0.456) | 215 (161 - 280) | 0.726 (0.539 - 0.947) | 2.176 (1.591-2.764) | 2.207  ( 1.901 - 2.513 ) |
|  | Serbia | 49 (35 - 69) | 0.431 (0.306 - 0.597) | 52 (37 - 73) | 0.376 (0.265 - 0.524) | -0.316 (-0.529to-0.103) | -0.385  ( -0.758 to -0.011 ) |
|  | Slovakia | 41 (32 - 51) | 0.713 (0.557 - 0.898) | 38 (27 - 54) | 0.457 (0.321 - 0.65) | -1.056 (-1.421to-0.689) | -1.408  ( -1.739 to -1.076 ) |
|  | Slovenia | 12 (9 - 14) | 0.484 (0.391 - 0.598) | 6 (4 - 9) | 0.18 (0.123 - 0.253) | -3.235 (-3.819to-2.647) | -3.353  ( -4.113 to -2.587 ) |
| **Eastern Europe** | Belarus | 48 (38 - 62) | 0.384 (0.308 - 0.486) | 46 (33 - 64) | 0.32 (0.232 - 0.433) | -0.972 (-1.234to-0.709) | -0.555  ( -1.165 - 0.059 ) |
|  | Estonia | 14 (10 - 18) | 0.706 (0.537 - 0.936) | 6 (4 - 8) | 0.286 (0.204 - 0.385) | -3.165 (-3.314to-3.015) | -3.007  ( -3.745 to -2.263 ) |
|  | Latvia | 25 (20 - 31) | 0.755 (0.608 - 0.93) | 9 (7 - 13) | 0.303 (0.218 - 0.411) | -3.696 (-4.044to-3.346) | -2.969  ( -4.045 to -1.882 ) |
|  | Lithuania | 30 (24 - 36) | 0.69 (0.565 - 0.829) | 13 (10 - 16) | 0.281 (0.215 - 0.361) | -2.922 (-3.242to-2.601) | -2.964  ( -3.888 to -2.032 ) |
|  | Republic of Moldova | 31 (27 - 35) | 0.689 (0.594 - 0.779) | 38 (31 - 46) | 0.691 (0.559 - 0.844) | -0.071 (-0.255-0.114) | -0.067  ( -0.693 - 0.564 ) |
|  | Russian Federation | 553 (522 - 613) | 0.313 (0.295 - 0.348) | 623 (561 - 706) | 0.29 (0.261 - 0.328) | -0.801 (-1.067to-0.533) | -0.033  ( -1.115 - 1.061 ) |
|  | Ukraine | 195 (148 - 274) | 0.291 (0.222 - 0.406) | 271 (182 - 393) | 0.414 (0.281 - 0.59) | 0.959 (0.669-1.25) | 1.150  ( 0.327 - 1.979 ) |
| **Australasia** | Australia | 243 (214 - 274) | 1.297 (1.137 - 1.463) | 284 (219 - 358) | 0.816 (0.623 - 1.037) | -1.693 (-1.883to-1.502) | -1.498  ( -1.956 to -1.038 ) |
|  | New Zealand | 30 (25 - 37) | 0.818 (0.679 - 0.995) | 33 (26 - 41) | 0.493 (0.386 - 0.614) | -1.629 (-1.886to-1.371) | -1.666  ( -2.116 to -1.214 ) |
| **High-income Asia Pacific** | Brunei Darussalam | 5 (4 - 6) | 3.457 (2.733 - 4.222) | 11 (9 - 14) | 2.668 (2.144 - 3.255) | -0.124 (-0.331-0.084) | -0.795  ( -0.943 to -0.647 ) |
|  | Japan | 497 (476 - 517) | 0.301 (0.288 - 0.313) | 980 (893 - 1049) | 0.35 (0.325 - 0.375) | 0.18 (-0.268-0.631) | 0.417  ( 0.041 - 0.795 ) |
|  | Republic of Korea | 172 (143 - 199) | 0.495 (0.409 - 0.572) | 265 (225 - 308) | 0.321 (0.273 - 0.37) | -1.187 (-1.393to-0.98) | -1.386  ( -1.546 to -1.226 ) |
|  | Singapore | 177 (157 - 200) | 6.49 (5.722 - 7.377) | 227 (176 - 286) | 2.8 (2.158 - 3.56) | -2.795 (-3.016to-2.572) | -2.671  ( -2.956 to -2.384 ) |
| **High-income North America** | Canada | 223 (194 - 250) | 0.725 (0.634 - 0.813) | 247 (192 - 308) | 0.472 (0.368 - 0.593) | -1.633 (-1.76to-1.506) | -1.306  ( -1.670 to -0.942 ) |
|  | Greenland | 5 (4 - 6) | 10.668 (9.12 - 12.276) | 3 (3 - 4) | 4.665 (3.765 - 5.85) | -2.69 (-2.825to-2.554) | -2.532  ( -2.836 to -2.227 ) |
|  | United States of America | 1948 (1886 - 2012) | 0.685 (0.665 - 0.708) | 2382 (2268 - 2491) | 0.517 (0.494 - 0.539) | -1.029 (-1.094to-0.964) | -0.929  ( -1.174 to -0.683 ) |
| **Southern Latin America** | Argentina | 124 (109 - 144) | 0.388 (0.339 - 0.449) | 109 (85 - 138) | 0.205 (0.16 - 0.261) | -1.717 (-1.832to-1.601) | -1.973  ( -2.322 to -1.623 ) |
|  | Chile | 21 (18 - 25) | 0.195 (0.164 - 0.232) | 24 (19 - 31) | 0.102 (0.079 - 0.13) | -1.638 (-1.916to-1.358) | -1.951  ( -2.529 to -1.370 ) |
|  | Uruguay | 22 (19 - 26) | 0.596 (0.513 - 0.702) | 18 (14 - 23) | 0.389 (0.3 - 0.496) | -1.258 (-1.447to-1.068) | -1.358  ( -1.851 to -0.863 ) |
| **Western Europe** | Andorra | 0 (0 - 0) | 0.38 (0.26 - 0.539) | 0 (0 - 1) | 0.27 (0.173 - 0.395) | -0.847 (-1.045to-0.65) | -1.190  ( -1.551 to -0.829 ) |
|  | Austria | 47 (40 - 54) | 0.474 (0.402 - 0.538) | 44 (34 - 54) | 0.319 (0.25 - 0.405) | -1.194 (-1.343to-1.045) | -1.284  ( -1.566 to -1.001 ) |
|  | Belgium | 80 (66 - 95) | 0.619 (0.51 - 0.741) | 85 (67 - 105) | 0.504 (0.394 - 0.622) | -0.93 (-1.184to-0.674) | -0.853  ( -1.249 to -0.454 ) |
|  | Cyprus | 2 (2 - 3) | 0.293 (0.233 - 0.361) | 4 (3 - 6) | 0.243 (0.186 - 0.311) | -0.128 (-0.322-0.067) | -0.674  ( -0.933 to -0.415 ) |
|  | Denmark | 22 (19 - 27) | 0.335 (0.289 - 0.394) | 24 (18 - 31) | 0.271 (0.205 - 0.353) | -0.74 (-0.954to-0.527) | -0.616  ( -1.109 to -0.120 ) |
|  | Finland | 15 (13 - 18) | 0.235 (0.199 - 0.276) | 13 (10 - 17) | 0.156 (0.12 - 0.201) | -1.228 (-1.451to-1.004) | -1.243  ( -1.463 to -1.022 ) |
|  | France | 978 (863 - 1125) | 1.414 (1.231 - 1.645) | 764 (593 - 990) | 0.809 (0.62 - 1.046) | -1.852 (-1.982to-1.723) | -1.777  ( -1.888 to -1.665 ) |
|  | Germany | 556 (487 - 636) | 0.514 (0.447 - 0.587) | 440 (339 - 566) | 0.311 (0.243 - 0.397) | -1.75 (-1.885to-1.614) | -1.688  ( -2.163 to -1.211 ) |
|  | Greece | 92 (84 - 101) | 0.689 (0.625 - 0.753) | 110 (97 - 125) | 0.665 (0.579 - 0.761) | -0.363 (-0.486to-0.24) | -0.146  ( -0.428 - 0.137 ) |
|  | Iceland | 1 (1 - 1) | 0.471 (0.396 - 0.56) | 2 (1 - 2) | 0.398 (0.314 - 0.523) | -0.822 (-0.966to-0.677) | -0.503  ( -0.726 to -0.279 ) |
|  | Ireland | 19 (16 - 21) | 0.493 (0.429 - 0.568) | 22 (18 - 28) | 0.341 (0.268 - 0.43) | -0.745 (-0.894to-0.597) | -1.231  ( -1.695 to -0.765 ) |
|  | Israel | 21 (17 - 26) | 0.459 (0.372 - 0.559) | 36 (28 - 46) | 0.338 (0.264 - 0.431) | -1.135 (-1.359to-0.911) | -1.025  ( -1.435 to -0.614 ) |
|  | Italy | 505 (469 - 544) | 0.672 (0.621 - 0.725) | 429 (378 - 482) | 0.45 (0.397 - 0.511) | -1.413 (-1.625to-1.2) | -1.407  ( -1.847 to -0.966 ) |
|  | Luxembourg | 4 (4 - 5) | 0.858 (0.765 - 0.972) | 5 (4 - 5) | 0.485 (0.409 - 0.58) | -1.946 (-2.095to-1.797) | -1.726  ( -1.903 to -1.549 ) |
|  | Malta | 7 (6 - 8) | 1.596 (1.35 - 1.894) | 9 (7 - 11) | 1.331 (1.014 - 1.687) | -0.585 (-0.639to-0.532) | -0.568  ( -0.954 to -0.180 ) |
|  | Monaco | 0 (0 - 0) | 0.417 (0.319 - 0.53) | 0 (0 - 0) | 0.478 (0.358 - 0.623) | 0.553 (0.393-0.713) | 0.423  ( 0.295 - 0.552 ) |
|  | Netherlands | 123 (108 - 140) | 0.688 (0.602 - 0.789) | 148 (118 - 185) | 0.575 (0.456 - 0.715) | -0.647 (-0.829to-0.466) | -0.598  ( -0.864 to -0.331 ) |
|  | Norway | 15 (14 - 16) | 0.265 (0.245 - 0.288) | 12 (10 - 14) | 0.157 (0.133 - 0.184) | -1.697 (-1.94to-1.455) | -1.657  ( -2.037 to -1.275 ) |
|  | Portugal | 87 (73 - 102) | 0.694 (0.585 - 0.813) | 93 (69 - 124) | 0.547 (0.409 - 0.727) | -0.916 (-1.067to-0.765) | -0.836  ( -1.354 to -0.315 ) |
|  | San Marino | 0 (0 - 0) | 1.095 (0.894 - 1.313) | 0 (0 - 0) | 0.635 (0.379 - 0.945) | -0.986 (-1.298to-0.673) | -1.918  ( -2.224 to -1.610 ) |
|  | Spain | 442 (392 - 496) | 0.943 (0.832 - 1.065) | 415 (330 - 531) | 0.586 (0.467 - 0.742) | -1.8 (-1.943to-1.658) | -1.620  ( -2.050 to -1.188 ) |
|  | Sweden | 38 (31 - 46) | 0.337 (0.274 - 0.41) | 34 (26 - 43) | 0.227 (0.175 - 0.291) | -1.289 (-1.576to-1.002) | -1.059  ( -2.409 - 0.311 ) |
|  | Switzerland | 61 (53 - 69) | 0.683 (0.587 - 0.77) | 32 (25 - 41) | 0.227 (0.181 - 0.286) | -4.018 (-4.771to-3.26) | -3.636  ( -4.125 to -3.144 ) |
|  | United Kingdom | 390 (378 - 400) | 0.532 (0.517 - 0.547) | 459 (440 - 477) | 0.492 (0.473 - 0.51) | -0.119 (-0.218to-0.019) | -0.308  ( -0.742 - 0.127 ) |
| **Andean Latin America** | Bolivia  (Plurinational State of) | 9 (7 - 11) | 0.246 (0.188 - 0.315) | 19 (15 - 26) | 0.2 (0.154 - 0.273) | -0.737 (-0.768to-0.706) | -0.676  ( -0.753 to -0.599 ) |
|  | Ecuador | 8 (7 - 10) | 0.14 (0.118 - 0.164) | 20 (14 - 26) | 0.118 (0.084 - 0.157) | -0.205 (-0.64-0.232) | -0.679  ( -1.147 to -0.209 ) |
|  | Peru | 18 (15 - 22) | 0.135 (0.114 - 0.163) | 41 (30 - 54) | 0.118 (0.087 - 0.156) | -0.537 (-0.703to-0.371) | -0.367  ( -1.413 - 0.690 ) |
| **Caribbean** | Antigua and Barbuda | 0 (0 - 0) | 0.314 (0.286 - 0.344) | 0 (0 - 0) | 0.375 (0.334 - 0.42) | 0.647 (0.48-0.815) | 0.641  ( 0.235 - 1.049 ) |
|  | Bahamas | 1 (1 - 1) | 0.504 (0.445 - 0.573) | 3 (2 - 3) | 0.61 (0.475 - 0.777) | 0.731 (0.627-0.835) | 0.650  ( 0.366 - 0.934 ) |
|  | Barbados | 1 (1 - 1) | 0.472 (0.423 - 0.534) | 3 (2 - 4) | 0.613 (0.478 - 0.779) | 0.87 (0.571-1.169) | 1.043  ( 0.442 - 1.647 ) |
|  | Belize | 0 (0 - 0) | 0.206 (0.188 - 0.226) | 1 (1 - 1) | 0.323 (0.275 - 0.377) | 1.404 (0.835-1.976) | 1.474  ( 1.069 - 1.881 ) |
|  | Bermuda | 0 (0 - 0) | 0.583 (0.49 - 0.69) | 1 (1 - 1) | 0.598 (0.439 - 0.825) | 0.269 (0.181-0.358) | 0.003  ( -0.241 - 0.247 ) |
|  | Cuba | 43 (38 - 49) | 0.421 (0.367 - 0.476) | 110 (87 - 140) | 0.603 (0.48 - 0.759) | 1.201 (1.087-1.316) | 1.305  ( 1.130 - 1.481 ) |
|  | Dominica | 0 (0 - 0) | 0.391 (0.347 - 0.438) | 0 (0 - 1) | 0.506 (0.414 - 0.626) | 0.97 (0.821-1.12) | 0.826  ( 0.695 - 0.957 ) |
|  | Dominican Republic | 18 (15 - 22) | 0.434 (0.358 - 0.526) | 42 (32 - 56) | 0.409 (0.307 - 0.538) | 0.094 (-0.08-0.268) | -0.150  ( -0.552 - 0.254 ) |
|  | Grenada | 0 (0 - 0) | 0.533 (0.431 - 0.664) | 1 (1 - 1) | 0.6 (0.481 - 0.744) | 0.62 (0.322-0.918) | 0.441  ( 0.123 - 0.760 ) |
|  | Guyana | 1 (1 - 1) | 0.22 (0.176 - 0.266) | 2 (1 - 3) | 0.3 (0.21 - 0.443) | 1.297 (1.035-1.561) | 1.057  ( 0.471 - 1.647 ) |
|  | Haiti | 25 (14 - 34) | 0.696 (0.391 - 0.908) | 55 (34 - 79) | 0.672 (0.42 - 0.95) | 0.049 (-0.029-0.128) | -0.040  ( -0.139 - 0.059 ) |
|  | Jamaica | 5 (4 - 7) | 0.295 (0.237 - 0.363) | 15 (10 - 21) | 0.486 (0.335 - 0.679) | 1.568 (1.084-2.054) | 2.325  ( 1.055 - 3.611 ) |
|  | Puerto Rico | 16 (14 - 19) | 0.464 (0.395 - 0.541) | 23 (18 - 29) | 0.423 (0.328 - 0.536) | -0.251 (-0.528-0.026) | -0.317  ( -0.683 - 0.050 ) |
|  | Saint Kitts and Nevis | 0 (0 - 0) | 0.693 (0.605 - 0.797) | 1 (0 - 1) | 0.706 (0.574 - 0.861) | 0.336 (0.194-0.477) | 0.036  ( -0.354 - 0.427 ) |
|  | Saint Lucia | 1 (1 - 1) | 0.713 (0.64 - 0.783) | 2 (1 - 2) | 0.751 (0.597 - 0.93) | -0.046 (-0.155-0.064) | 0.176  ( -0.006 - 0.359 ) |
|  | Saint Vincent and the Grenadines | 0 (0 - 0) | 0.507 (0.44 - 0.591) | 1 (1 - 1) | 0.635 (0.531 - 0.754) | 0.654 (0.546-0.762) | 0.654  ( 0.244 - 1.065 ) |
|  | Suriname | 2 (2 - 2) | 0.624 (0.544 - 0.706) | 5 (3 - 6) | 0.705 (0.538 - 0.885) | 0.578 (0.345-0.812) | 0.510  ( 0.004 - 1.020 ) |
|  | Trinidad and Tobago | 3 (3 - 3) | 0.342 (0.314 - 0.373) | 7 (6 - 10) | 0.405 (0.297 - 0.526) | 0.431 (0.256-0.607) | 0.599  ( 0.365 - 0.832 ) |
|  | United States Virgin Islands | 1 (1 - 1) | 0.688 (0.571 - 0.816) | 1 (0 - 1) | 0.439 (0.344 - 0.564) | -1.098 (-1.294to-0.901) | -1.423  ( -1.757 to -1.089 ) |
| **Central Latin America** | Colombia | 65 (57 - 74) | 0.308 (0.271 - 0.353) | 105 (78 - 139) | 0.192 (0.143 - 0.255) | -2.108 (-2.312to-1.904) | -1.625  ( -1.869 to -1.380 ) |
|  | Costa Rica | 11 (9 - 12) | 0.535 (0.465 - 0.609) | 22 (18 - 28) | 0.408 (0.332 - 0.506) | -1.448 (-1.672to-1.224) | -0.939  ( -1.254 to -0.623 ) |
|  | El Salvador | 7 (6 - 8) | 0.206 (0.186 - 0.228) | 14 (11 - 17) | 0.221 (0.174 - 0.273) | 0.069 (-0.066-0.204) | 0.220  ( -0.461 - 0.906 ) |
|  | Guatemala | 12 (11 - 12) | 0.28 (0.258 - 0.302) | 24 (20 - 29) | 0.201 (0.167 - 0.237) | -1.331 (-1.617to-1.044) | -1.204  ( -1.797 to -0.607 ) |
|  | Honduras | 5 (4 - 6) | 0.211 (0.171 - 0.253) | 16 (12 - 20) | 0.229 (0.18 - 0.292) | 0.341 (0.253-0.428) | 0.307  ( -0.022 - 0.638 ) |
|  | Mexico | 85 (82 - 89) | 0.178 (0.172 - 0.186) | 188 (161 - 214) | 0.144 (0.123 - 0.164) | -0.894 (-0.986to-0.802) | -0.751  ( -1.101 to -0.399 ) |
|  | Nicaragua | 4 (3 - 4) | 0.193 (0.163 - 0.225) | 10 (8 - 13) | 0.19 (0.151 - 0.241) | 0.026 (-0.256-0.308) | 0.078  ( -0.105 - 0.262 ) |
|  | Panama | 5 (4 - 5) | 0.305 (0.275 - 0.336) | 13 (10 - 16) | 0.299 (0.231 - 0.37) | -0.258 (-0.473to-0.042) | -0.080  ( -0.343 - 0.184 ) |
|  | Venezuela  (Bolivarian Republic of) | 26 (24 - 29) | 0.241 (0.216 - 0.267) | 89 (65 - 121) | 0.298 (0.217 - 0.404) | 0.351 (0.083-0.618) | 0.686  ( 0.333 - 1.041 ) |
| **Tropical Latin America** | Brazil | 217 (203 - 235) | 0.201 (0.187 - 0.217) | 602 (553 - 656) | 0.238 (0.219 - 0.26) | 0.233 (-0.198-0.667) | 0.525  ( 0.142 - 0.909 ) |
|  | Paraguay | 3 (2 - 3) | 0.103 (0.084 - 0.127) | 11 (8 - 15) | 0.178 (0.131 - 0.236) | 1.742 (1.372-2.114) | 1.747  ( 1.373 - 2.122 ) |
| **North Africa and Middle East** | Afghanistan | 61 (27 - 87) | 0.816 (0.374 - 1.157) | 79 (39 - 114) | 0.57 (0.277 - 0.796) | -1.422 (-1.536to-1.308) | -1.155  ( -1.227 to -1.083 ) |
|  | Algeria | 392 (322 - 475) | 2.631 (2.142 - 3.22) | 867 (661 - 1098) | 2.141 (1.644 - 2.702) | -0.606 (-0.683to-0.529) | -0.672  ( -0.757 to -0.588 ) |
|  | Bahrain | 2 (2 - 2) | 0.872 (0.719 - 1.052) | 6 (4 - 9) | 0.502 (0.362 - 0.684) | -2.281 (-2.561to-2.001) | -1.702  ( -1.998 to -1.404 ) |
|  | Egypt | 34 (30 - 40) | 0.107 (0.094 - 0.124) | 55 (44 - 69) | 0.074 (0.06 - 0.092) | -1.218 (-1.479to-0.957) | -1.187  ( -1.483 to -0.890 ) |
|  | Iran  (Islamic Republic of) | 67 (59 - 76) | 0.216 (0.19 - 0.243) | 136 (123 - 151) | 0.162 (0.146 - 0.181) | -0.963 (-1.099to-0.827) | -0.918  ( -1.163 to -0.673 ) |
|  | Iraq | 51 (40 - 64) | 0.516 (0.404 - 0.647) | 108 (80 - 143) | 0.348 (0.258 - 0.456) | -1.761 (-1.998to-1.524) | -1.290  ( -1.559 to -1.021 ) |
|  | Jordan | 17 (14 - 21) | 0.939 (0.757 - 1.143) | 54 (39 - 73) | 0.544 (0.396 - 0.727) | -2.185 (-2.435to-1.934) | -1.723  ( -1.943 to -1.503 ) |
|  | Kuwait | 8 (7 - 10) | 0.814 (0.68 - 0.976) | 13 (10 - 16) | 0.282 (0.216 - 0.363) | -2.799 (-3.218to-2.379) | -3.530  ( -5.005 to -2.032 ) |
|  | Lebanon | 13 (9 - 18) | 0.543 (0.379 - 0.744) | 23 (18 - 30) | 0.387 (0.304 - 0.496) | -0.916 (-1.029to-0.802) | -1.069  ( -1.259 to -0.879 ) |
|  | Libya | 66 (49 - 84) | 2.775 (2.063 - 3.558) | 190 (138 - 256) | 2.783 (2.055 - 3.669) | 0.205 (0.111-0.299) | 0.042  ( -0.166 - 0.251 ) |
|  | Morocco | 230 (185 - 280) | 1.404 (1.126 - 1.726) | 462 (327 - 612) | 1.239 (0.88 - 1.624) | -0.365 (-0.442to-0.289) | -0.381  ( -0.464 to -0.299 ) |
|  | Oman | 5 (4 - 7) | 0.532 (0.383 - 0.72) | 11 (7 - 15) | 0.321 (0.236 - 0.416) | -1.57 (-1.706to-1.434) | -1.632  ( -2.074 to -1.187 ) |
|  | Palestine | 3 (3 - 4) | 0.343 (0.26 - 0.442) | 8 (6 - 9) | 0.242 (0.201 - 0.29) | -1.232 (-1.402to-1.063) | -1.138  ( -1.297 to -0.980 ) |
|  | Qatar | 1 (1 - 1) | 0.493 (0.381 - 0.615) | 7 (4 - 10) | 0.316 (0.218 - 0.463) | -1.473 (-1.956to-0.986) | -1.440  ( -1.982 to -0.895 ) |
|  | Saudi Arabia | 122 (90 - 158) | 1.498 (1.107 - 1.916) | 380 (283 - 508) | 1.092 (0.848 - 1.392) | -1.282 (-1.457to-1.106) | -0.997  ( -1.183 to -0.811 ) |
|  | Sudan | 57 (36 - 85) | 0.521 (0.33 - 0.762) | 76 (52 - 106) | 0.298 (0.212 - 0.415) | -2.025 (-2.134to-1.915) | -1.795  ( -1.840 to -1.751 ) |
|  | Syrian Arab Republic | 11 (9 - 14) | 0.167 (0.129 - 0.206) | 16 (12 - 21) | 0.115 (0.086 - 0.152) | -1.734 (-2.011to-1.456) | -1.266  ( -1.501 to -1.030 ) |
|  | Tunisia | 156 (124 - 194) | 2.69 (2.146 - 3.35) | 315 (225 - 437) | 2.316 (1.655 - 3.202) | -0.704 (-0.786to-0.621) | -0.481  ( -0.591 to -0.370 ) |
|  | Türkiye | 367 (298 - 456) | 0.904 (0.733 - 1.122) | 532 (424 - 648) | 0.564 (0.451 - 0.684) | -1.688 (-1.837to-1.538) | -1.532  ( -1.831 to -1.231 ) |
|  | United Arab Emirates | 7 (5 - 10) | 0.842 (0.567 - 1.23) | 35 (27 - 45) | 0.536 (0.416 - 0.653) | -0.46 (-0.794to-0.125) | -1.349  ( -1.823 to -0.874 ) |
|  | Yemen | 30 (20 - 42) | 0.509 (0.345 - 0.714) | 60 (41 - 82) | 0.344 (0.238 - 0.469) | -1.496 (-1.604to-1.387) | -1.226  ( -1.384 to -1.068 ) |
| **South Asia** | Bangladesh | 971 (698 - 1214) | 1.661 (1.204 - 2.07) | 1473 (947 - 2222) | 0.977 (0.631 - 1.464) | -1.741 (-1.84to-1.641) | -1.647  ( -1.899 to -1.394 ) |
|  | Bhutan | 5 (3 - 7) | 1.434 (0.94 - 2.045) | 6 (4 - 10) | 0.918 (0.608 - 1.427) | -1.609 (-1.762to-1.457) | -1.444  ( -1.568 to -1.319 ) |
|  | India | 7907 (6707 - 9165) | 1.362 (1.153 - 1.581) | 13166 (11573 - 14842) | 0.997 (0.877 - 1.121) | -1.213 (-1.467to-0.959) | -1.001  ( -1.150 to -0.852 ) |
|  | Nepal | 156 (113 - 212) | 1.331 (0.969 - 1.806) | 260 (188 - 354) | 1.005 (0.731 - 1.362) | -0.796 (-1.089to-0.503) | -0.895  ( -0.984 to -0.805 ) |
|  | Pakistan | 904 (760 - 1093) | 1.36 (1.138 - 1.646) | 2125 (1672 - 2676) | 1.326 (1.042 - 1.671) | -0.355 (-0.512to-0.198) | -0.055  ( -0.127 - 0.017 ) |
| **East Asia** | China | 44864 (38023 - 51827) | 4.639 (3.935 - 5.358) | 65934 (53272 - 81430) | 3.423 (2.772 - 4.232) | -1.499 (-1.909to-1.086) | -0.978  ( -1.168 to -0.787 ) |
|  | Democratic People's Republic of Korea | 409 (287 - 542) | 2.167 (1.533 - 2.859) | 617 (458 - 818) | 1.832 (1.361 - 2.405) | -0.578 (-0.63to-0.526) | -0.539  ( -0.584 to -0.494 ) |
|  | Taiwan  (Province of China) | 1329 (1193 - 1465) | 7.077 (6.396 - 7.783) | 1488 (1168 - 1925) | 4.27 (3.326 - 5.512) | -1.935 (-2.071to-1.798) | -1.667  ( -1.816 to -1.517 ) |
| **Oceania** | American Samoa | 0 (0 - 0) | 1.43 (1.151 - 1.744) | 1 (1 - 1) | 1.572 (1.266 - 1.968) | 0.746 (0.565-0.928) | 0.301  ( -0.146 - 0.749 ) |
|  | Cook Islands | 0 (0 - 0) | 0.228 (0.18 - 0.282) | 0 (0 - 0) | 0.214 (0.162 - 0.276) | -0.004 (-0.18-0.172) | -0.208  ( -0.574 - 0.159 ) |
|  | Fiji | 2 (1 - 2) | 0.382 (0.307 - 0.473) | 3 (2 - 4) | 0.318 (0.236 - 0.431) | -0.635 (-0.847to-0.422) | -0.635  ( -0.911 to -0.358 ) |
|  | Guam | 2 (2 - 3) | 2.633 (2.356 - 2.915) | 3 (3 - 4) | 1.697 (1.462 - 1.969) | -0.98 (-1.229to-0.73) | -1.461  ( -2.387 to -0.527 ) |
|  | Kiribati | 0 (0 - 0) | 0.846 (0.649 - 1.054) | 1 (1 - 1) | 0.801 (0.588 - 1.101) | -0.099 (-0.151to-0.047) | -0.175  ( -0.220 to -0.129 ) |
|  | Marshall Islands | 0 (0 - 0) | 1.232 (0.913 - 1.565) | 0 (0 - 1) | 1.1 (0.793 - 1.453) | -0.383 (-0.438to-0.328) | -0.368  ( -0.419 to -0.316 ) |
|  | Micronesia  (Federated States of) | 1 (1 - 1) | 1.385 (1.022 - 1.778) | 1 (1 - 1) | 1.046 (0.783 - 1.399) | -0.961 (-1.073to-0.849) | -0.908  ( -0.966 to -0.850 ) |
|  | Nauru | 0 (0 - 0) | 1.541 (1.034 - 2.123) | 0 (0 - 0) | 1.222 (0.768 - 1.805) | -0.812 (-0.93to-0.693) | -0.753  ( -0.800 to -0.707 ) |
|  | Niue | 0 (0 - 0) | 1.037 (0.773 - 1.357) | 0 (0 - 0) | 0.885 (0.665 - 1.141) | -0.828 (-0.941to-0.715) | -0.499  ( -0.579 to -0.420 ) |
|  | Northern Mariana Islands | 0 (0 - 1) | 1.782 (1.414 - 2.273) | 1 (1 - 1) | 1.617 (1.356 - 1.9) | -0.13 (-0.293-0.033) | -0.366  ( -0.954 - 0.226 ) |
|  | Palau | 0 (0 - 0) | 0.06 (0.04 - 0.09) | 0 (0 - 0) | 0.046 (0.033 - 0.063) | -0.618 (-0.768to-0.467) | -0.859  ( -0.987 to -0.731 ) |
|  | Papua New Guinea | 21 (12 - 32) | 0.951 (0.577 - 1.422) | 50 (30 - 79) | 0.788 (0.486 - 1.241) | -0.585 (-0.69to-0.48) | -0.577  ( -0.636 to -0.518 ) |
|  | Samoa | 2 (1 - 2) | 1.806 (1.442 - 2.271) | 3 (2 - 4) | 1.701 (1.304 - 2.221) | -0.218 (-0.311to-0.125) | -0.190  ( -0.215 to -0.164 ) |
|  | Solomon Islands | 2 (1 - 3) | 1.185 (0.679 - 1.7) | 4 (3 - 6) | 1.036 (0.742 - 1.406) | -0.38 (-0.513to-0.247) | -0.456  ( -0.594 to -0.317 ) |
|  | Tokelau | 0 (0 - 0) | 1.118 (0.803 - 1.5) | 0 (0 - 0) | 0.865 (0.613 - 1.19) | -1.027 (-1.131to-0.924) | -0.813  ( -0.964 to -0.661 ) |
|  | Tonga | 1 (0 - 1) | 0.828 (0.597 - 1.131) | 1 (0 - 1) | 0.724 (0.498 - 1.028) | -0.444 (-0.499to-0.389) | -0.432  ( -0.542 to -0.322 ) |
|  | Tuvalu | 0 (0 - 0) | 1.189 (0.871 - 1.506) | 0 (0 - 0) | 0.939 (0.764 - 1.176) | -0.749 (-0.816to-0.681) | -0.757  ( -0.808 to -0.706 ) |
|  | Vanuatu | 1 (1 - 1) | 1.05 (0.757 - 1.42) | 2 (1 - 2) | 0.892 (0.678 - 1.143) | -0.639 (-0.758to-0.52) | -0.544  ( -0.743 to -0.345 ) |
| **Southeast Asia** | Cambodia | 114 (87 - 144) | 2.137 (1.626 - 2.678) | 286 (210 - 382) | 2.051 (1.513 - 2.717) | -0.257 (-0.378to-0.136) | -0.094  ( -0.222 - 0.034 ) |
|  | Indonesia | 1792 (1417 - 2233) | 1.495 (1.187 - 1.853) | 3635 (2607 - 4882) | 1.342 (0.981 - 1.767) | -0.432 (-0.496to-0.367) | -0.338  ( -0.410 to -0.265 ) |
|  | Lao People's Democratic Republic | 55 (35 - 75) | 2.288 (1.499 - 3.081) | 87 (62 - 117) | 1.591 (1.148 - 2.12) | -1.331 (-1.415to-1.247) | -1.170  ( -1.213 to -1.127 ) |
|  | Malaysia | 819 (696 - 956) | 7.087 (6.024 - 8.281) | 1935 (1650 - 2290) | 6.091 (5.213 - 7.189) | -0.874 (-1.216to-0.531) | -0.455  ( -0.762 to -0.147 ) |
|  | Maldives | 1 (0 - 1) | 0.508 (0.34 - 0.654) | 1 (1 - 1) | 0.25 (0.197 - 0.313) | -2.606 (-2.714to-2.498) | -2.391  ( -2.628 to -2.155 ) |
|  | Mauritius | 7 (6 - 8) | 0.828 (0.754 - 0.916) | 15 (13 - 17) | 0.869 (0.75 - 0.974) | -0.154 (-0.384-0.076) | 0.171  ( -0.633 - 0.981 ) |
|  | Myanmar | 531 (381 - 701) | 1.979 (1.445 - 2.596) | 700 (527 - 905) | 1.328 (1.015 - 1.693) | -1.579 (-1.685to-1.473) | -1.297  ( -1.382 to -1.212 ) |
|  | Philippines | 620 (554 - 707) | 1.673 (1.461 - 1.934) | 1549 (1289 - 1841) | 1.638 (1.368 - 1.943) | -0.066 (-0.134-0.003) | -0.057  ( -0.242 - 0.128 ) |
|  | Seychelles | 1 (1 - 1) | 1.677 (1.416 - 1.961) | 2 (2 - 2) | 1.648 (1.366 - 1.982) | -0.022 (-0.313-0.27) | 0.000  ( -0.199 - 0.200 ) |
|  | Sri Lanka | 133 (111 - 159) | 1.08 (0.899 - 1.282) | 216 (140 - 305) | 0.818 (0.535 - 1.153) | -1.015 (-1.253to-0.777) | -0.726  ( -1.143 to -0.307 ) |
|  | Thailand | 821 (693 - 970) | 1.904 (1.611 - 2.226) | 1773 (1364 - 2250) | 1.869 (1.443 - 2.358) | -0.537 (-0.729to-0.345) | -0.033  ( -0.197 - 0.131 ) |
|  | Timor-Leste | 5 (4 - 8) | 1.462 (0.98 - 2.152) | 12 (9 - 17) | 1.343 (0.971 - 1.867) | -0.272 (-0.47to-0.073) | -0.303  ( -0.467 to -0.138 ) |
|  | Viet Nam | 1301 (989 - 1677) | 2.933 (2.216 - 3.806) | 3431 (2539 - 4457) | 3.123 (2.344 - 4.018) | 0.271 (0.155-0.386) | 0.210  ( 0.140 - 0.279 ) |
| **Central Sub-Saharan Africa** | Angola | 21 (15 - 28) | 0.426 (0.308 - 0.58) | 57 (40 - 79) | 0.38 (0.269 - 0.519) | -0.36 (-0.418to-0.302) | -0.388  ( -0.665 to -0.111 ) |
|  | Central African Republic | 7 (5 - 10) | 0.52 (0.389 - 0.675) | 13 (9 - 18) | 0.437 (0.305 - 0.634) | -0.675 (-0.739to-0.61) | -0.530  ( -0.604 to -0.455 ) |
|  | Congo | 6 (5 - 8) | 0.514 (0.4 - 0.652) | 14 (10 - 19) | 0.413 (0.305 - 0.542) | -0.911 (-1.046to-0.776) | -0.695  ( -0.960 to -0.430 ) |
|  | Democratic Republic of the Congo | 70 (50 - 96) | 0.378 (0.272 - 0.526) | 161 (111 - 241) | 0.353 (0.245 - 0.524) | -0.205 (-0.311to-0.098) | -0.247  ( -0.366 to -0.127 ) |
|  | Equatorial Guinea | 1 (1 - 1) | 0.459 (0.332 - 0.621) | 2 (1 - 4) | 0.327 (0.205 - 0.499) | -1.301 (-1.56to-1.041) | -1.024  ( -1.412 to -0.635 ) |
|  | Gabon | 3 (2 - 4) | 0.489 (0.396 - 0.595) | 5 (3 - 6) | 0.39 (0.286 - 0.515) | -0.884 (-0.984to-0.785) | -0.713  ( -0.857 to -0.569 ) |
| **Eastern Sub-Saharan Africa** | Burundi | 56 (40 - 74) | 1.964 (1.424 - 2.598) | 93 (61 - 136) | 1.385 (0.931 - 2.022) | -1.483 (-1.646to-1.319) | -1.139  ( -1.320 to -0.958 ) |
|  | Comoros | 4 (2 - 5) | 1.428 (0.903 - 2.087) | 7 (5 - 11) | 1.228 (0.783 - 1.839) | -0.746 (-0.932to-0.56) | -0.436  ( -0.780 to -0.090 ) |
|  | Djibouti | 3 (2 - 4) | 1.409 (0.876 - 2.146) | 11 (6 - 19) | 1.31 (0.76 - 2.091) | -0.34 (-0.463to-0.216) | -0.231  ( -0.321 to -0.141 ) |
|  | Eritrea | 30 (24 - 37) | 1.768 (1.42 - 2.213) | 61 (43 - 86) | 1.55 (1.117 - 2.152) | -0.459 (-0.494to-0.424) | -0.444  ( -0.591 to -0.296 ) |
|  | Ethiopia | 504 (332 - 646) | 1.955 (1.3 - 2.485) | 738 (503 - 1131) | 1.279 (0.872 - 1.932) | -1.686 (-1.855to-1.518) | -1.356  ( -1.420 to -1.292 ) |
|  | Kenya | 179 (128 - 250) | 1.685 (1.191 - 2.374) | 569 (417 - 761) | 1.879 (1.393 - 2.51) | 0.613 (0.447-0.778) | 0.341  ( 0.248 - 0.435 ) |
|  | Madagascar | 85 (65 - 109) | 1.335 (1.002 - 1.744) | 172 (113 - 254) | 1.06 (0.703 - 1.549) | -0.767 (-0.872to-0.663) | -0.742  ( -0.813 to -0.672 ) |
|  | Malawi | 17 (13 - 21) | 0.333 (0.257 - 0.419) | 33 (23 - 46) | 0.317 (0.234 - 0.434) | -0.384 (-0.505to-0.263) | -0.114  ( -0.254 - 0.026 ) |
|  | Mozambique | 6 (4 - 8) | 0.089 (0.067 - 0.113) | 14 (10 - 18) | 0.102 (0.076 - 0.13) | 0.848 (0.715-0.98) | 0.479  ( 0.395 - 0.563 ) |
|  | Rwanda | 82 (63 - 101) | 2.244 (1.725 - 2.777) | 114 (77 - 164) | 1.385 (0.962 - 1.969) | -2.327 (-2.601to-2.052) | -1.529  ( -1.673 to -1.385 ) |
|  | Somalia | 58 (39 - 82) | 1.639 (1.131 - 2.301) | 134 (88 - 212) | 1.483 (0.992 - 2.296) | -0.341 (-0.39to-0.291) | -0.318  ( -0.377 to -0.258 ) |
|  | South Sudan | 40 (27 - 59) | 1.339 (0.883 - 1.985) | 68 (43 - 107) | 1.277 (0.824 - 1.984) | -0.307 (-0.51to-0.103) | -0.125  ( -0.238 to -0.012 ) |
|  | Uganda | 238 (179 - 313) | 2.973 (2.253 - 3.904) | 560 (404 - 773) | 2.685 (1.961 - 3.667) | -1.011 (-1.295to-0.727) | -0.351  ( -0.579 to -0.123 ) |
|  | United Republic of Tanzania | 212 (155 - 278) | 1.58 (1.154 - 2.062) | 422 (285 - 604) | 1.267 (0.875 - 1.79) | -0.827 (-0.875to-0.779) | -0.717  ( -0.821 to -0.612 ) |
|  | Zambia | 59 (47 - 75) | 1.578 (1.253 - 1.989) | 222 (90 - 391) | 2.174 (0.914 - 3.753) | 1.234 (0.956-1.513) | 1.103  ( 0.958 - 1.248 ) |
| **Southern Sub-Saharan Africa** | Botswana | 4 (3 - 6) | 0.686 (0.486 - 0.957) | 10 (6 - 14) | 0.555 (0.354 - 0.798) | -0.808 (-1.033to-0.583) | -0.722  ( -1.125 to -0.317 ) |
|  | Eswatini | 2 (2 - 3) | 0.696 (0.526 - 0.887) | 5 (4 - 7) | 0.787 (0.541 - 1.077) | 0.594 (0.144-1.046) | 0.394  ( 0.218 - 0.570 ) |
|  | Lesotho | 5 (4 - 7) | 0.546 (0.4 - 0.775) | 11 (8 - 15) | 0.881 (0.64 - 1.195) | 2.105 (1.718-2.493) | 1.616  ( 1.367 - 1.866 ) |
|  | Namibia | 5 (4 - 6) | 0.596 (0.452 - 0.772) | 9 (7 - 13) | 0.543 (0.384 - 0.715) | -0.659 (-1to-0.317) | -0.245  ( -0.442 to -0.048 ) |
|  | South Africa | 128 (108 - 151) | 0.545 (0.453 - 0.648) | 250 (221 - 276) | 0.498 (0.442 - 0.549) | -0.47 (-0.693to-0.247) | -0.365  ( -0.821 - 0.092 ) |
|  | Zimbabwe | 26 (21 - 33) | 0.538 (0.435 - 0.674) | 72 (53 - 93) | 0.773 (0.585 - 0.995) | 1.655 (1.198-2.114) | 1.205  ( 0.701 - 1.711 ) |
| **Western Sub-Saharan Africa** | Benin | 3 (2 - 4) | 0.125 (0.096 - 0.158) | 10 (6 - 14) | 0.13 (0.084 - 0.188) | -0.005 (-0.13-0.119) | 0.108  ( -0.164 - 0.381 ) |
|  | Burkina Faso | 7 (5 - 10) | 0.142 (0.105 - 0.184) | 20 (14 - 27) | 0.157 (0.108 - 0.214) | 0.275 (0.137-0.414) | 0.333  ( 0.139 - 0.528 ) |
|  | Cabo Verde | 0 (0 - 0) | 0.034 (0.027 - 0.042) | 1 (1 - 2) | 0.228 (0.171 - 0.304) | 5.071 (3.547-6.618) | 6.339  ( 6.058 - 6.621 ) |
|  | Cameroon | 9 (7 - 11) | 0.148 (0.115 - 0.188) | 32 (21 - 45) | 0.166 (0.11 - 0.231) | 0.291 (0.157-0.426) | 0.360  ( 0.281 - 0.439 ) |
|  | Chad | 4 (3 - 5) | 0.11 (0.081 - 0.146) | 14 (9 - 19) | 0.157 (0.108 - 0.221) | 1.16 (0.969-1.351) | 1.200  ( 1.034 - 1.367 ) |
|  | Côte d'Ivoire | 19 (14 - 25) | 0.335 (0.246 - 0.436) | 53 (34 - 76) | 0.324 (0.215 - 0.462) | -0.235 (-0.361to-0.109) | -0.109  ( -0.316 - 0.098 ) |
|  | Gambia | 1 (1 - 1) | 0.157 (0.117 - 0.2) | 2 (2 - 3) | 0.179 (0.131 - 0.229) | 0.172 (-0.008-0.352) | 0.405  ( -0.683 - 1.506 ) |
|  | Ghana | 7 (5 - 10) | 0.08 (0.056 - 0.109) | 3 (2 - 4) | 0.012 (0.008 - 0.017) | -8.644 (-10.239to-7.021) | -5.802  ( -6.337 to -5.264 ) |
|  | Guinea | 10 (8 - 13) | 0.275 (0.214 - 0.343) | 21 (15 - 30) | 0.295 (0.215 - 0.406) | 0.342 (0.264-0.42) | 0.234  ( 0.124 - 0.344 ) |
|  | Guinea-Bissau | 1 (1 - 1) | 0.192 (0.136 - 0.264) | 3 (2 - 3) | 0.211 (0.155 - 0.281) | 0.353 (0.28-0.425) | 0.336  ( 0.222 - 0.451 ) |
|  | Liberia | 2 (1 - 2) | 0.13 (0.091 - 0.179) | 5 (3 - 8) | 0.149 (0.095 - 0.227) | 0.36 (0.21-0.51) | 0.442  ( 0.063 - 0.822 ) |
|  | Mali | 8 (7 - 11) | 0.171 (0.14 - 0.21) | 18 (13 - 25) | 0.153 (0.108 - 0.213) | -0.269 (-0.431to-0.107) | -0.348  ( -0.441 to -0.254 ) |
|  | Mauritania | 2 (1 - 2) | 0.126 (0.093 - 0.171) | 3 (2 - 5) | 0.117 (0.068 - 0.188) | -0.416 (-0.501to-0.331) | -0.252  ( -0.392 to -0.112 ) |
|  | Niger | 5 (3 - 6) | 0.116 (0.078 - 0.163) | 14 (8 - 23) | 0.115 (0.066 - 0.191) | -0.058 (-0.156-0.04) | 0.010  ( -0.112 - 0.132 ) |
|  | Nigeria | 558 (427 - 703) | 1.075 (0.826 - 1.345) | 1173 (782 - 1608) | 0.959 (0.663 - 1.288) | -0.441 (-0.55to-0.331) | -0.395  ( -0.482 to -0.308 ) |
|  | Sao Tome and Principe | 0 (0 - 0) | 0.023 (0.017 - 0.031) | 0 (0 - 0) | 0.024 (0.017 - 0.034) | -0.187 (-0.397-0.024) | 0.037  ( -0.141 - 0.215 ) |
|  | Senegal | 5 (4 - 7) | 0.128 (0.091 - 0.176) | 15 (9 - 22) | 0.143 (0.087 - 0.215) | 0.388 (0.295-0.48) | 0.377  ( 0.283 - 0.471 ) |
|  | Sierra Leone | 3 (2 - 4) | 0.125 (0.091 - 0.164) | 8 (5 - 11) | 0.145 (0.096 - 0.203) | 0.627 (0.569-0.685) | 0.513  ( 0.276 - 0.751 ) |
|  | Togo | 2 (2 - 3) | 0.135 (0.103 - 0.173) | 10 (7 - 14) | 0.165 (0.115 - 0.231) | 0.623 (0.536-0.711) | 0.664  ( 0.547 - 0.781 ) |

*(Table continues on the next page)*

**Table S1 Global Burden of nasopharyngeal carcinoma and temporal trends from 1990 to 2021 across 204 countries or territories.** *(continued)*

| **GBD regions** | **Location** | **Deaths** | | | | | |
| --- | --- | --- | --- | --- | --- | --- | --- |
|  |  | **1990** | | **2021** | | **1990-2021** | |
|  |  | **Number** | **ASR (per 100,000)** | **Number** | **ASR (per 100,000)** | **EAPC (%)** | **AAPC (%)** |
| **Central Asia** | Armenia | 6 (5 - 7) | 0.191 (0.159 - 0.227) | 11 (9 - 13) | 0.271 (0.22 - 0.326) | 1.396 (1.009-1.784) | 1.201  ( 0.734 - 1.670 ) |
|  | Azerbaijan | 10 (7 - 14) | 0.181 (0.127 - 0.248) | 16 (11 - 22) | 0.145 (0.099 - 0.207) | -0.596 (-0.78to-0.411) | -0.720  ( -0.946 to -0.493 ) |
|  | Georgia | 20 (15 - 25) | 0.32 (0.243 - 0.411) | 24 (18 - 30) | 0.44 (0.342 - 0.55) | 1.416 (0.882-1.954) | 1.129  ( 0.424 - 1.839 ) |
|  | Kazakhstan | 54 (48 - 64) | 0.399 (0.353 - 0.472) | 74 (59 - 92) | 0.395 (0.315 - 0.492) | -0.282 (-0.535to-0.028) | 0.061  ( -0.278 - 0.401 ) |
|  | Kyrgyzstan | 12 (9 - 16) | 0.372 (0.276 - 0.5) | 24 (16 - 32) | 0.433 (0.299 - 0.597) | 1.036 (0.808-1.266) | 0.482  ( 0.135 - 0.830 ) |
|  | Mongolia | 5 (3 - 8) | 0.423 (0.273 - 0.653) | 11 (7 - 16) | 0.401 (0.257 - 0.58) | -0.285 (-0.515to-0.054) | -0.182  ( -0.525 - 0.161 ) |
|  | Tajikistan | 21 (14 - 29) | 0.664 (0.453 - 0.957) | 30 (20 - 44) | 0.436 (0.286 - 0.65) | -1.638 (-1.826to-1.448) | -1.339  ( -1.617 to -1.061 ) |
|  | Turkmenistan | 10 (9 - 11) | 0.425 (0.375 - 0.476) | 21 (16 - 28) | 0.458 (0.354 - 0.605) | 0.267 (-0.211-0.747) | 0.312  ( -0.287 - 0.914 ) |
|  | Uzbekistan | 53 (38 - 76) | 0.401 (0.277 - 0.572) | 127 (89 - 178) | 0.436 (0.308 - 0.611) | 0.2 (0.036-0.365) | 0.262  ( -0.049 - 0.573 ) |
| **Central Europe** | Albania | 10 (8 - 13) | 0.441 (0.336 - 0.582) | 15 (11 - 22) | 0.382 (0.265 - 0.541) | -0.121 (-0.376-0.134) | -0.588  ( -0.958 to -0.217 ) |
|  | Bosnia and Herzegovina | 5 (5 - 6) | 0.121 (0.103 - 0.142) | 9 (7 - 11) | 0.152 (0.115 - 0.196) | 1.285 (0.948-1.625) | 0.762  ( 0.414 - 1.112 ) |
|  | Bulgaria | 29 (23 - 35) | 0.25 (0.204 - 0.308) | 46 (35 - 61) | 0.382 (0.288 - 0.509) | 0.931 (0.591-1.272) | 1.480  ( 0.949 - 2.013 ) |
|  | Croatia | 23 (20 - 28) | 0.376 (0.321 - 0.443) | 21 (17 - 26) | 0.27 (0.22 - 0.333) | -1.025 (-1.472to-0.576) | -1.043  ( -2.043 to -0.033 ) |
|  | Czechia | 60 (50 - 72) | 0.459 (0.381 - 0.55) | 41 (29 - 56) | 0.22 (0.156 - 0.3) | -2.509 (-2.675to-2.343) | -2.330  ( -2.550 to -2.109 ) |
|  | Hungary | 67 (58 - 77) | 0.496 (0.428 - 0.568) | 78 (60 - 101) | 0.473 (0.367 - 0.606) | -0.199 (-0.605-0.208) | -0.171  ( -0.734 - 0.396 ) |
|  | Montenegro | 1 (1 - 1) | 0.105 (0.088 - 0.126) | 1 (1 - 1) | 0.105 (0.083 - 0.13) | -0.042 (-0.19-0.105) | 0.067  ( -0.173 - 0.309 ) |
|  | North Macedonia | 6 (5 - 7) | 0.297 (0.245 - 0.364) | 10 (7 - 13) | 0.304 (0.228 - 0.409) | 0.197 (-0.148-0.543) | 0.099  ( -0.364 - 0.563 ) |
|  | Poland | 158 (148 - 166) | 0.37 (0.347 - 0.391) | 202 (179 - 222) | 0.312 (0.278 - 0.343) | -0.585 (-1.004to-0.165) | -0.646  ( -1.154 to -0.136 ) |
|  | Romania | 98 (80 - 119) | 0.365 (0.298 - 0.441) | 198 (149 - 256) | 0.629 (0.469 - 0.824) | 1.754 (1.18-2.331) | 1.825  ( 1.568 - 2.082 ) |
|  | Serbia | 47 (33 - 66) | 0.421 (0.301 - 0.585) | 47 (34 - 65) | 0.317 (0.228 - 0.44) | -0.87 (-1.075to-0.665) | -0.888  ( -1.259 to -0.516 ) |
|  | Slovakia | 39 (30 - 49) | 0.682 (0.538 - 0.855) | 36 (26 - 51) | 0.419 (0.292 - 0.595) | -1.22 (-1.59to-0.85) | -1.543  ( -1.850 to -1.235 ) |
|  | Slovenia | 11 (9 - 13) | 0.435 (0.352 - 0.538) | 5 (4 - 7) | 0.13 (0.089 - 0.186) | -4.063 (-4.621to-3.502) | -3.896  ( -4.667 to -3.118 ) |
| **Eastern Europe** | Belarus | 46 (36 - 59) | 0.361 (0.288 - 0.461) | 43 (31 - 60) | 0.282 (0.201 - 0.388) | -1.184 (-1.444to-0.924) | -0.788  ( -1.297 to -0.277 ) |
|  | Estonia | 13 (10 - 17) | 0.663 (0.502 - 0.876) | 6 (4 - 8) | 0.247 (0.176 - 0.334) | -3.495 (-3.632to-3.356) | -3.520  ( -3.672 to -3.368 ) |
|  | Latvia | 24 (20 - 30) | 0.708 (0.571 - 0.879) | 9 (7 - 12) | 0.275 (0.198 - 0.374) | -3.758 (-4.085to-3.43) | -3.067  ( -4.120 to -2.003 ) |
|  | Lithuania | 28 (23 - 34) | 0.644 (0.528 - 0.774) | 12 (10 - 16) | 0.256 (0.195 - 0.325) | -2.97 (-3.283to-2.656) | -3.132  ( -3.991 to -2.264 ) |
|  | Republic of Moldova | 30 (26 - 34) | 0.675 (0.583 - 0.763) | 37 (30 - 45) | 0.647 (0.524 - 0.796) | -0.207 (-0.391to-0.021) | -0.222  ( -0.845 - 0.405 ) |
|  | Russian Federation | 546 (515 - 605) | 0.309 (0.291 - 0.342) | 599 (539 - 679) | 0.27 (0.243 - 0.305) | -1.026 (-1.309to-0.742) | -0.223  ( -1.315 - 0.882 ) |
|  | Ukraine | 186 (142 - 262) | 0.273 (0.208 - 0.382) | 256 (172 - 370) | 0.376 (0.254 - 0.54) | 0.846 (0.544-1.148) | 1.051  ( 0.208 - 1.900 ) |
| **Australasia** | Australia | 101 (90 - 112) | 0.529 (0.475 - 0.588) | 103 (80 - 129) | 0.254 (0.198 - 0.319) | -2.5 (-2.577to-2.423) | -2.364  ( -2.626 to -2.102 ) |
|  | New Zealand | 14 (11 - 16) | 0.36 (0.303 - 0.431) | 12 (10 - 16) | 0.165 (0.128 - 0.206) | -2.547 (-2.741to-2.353) | -2.521  ( -3.073 to -1.965 ) |
| **High-income Asia Pacific** | Brunei Darussalam | 4 (3 - 5) | 3.496 (2.758 - 4.3) | 10 (8 - 12) | 2.577 (2.078 - 3.143) | -0.25 (-0.462to-0.038) | -0.975  ( -1.112 to -0.838 ) |
|  | Japan | 427 (409 - 443) | 0.254 (0.244 - 0.264) | 955 (852 - 1025) | 0.271 (0.25 - 0.288) | -0.311 (-0.726-0.107) | 0.133  ( -0.153 - 0.420 ) |
|  | Republic of Korea | 160 (132 - 186) | 0.488 (0.404 - 0.565) | 206 (175 - 240) | 0.228 (0.194 - 0.266) | -2.409 (-2.576to-2.241) | -2.444  ( -2.591 to -2.296 ) |
|  | Singapore | 152 (134 - 172) | 5.891 (5.159 - 6.767) | 179 (137 - 225) | 2.123 (1.617 - 2.665) | -3.428 (-3.623to-3.232) | -3.207  ( -3.489 to -2.924 ) |
| **High-income North America** | Canada | 137 (121 - 153) | 0.44 (0.39 - 0.49) | 132 (102 - 168) | 0.207 (0.16 - 0.26) | -2.703 (-2.847to-2.559) | -2.356  ( -2.639 to -2.073 ) |
|  | Greenland | 4 (4 - 5) | 9.897 (8.52 - 11.362) | 3 (2 - 4) | 3.999 (3.2 - 5.023) | -2.988 (-3.112to-2.863) | -2.801  ( -3.121 to -2.480 ) |
|  | United States of America | 990 (952 - 1023) | 0.335 (0.324 - 0.346) | 1066 (1000 - 1115) | 0.201 (0.19 - 0.21) | -1.769 (-1.85to-1.687) | -1.648  ( -1.834 to -1.461 ) |
| **Southern Latin America** | Argentina | 119 (104 - 138) | 0.375 (0.327 - 0.432) | 99 (78 - 126) | 0.182 (0.143 - 0.232) | -1.975 (-2.102to-1.847) | -2.253  ( -2.616 to -1.888 ) |
|  | Chile | 19 (16 - 23) | 0.186 (0.157 - 0.221) | 19 (15 - 24) | 0.077 (0.06 - 0.098) | -2.415 (-2.674to-2.156) | -2.722  ( -3.264 to -2.178 ) |
|  | Uruguay | 21 (18 - 25) | 0.558 (0.482 - 0.661) | 16 (13 - 21) | 0.321 (0.25 - 0.404) | -1.655 (-1.842to-1.467) | -1.773  ( -2.254 to -1.289 ) |
| **Western Europe** | Andorra | 0 (0 - 0) | 0.253 (0.17 - 0.37) | 0 (0 - 0) | 0.131 (0.085 - 0.194) | -1.883 (-2.041to-1.724) | -2.279  ( -2.646 to -1.911 ) |
|  | Austria | 37 (32 - 42) | 0.347 (0.299 - 0.396) | 28 (22 - 35) | 0.173 (0.136 - 0.216) | -2.186 (-2.252to-2.12) | -2.248  ( -2.403 to -2.093 ) |
|  | Belgium | 60 (50 - 71) | 0.435 (0.362 - 0.514) | 54 (43 - 67) | 0.268 (0.215 - 0.331) | -1.777 (-2.035to-1.518) | -1.694  ( -2.009 to -1.379 ) |
|  | Cyprus | 2 (2 - 2) | 0.263 (0.207 - 0.323) | 3 (2 - 3) | 0.139 (0.109 - 0.175) | -1.894 (-1.971to-1.817) | -2.042  ( -2.331 to -1.752 ) |
|  | Denmark | 16 (13 - 18) | 0.218 (0.187 - 0.253) | 13 (10 - 17) | 0.121 (0.092 - 0.157) | -2.268 (-2.459to-2.076) | -1.820  ( -2.145 to -1.493 ) |
|  | Finland | 12 (10 - 14) | 0.176 (0.149 - 0.208) | 8 (6 - 11) | 0.078 (0.06 - 0.1) | -2.689 (-2.805to-2.572) | -2.555  ( -2.751 to -2.359 ) |
|  | France | 697 (618 - 798) | 0.964 (0.846 - 1.107) | 381 (297 - 485) | 0.326 (0.254 - 0.419) | -3.651 (-3.771to-3.53) | -3.455  ( -3.800 to -3.109 ) |
|  | Germany | 407 (357 - 464) | 0.358 (0.313 - 0.407) | 291 (221 - 372) | 0.173 (0.132 - 0.22) | -2.102 (-2.372to-1.831) | -2.416  ( -2.729 to -2.102 ) |
|  | Greece | 66 (60 - 72) | 0.46 (0.421 - 0.5) | 77 (68 - 88) | 0.386 (0.34 - 0.436) | -0.838 (-0.929to-0.747) | -0.574  ( -0.719 to -0.429 ) |
|  | Iceland | 1 (1 - 1) | 0.296 (0.25 - 0.349) | 1 (1 - 1) | 0.168 (0.132 - 0.217) | -2.095 (-2.237to-1.953) | -1.768  ( -2.093 to -1.443 ) |
|  | Ireland | 15 (13 - 17) | 0.381 (0.33 - 0.436) | 12 (9 - 15) | 0.162 (0.127 - 0.203) | -2.572 (-2.712to-2.433) | -2.734  ( -3.025 to -2.442 ) |
|  | Israel | 17 (14 - 21) | 0.376 (0.306 - 0.451) | 24 (18 - 30) | 0.205 (0.16 - 0.26) | -2.246 (-2.44to-2.052) | -2.013  ( -2.356 to -1.668 ) |
|  | Italy | 397 (370 - 425) | 0.491 (0.458 - 0.526) | 297 (262 - 336) | 0.246 (0.22 - 0.276) | -2.331 (-2.45to-2.213) | -2.193  ( -2.416 to -1.970 ) |
|  | Luxembourg | 3 (3 - 4) | 0.644 (0.573 - 0.715) | 3 (2 - 3) | 0.271 (0.233 - 0.321) | -2.919 (-3.032to-2.806) | -2.894  ( -3.228 to -2.559 ) |
|  | Malta | 5 (4 - 6) | 1.259 (1.065 - 1.491) | 6 (4 - 7) | 0.695 (0.529 - 0.894) | -1.996 (-2.065to-1.927) | -1.953  ( -2.271 to -1.633 ) |
|  | Monaco | 0 (0 - 0) | 0.255 (0.188 - 0.325) | 0 (0 - 0) | 0.211 (0.16 - 0.268) | -0.581 (-0.67to-0.491) | -0.612  ( -0.694 to -0.529 ) |
|  | Netherlands | 74 (66 - 84) | 0.397 (0.35 - 0.448) | 76 (60 - 93) | 0.237 (0.188 - 0.292) | -1.902 (-2.083to-1.721) | -1.675  ( -1.908 to -1.442 ) |
|  | Norway | 11 (11 - 12) | 0.192 (0.179 - 0.206) | 7 (7 - 9) | 0.08 (0.07 - 0.093) | -2.914 (-3.083to-2.743) | -2.802  ( -3.179 to -2.424 ) |
|  | Portugal | 78 (66 - 92) | 0.602 (0.505 - 0.703) | 70 (52 - 93) | 0.345 (0.256 - 0.463) | -1.999 (-2.097to-1.901) | -1.879  ( -2.326 to -1.430 ) |
|  | San Marino | 0 (0 - 0) | 0.682 (0.558 - 0.814) | 0 (0 - 0) | 0.305 (0.182 - 0.446) | -1.813 (-2.099to-1.526) | -2.881  ( -3.305 to -2.454 ) |
|  | Spain | 317 (283 - 354) | 0.634 (0.567 - 0.708) | 255 (203 - 318) | 0.297 (0.237 - 0.373) | -2.644 (-2.735to-2.552) | -2.520  ( -2.819 to -2.220 ) |
|  | Sweden | 20 (16 - 24) | 0.148 (0.122 - 0.176) | 16 (13 - 21) | 0.083 (0.065 - 0.105) | -1.96 (-2.22to-1.698) | -1.817  ( -2.733 to -0.892 ) |
|  | Switzerland | 53 (45 - 61) | 0.567 (0.486 - 0.648) | 27 (21 - 34) | 0.162 (0.127 - 0.207) | -4.461 (-5.137to-3.781) | -4.123  ( -4.593 to -3.652 ) |
|  | United Kingdom | 257 (248 - 265) | 0.315 (0.304 - 0.324) | 243 (228 - 255) | 0.211 (0.201 - 0.221) | -1.309 (-1.395to-1.224) | -1.312  ( -1.611 to -1.012 ) |
| **Andean Latin America** | Bolivia  (Plurinational State of) | 9 (7 - 11) | 0.256 (0.194 - 0.329) | 19 (14 - 26) | 0.205 (0.157 - 0.28) | -0.759 (-0.785to-0.733) | -0.720  ( -0.786 to -0.653 ) |
|  | Ecuador | 8 (7 - 10) | 0.146 (0.122 - 0.172) | 19 (13 - 25) | 0.117 (0.083 - 0.156) | -0.317 (-0.739-0.106) | -0.851  ( -1.293 to -0.406 ) |
|  | Peru | 18 (15 - 22) | 0.14 (0.119 - 0.168) | 37 (27 - 50) | 0.11 (0.08 - 0.146) | -0.914 (-1.072to-0.756) | -0.829  ( -2.192 - 0.554 ) |
| **Caribbean** | Antigua and Barbuda | 0 (0 - 0) | 0.312 (0.284 - 0.342) | 0 (0 - 0) | 0.363 (0.322 - 0.406) | 0.521 (0.342-0.7) | 0.669  ( -0.150 - 1.494 ) |
|  | Bahamas | 1 (1 - 1) | 0.492 (0.433 - 0.558) | 2 (2 - 3) | 0.575 (0.448 - 0.729) | 0.598 (0.503-0.694) | 0.564  ( 0.333 - 0.796 ) |
|  | Barbados | 1 (1 - 1) | 0.465 (0.418 - 0.525) | 3 (2 - 4) | 0.579 (0.449 - 0.729) | 0.725 (0.436-1.015) | 0.886  ( 0.242 - 1.534 ) |
|  | Belize | 0 (0 - 0) | 0.208 (0.19 - 0.227) | 1 (1 - 1) | 0.314 (0.266 - 0.369) | 1.26 (0.67-1.853) | 1.362  ( 0.946 - 1.780 ) |
|  | Bermuda | 0 (0 - 0) | 0.558 (0.475 - 0.655) | 1 (0 - 1) | 0.449 (0.338 - 0.603) | -0.557 (-0.64to-0.474) | -0.777  ( -1.153 to -0.399 ) |
|  | Cuba | 41 (36 - 46) | 0.404 (0.351 - 0.458) | 102 (79 - 131) | 0.529 (0.413 - 0.673) | 0.95 (0.852-1.048) | 1.050  ( 0.864 - 1.236 ) |
|  | Dominica | 0 (0 - 0) | 0.405 (0.357 - 0.453) | 0 (0 - 1) | 0.508 (0.419 - 0.627) | 0.848 (0.7-0.997) | 0.724  ( 0.560 - 0.887 ) |
|  | Dominican Republic | 18 (15 - 21) | 0.452 (0.373 - 0.544) | 41 (31 - 54) | 0.404 (0.303 - 0.527) | -0.059 (-0.241-0.124) | -0.315  ( -0.749 - 0.121 ) |
|  | Grenada | 0 (0 - 0) | 0.536 (0.433 - 0.665) | 1 (1 - 1) | 0.59 (0.474 - 0.74) | 0.557 (0.24-0.875) | 0.387  ( -0.086 - 0.863 ) |
|  | Guyana | 1 (1 - 1) | 0.226 (0.182 - 0.271) | 2 (1 - 3) | 0.3 (0.21 - 0.438) | 1.205 (0.943-1.467) | 1.109  ( 0.694 - 1.525 ) |
|  | Haiti | 26 (14 - 34) | 0.726 (0.412 - 0.948) | 55 (34 - 78) | 0.701 (0.442 - 0.99) | 0.045 (-0.034-0.124) | -0.043  ( -0.132 - 0.046 ) |
|  | Jamaica | 5 (4 - 7) | 0.296 (0.239 - 0.365) | 15 (10 - 20) | 0.466 (0.322 - 0.64) | 1.443 (0.988-1.901) | 2.159  ( 0.915 - 3.418 ) |
|  | Puerto Rico | 15 (13 - 18) | 0.437 (0.37 - 0.513) | 21 (16 - 26) | 0.324 (0.252 - 0.409) | -0.971 (-1.239to-0.703) | -0.996  ( -1.374 to -0.617 ) |
|  | Saint Kitts and Nevis | 0 (0 - 0) | 0.714 (0.621 - 0.825) | 0 (0 - 1) | 0.706 (0.574 - 0.851) | 0.264 (0.115-0.414) | -0.052  ( -0.552 - 0.451 ) |
|  | Saint Lucia | 1 (1 - 1) | 0.738 (0.664 - 0.807) | 2 (1 - 2) | 0.725 (0.58 - 0.882) | -0.328 (-0.46to-0.196) | -0.059  ( -0.276 - 0.158 ) |
|  | Saint Vincent and the Grenadines | 0 (0 - 0) | 0.519 (0.449 - 0.606) | 1 (1 - 1) | 0.622 (0.521 - 0.736) | 0.535 (0.424-0.646) | 0.508  ( 0.050 - 0.968 ) |
|  | Suriname | 2 (2 - 2) | 0.636 (0.555 - 0.719) | 4 (3 - 6) | 0.696 (0.524 - 0.869) | 0.492 (0.252-0.732) | 0.417  ( -0.104 - 0.941 ) |
|  | Trinidad and Tobago | 3 (3 - 3) | 0.351 (0.322 - 0.38) | 7 (5 - 9) | 0.38 (0.281 - 0.496) | 0.13 (-0.048-0.308) | 0.291  ( 0.059 - 0.523 ) |
|  | United States Virgin Islands | 1 (0 - 1) | 0.677 (0.563 - 0.806) | 1 (0 - 1) | 0.381 (0.299 - 0.491) | -1.56 (-1.779to-1.341) | -1.861  ( -2.136 to -1.585 ) |
| **Central Latin America** | Colombia | 61 (53 - 69) | 0.305 (0.268 - 0.348) | 93 (69 - 123) | 0.169 (0.126 - 0.223) | -2.504 (-2.715to-2.292) | -2.039  ( -2.284 to -1.793 ) |
|  | Costa Rica | 9 (8 - 11) | 0.499 (0.437 - 0.566) | 19 (16 - 24) | 0.345 (0.284 - 0.428) | -1.706 (-1.927to-1.484) | -1.241  ( -1.543 to -0.939 ) |
|  | El Salvador | 7 (6 - 7) | 0.208 (0.188 - 0.232) | 12 (10 - 15) | 0.199 (0.159 - 0.247) | -0.314 (-0.446to-0.182) | -0.138  ( -0.823 - 0.552 ) |
|  | Guatemala | 11 (10 - 12) | 0.294 (0.271 - 0.317) | 23 (19 - 27) | 0.199 (0.166 - 0.232) | -1.525 (-1.81to-1.238) | -1.376  ( -1.959 to -0.789 ) |
|  | Honduras | 5 (4 - 6) | 0.217 (0.176 - 0.26) | 15 (12 - 20) | 0.236 (0.187 - 0.301) | 0.382 (0.286-0.477) | 0.330  ( -0.000 - 0.661 ) |
|  | Mexico | 83 (80 - 86) | 0.184 (0.177 - 0.192) | 174 (148 - 199) | 0.136 (0.116 - 0.155) | -1.164 (-1.251to-1.078) | -1.029  ( -1.356 to -0.701 ) |
|  | Nicaragua | 3 (3 - 4) | 0.196 (0.165 - 0.228) | 9 (7 - 12) | 0.181 (0.145 - 0.231) | -0.183 (-0.486-0.121) | -0.248  ( -0.507 - 0.010 ) |
|  | Panama | 5 (4 - 5) | 0.305 (0.274 - 0.336) | 12 (9 - 15) | 0.269 (0.207 - 0.334) | -0.567 (-0.789to-0.345) | -0.367  ( -0.624 to -0.108 ) |
|  | Venezuela  (Bolivarian Republic of) | 25 (23 - 28) | 0.245 (0.219 - 0.272) | 83 (61 - 113) | 0.278 (0.203 - 0.377) | 0.024 (-0.254-0.302) | 0.449  ( 0.121 - 0.779 ) |
| **Tropical Latin America** | Brazil | 200 (186 - 216) | 0.191 (0.178 - 0.207) | 527 (484 - 575) | 0.208 (0.191 - 0.227) | -0.051 (-0.484-0.383) | 0.251  ( -0.084 - 0.587 ) |
|  | Paraguay | 3 (2 - 3) | 0.099 (0.08 - 0.121) | 10 (7 - 13) | 0.16 (0.116 - 0.211) | 1.567 (1.188-1.948) | 1.561  ( 1.222 - 1.900 ) |
| **North Africa and Middle East** | Afghanistan | 60 (28 - 87) | 0.826 (0.392 - 1.168) | 74 (36 - 107) | 0.573 (0.285 - 0.796) | -1.438 (-1.555to-1.322) | -1.176  ( -1.244 to -1.109 ) |
|  | Algeria | 357 (291 - 435) | 2.556 (2.075 - 3.132) | 693 (527 - 876) | 1.82 (1.393 - 2.303) | -0.997 (-1.081to-0.912) | -1.101  ( -1.167 to -1.036 ) |
|  | Bahrain | 2 (1 - 2) | 0.877 (0.721 - 1.065) | 4 (3 - 6) | 0.428 (0.309 - 0.59) | -2.864 (-3.168to-2.559) | -2.246  ( -2.826 to -1.663 ) |
|  | Egypt | 33 (28 - 38) | 0.11 (0.097 - 0.126) | 48 (38 - 60) | 0.071 (0.057 - 0.087) | -1.406 (-1.673to-1.14) | -1.426  ( -1.739 to -1.112 ) |
|  | Iran  (Islamic Republic of) | 62 (54 - 69) | 0.215 (0.19 - 0.244) | 107 (98 - 119) | 0.137 (0.125 - 0.152) | -1.476 (-1.622to-1.331) | -1.423  ( -1.604 to -1.242 ) |
|  | Iraq | 46 (37 - 58) | 0.493 (0.393 - 0.614) | 85 (63 - 113) | 0.299 (0.221 - 0.385) | -2.143 (-2.409to-1.877) | -1.607  ( -1.855 to -1.358 ) |
|  | Jordan | 15 (12 - 19) | 0.892 (0.72 - 1.086) | 38 (28 - 52) | 0.437 (0.322 - 0.59) | -2.8 (-3.096to-2.504) | -2.238  ( -2.462 to -2.013 ) |
|  | Kuwait | 6 (5 - 7) | 0.68 (0.564 - 0.809) | 7 (5 - 9) | 0.197 (0.151 - 0.255) | -3.364 (-3.801to-2.925) | -4.079  ( -5.534 to -2.602 ) |
|  | Lebanon | 12 (8 - 16) | 0.51 (0.362 - 0.695) | 18 (14 - 22) | 0.295 (0.234 - 0.367) | -1.623 (-1.755to-1.491) | -1.729  ( -1.887 to -1.570 ) |
|  | Libya | 58 (43 - 75) | 2.603 (1.914 - 3.353) | 151 (110 - 201) | 2.379 (1.753 - 3.168) | -0.192 (-0.286to-0.099) | -0.263  ( -0.447 to -0.080 ) |
|  | Morocco | 221 (178 - 268) | 1.383 (1.11 - 1.689) | 426 (303 - 563) | 1.164 (0.831 - 1.51) | -0.509 (-0.579to-0.438) | -0.564  ( -0.711 to -0.417 ) |
|  | Oman | 4 (3 - 6) | 0.51 (0.371 - 0.692) | 7 (5 - 9) | 0.26 (0.195 - 0.331) | -2.094 (-2.24to-1.948) | -2.121  ( -2.460 to -1.780 ) |
|  | Palestine | 3 (2 - 4) | 0.345 (0.264 - 0.449) | 6 (5 - 7) | 0.223 (0.185 - 0.266) | -1.506 (-1.719to-1.292) | -1.425  ( -1.626 to -1.223 ) |
|  | Qatar | 1 (1 - 1) | 0.491 (0.381 - 0.61) | 3 (2 - 5) | 0.244 (0.169 - 0.35) | -2.458 (-3.044to-1.869) | -2.278  ( -2.975 to -1.576 ) |
|  | Saudi Arabia | 111 (82 - 145) | 1.454 (1.088 - 1.858) | 252 (190 - 336) | 0.867 (0.68 - 1.09) | -1.969 (-2.102to-1.835) | -1.687  ( -1.801 to -1.572 ) |
|  | Sudan | 56 (35 - 82) | 0.526 (0.339 - 0.777) | 67 (47 - 94) | 0.288 (0.208 - 0.4) | -2.151 (-2.248to-2.053) | -1.931  ( -1.980 to -1.882 ) |
|  | Syrian Arab Republic | 10 (8 - 12) | 0.164 (0.127 - 0.202) | 13 (10 - 17) | 0.1 (0.076 - 0.13) | -2.131 (-2.415to-1.847) | -1.613  ( -1.898 to -1.327 ) |
|  | Tunisia | 140 (111 - 175) | 2.529 (2.003 - 3.147) | 247 (175 - 333) | 1.815 (1.297 - 2.43) | -1.3 (-1.39to-1.21) | -1.086  ( -1.199 to -0.973 ) |
|  | Türkiye | 344 (278 - 430) | 0.886 (0.715 - 1.111) | 435 (348 - 533) | 0.464 (0.372 - 0.565) | -2.298 (-2.46to-2.136) | -2.051  ( -2.285 to -1.817 ) |
|  | United Arab Emirates | 6 (4 - 9) | 0.834 (0.567 - 1.215) | 26 (20 - 34) | 0.512 (0.401 - 0.628) | -0.433 (-0.817to-0.048) | -1.439  ( -2.023 to -0.852 ) |
|  | Yemen | 29 (19 - 41) | 0.516 (0.354 - 0.725) | 56 (38 - 76) | 0.345 (0.242 - 0.469) | -1.537 (-1.651to-1.423) | -1.260  ( -1.405 to -1.114 ) |
| **South Asia** | Bangladesh | 941 (683 - 1173) | 1.656 (1.206 - 2.062) | 1348 (864 - 2034) | 0.916 (0.593 - 1.369) | -1.939 (-2.022to-1.857) | -1.833  ( -2.032 to -1.633 ) |
|  | Bhutan | 4 (3 - 6) | 1.43 (0.953 - 2.04) | 6 (4 - 9) | 0.877 (0.584 - 1.367) | -1.739 (-1.879to-1.6) | -1.570  ( -1.679 to -1.461 ) |
|  | India | 7581 (6424 - 8800) | 1.345 (1.134 - 1.564) | 12208 (10729 - 13744) | 0.946 (0.833 - 1.066) | -1.334 (-1.576to-1.091) | -1.111  ( -1.269 to -0.953 ) |
|  | Nepal | 151 (110 - 205) | 1.332 (0.976 - 1.792) | 244 (178 - 332) | 0.969 (0.704 - 1.309) | -0.91 (-1.195to-0.624) | -1.011  ( -1.103 to -0.920 ) |
|  | Pakistan | 882 (742 - 1062) | 1.359 (1.133 - 1.639) | 1978 (1554 - 2492) | 1.293 (1.025 - 1.644) | -0.431 (-0.596to-0.266) | -0.126  ( -0.204 to -0.048 ) |
| **East Asia** | China | 37625 (31890 - 43595) | 4.11 (3.49 - 4.735) | 31321 (25467 - 38381) | 1.508 (1.234 - 1.84) | -3.854 (-4.158to-3.548) | -3.212  ( -3.358 to -3.067 ) |
|  | Democratic People's Republic of Korea | 357 (249 - 468) | 1.959 (1.378 - 2.557) | 504 (380 - 662) | 1.486 (1.133 - 1.937) | -0.938 (-0.97to-0.905) | -0.885  ( -0.928 to -0.843 ) |
|  | Taiwan  (Province of China) | 853 (772 - 930) | 4.841 (4.405 - 5.265) | 791 (643 - 964) | 1.987 (1.612 - 2.427) | -3.299 (-3.45to-3.147) | -2.869  ( -3.059 to -2.679 ) |
| **Oceania** | American Samoa | 0 (0 - 0) | 1.363 (1.094 - 1.661) | 1 (1 - 1) | 1.44 (1.157 - 1.785) | 0.63 (0.448-0.811) | 0.169  ( -0.243 - 0.583 ) |
|  | Cook Islands | 0 (0 - 0) | 0.213 (0.168 - 0.262) | 0 (0 - 0) | 0.162 (0.124 - 0.207) | -0.743 (-0.909to-0.576) | -0.867  ( -1.186 to -0.546 ) |
|  | Fiji | 2 (1 - 2) | 0.37 (0.297 - 0.462) | 3 (2 - 4) | 0.303 (0.224 - 0.411) | -0.676 (-0.887to-0.465) | -0.644  ( -0.894 to -0.394 ) |
|  | Guam | 2 (2 - 2) | 2.338 (2.078 - 2.605) | 3 (2 - 3) | 1.341 (1.151 - 1.537) | -1.375 (-1.604to-1.146) | -1.783  ( -2.697 to -0.860 ) |
|  | Kiribati | 0 (0 - 0) | 0.844 (0.648 - 1.043) | 1 (0 - 1) | 0.79 (0.584 - 1.098) | -0.134 (-0.187to-0.08) | -0.211  ( -0.256 to -0.165 ) |
|  | Marshall Islands | 0 (0 - 0) | 1.22 (0.909 - 1.546) | 0 (0 - 1) | 1.07 (0.779 - 1.404) | -0.435 (-0.499to-0.371) | -0.426  ( -0.472 to -0.379 ) |
|  | Micronesia  (Federated States of) | 1 (1 - 1) | 1.371 (1.018 - 1.757) | 1 (1 - 1) | 1.004 (0.761 - 1.34) | -1.071 (-1.174to-0.967) | -1.002  ( -1.074 to -0.929 ) |
|  | Nauru | 0 (0 - 0) | 1.516 (1.018 - 2.065) | 0 (0 - 0) | 1.163 (0.737 - 1.697) | -0.904 (-1.05to-0.758) | -0.853  ( -0.919 to -0.788 ) |
|  | Niue | 0 (0 - 0) | 0.99 (0.744 - 1.261) | 0 (0 - 0) | 0.783 (0.59 - 1.009) | -1.026 (-1.124to-0.928) | -0.736  ( -0.841 to -0.631 ) |
|  | Northern Mariana Islands | 0 (0 - 0) | 1.595 (1.277 - 2.014) | 1 (1 - 1) | 1.393 (1.177 - 1.647) | -0.148 (-0.34-0.044) | -0.403  ( -0.943 - 0.139 ) |
|  | Palau | 0 (0 - 0) | 0.056 (0.037 - 0.086) | 0 (0 - 0) | 0.041 (0.029 - 0.057) | -0.761 (-0.912to-0.61) | -1.035  ( -1.250 to -0.820 ) |
|  | Papua New Guinea | 20 (12 - 30) | 0.942 (0.574 - 1.41) | 47 (28 - 75) | 0.779 (0.478 - 1.234) | -0.581 (-0.678to-0.484) | -0.574  ( -0.629 to -0.518 ) |
|  | Samoa | 2 (1 - 2) | 1.699 (1.355 - 2.102) | 2 (2 - 3) | 1.488 (1.153 - 1.936) | -0.446 (-0.537to-0.356) | -0.430  ( -0.454 to -0.405 ) |
|  | Solomon Islands | 2 (1 - 3) | 1.186 (0.698 - 1.704) | 4 (3 - 6) | 1.012 (0.725 - 1.375) | -0.453 (-0.58to-0.326) | -0.530  ( -0.671 to -0.389 ) |
|  | Tokelau | 0 (0 - 0) | 1.101 (0.786 - 1.472) | 0 (0 - 0) | 0.772 (0.543 - 1.047) | -1.287 (-1.36to-1.213) | -1.116  ( -1.191 to -1.041 ) |
|  | Tonga | 0 (0 - 1) | 0.799 (0.576 - 1.082) | 1 (0 - 1) | 0.671 (0.461 - 0.949) | -0.561 (-0.625to-0.497) | -0.557  ( -0.748 to -0.366 ) |
|  | Tuvalu | 0 (0 - 0) | 1.18 (0.875 - 1.502) | 0 (0 - 0) | 0.894 (0.723 - 1.124) | -0.869 (-0.933to-0.806) | -0.891  ( -0.934 to -0.847 ) |
|  | Vanuatu | 1 (1 - 1) | 1.045 (0.757 - 1.418) | 2 (1 - 2) | 0.876 (0.674 - 1.112) | -0.667 (-0.779to-0.556) | -0.588  ( -0.812 to -0.363 ) |
| **Southeast Asia** | Cambodia | 110 (83 - 139) | 2.146 (1.638 - 2.693) | 262 (192 - 349) | 1.967 (1.457 - 2.574) | -0.408 (-0.517to-0.3) | -0.264  ( -0.380 to -0.148 ) |
|  | Indonesia | 1663 (1320 - 2071) | 1.461 (1.166 - 1.809) | 3269 (2373 - 4341) | 1.27 (0.94 - 1.656) | -0.521 (-0.602to-0.44) | -0.441  ( -0.496 to -0.386 ) |
|  | Lao People's Democratic Republic | 54 (35 - 73) | 2.302 (1.506 - 3.093) | 80 (58 - 108) | 1.553 (1.132 - 2.06) | -1.425 (-1.499to-1.35) | -1.266  ( -1.308 to -1.223 ) |
|  | Malaysia | 713 (603 - 833) | 6.528 (5.494 - 7.616) | 1450 (1242 - 1718) | 4.764 (4.103 - 5.616) | -1.411 (-1.703to-1.118) | -1.003  ( -1.294 to -0.711 ) |
|  | Maldives | 0 (0 - 1) | 0.5 (0.346 - 0.636) | 1 (1 - 1) | 0.205 (0.16 - 0.255) | -3.299 (-3.451to-3.147) | -2.904  ( -3.140 to -2.667 ) |
|  | Mauritius | 6 (5 - 7) | 0.764 (0.695 - 0.845) | 13 (11 - 14) | 0.701 (0.609 - 0.793) | -0.54 (-0.779to-0.301) | -0.288  ( -0.956 - 0.384 ) |
|  | Myanmar | 510 (371 - 673) | 1.969 (1.453 - 2.587) | 649 (495 - 837) | 1.269 (0.977 - 1.627) | -1.701 (-1.806to-1.595) | -1.429  ( -1.517 to -1.341 ) |
|  | Philippines | 543 (477 - 625) | 1.591 (1.373 - 1.851) | 1357 (1127 - 1623) | 1.505 (1.253 - 1.792) | -0.117 (-0.208to-0.026) | -0.128  ( -0.187 to -0.070 ) |
|  | Seychelles | 1 (1 - 1) | 1.563 (1.322 - 1.826) | 2 (1 - 2) | 1.402 (1.162 - 1.659) | -0.332 (-0.628to-0.036) | -0.340  ( -0.562 to -0.118 ) |
|  | Sri Lanka | 119 (98 - 142) | 1.034 (0.855 - 1.237) | 169 (110 - 240) | 0.626 (0.41 - 0.879) | -1.704 (-1.98to-1.427) | -1.364  ( -1.756 to -0.970 ) |
|  | Thailand | 695 (583 - 808) | 1.726 (1.456 - 2.008) | 1312 (1001 - 1685) | 1.262 (0.967 - 1.606) | -1.495 (-1.684to-1.306) | -1.002  ( -1.139 to -0.864 ) |
|  | Timor-Leste | 5 (3 - 8) | 1.475 (0.992 - 2.172) | 12 (9 - 17) | 1.318 (0.958 - 1.831) | -0.374 (-0.551to-0.197) | -0.355  ( -0.535 to -0.174 ) |
|  | Viet Nam | 1181 (896 - 1532) | 2.747 (2.085 - 3.568) | 2585 (1878 - 3345) | 2.415 (1.777 - 3.111) | -0.356 (-0.427to-0.285) | -0.402  ( -0.469 to -0.335 ) |
| **Central Sub-Saharan Africa** | Angola | 20 (15 - 28) | 0.436 (0.316 - 0.595) | 55 (39 - 76) | 0.385 (0.271 - 0.532) | -0.392 (-0.447to-0.337) | -0.420  ( -0.686 to -0.153 ) |
|  | Central African Republic | 7 (5 - 9) | 0.532 (0.402 - 0.684) | 12 (8 - 18) | 0.448 (0.314 - 0.645) | -0.668 (-0.731to-0.605) | -0.525  ( -0.597 to -0.453 ) |
|  | Congo | 6 (5 - 8) | 0.525 (0.413 - 0.666) | 13 (10 - 18) | 0.416 (0.306 - 0.541) | -0.952 (-1.08to-0.823) | -0.737  ( -0.982 to -0.492 ) |
|  | Democratic Republic of the Congo | 69 (50 - 95) | 0.388 (0.28 - 0.541) | 156 (107 - 236) | 0.36 (0.248 - 0.547) | -0.229 (-0.328to-0.129) | -0.274  ( -0.388 to -0.159 ) |
|  | Equatorial Guinea | 1 (1 - 1) | 0.469 (0.342 - 0.63) | 2 (1 - 3) | 0.321 (0.203 - 0.485) | -1.429 (-1.678to-1.179) | -1.155  ( -1.381 to -0.928 ) |
|  | Gabon | 3 (2 - 4) | 0.498 (0.402 - 0.601) | 5 (3 - 6) | 0.388 (0.284 - 0.508) | -0.954 (-1.058to-0.85) | -0.787  ( -0.949 to -0.625 ) |
| **Eastern Sub-Saharan Africa** | Burundi | 55 (40 - 72) | 1.969 (1.431 - 2.602) | 89 (59 - 131) | 1.384 (0.93 - 2.009) | -1.491 (-1.651to-1.33) | -1.148  ( -1.329 to -0.967 ) |
|  | Comoros | 3 (2 - 5) | 1.423 (0.915 - 2.063) | 7 (4 - 10) | 1.212 (0.773 - 1.829) | -0.767 (-0.941to-0.593) | -0.563  ( -1.054 to -0.071 ) |
|  | Djibouti | 3 (2 - 4) | 1.407 (0.878 - 2.165) | 11 (6 - 18) | 1.297 (0.754 - 2.08) | -0.367 (-0.496to-0.238) | -0.251  ( -0.336 to -0.166 ) |
|  | Eritrea | 28 (23 - 36) | 1.763 (1.416 - 2.18) | 58 (41 - 81) | 1.538 (1.118 - 2.127) | -0.47 (-0.505to-0.435) | -0.490  ( -0.624 to -0.356 ) |
|  | Ethiopia | 493 (325 - 629) | 1.968 (1.312 - 2.503) | 695 (473 - 1062) | 1.267 (0.863 - 1.915) | -1.733 (-1.891to-1.574) | -1.409  ( -1.470 to -1.349 ) |
|  | Kenya | 170 (121 - 238) | 1.668 (1.176 - 2.356) | 527 (386 - 709) | 1.831 (1.359 - 2.453) | 0.578 (0.406-0.75) | 0.295  ( 0.203 - 0.387 ) |
|  | Madagascar | 82 (63 - 107) | 1.333 (0.996 - 1.748) | 161 (104 - 237) | 1.046 (0.684 - 1.517) | -0.801 (-0.903to-0.699) | -0.776  ( -0.847 to -0.704 ) |
|  | Malawi | 16 (13 - 21) | 0.333 (0.257 - 0.419) | 31 (22 - 43) | 0.311 (0.23 - 0.425) | -0.446 (-0.568to-0.323) | -0.177  ( -0.313 to -0.041 ) |
|  | Mozambique | 6 (5 - 8) | 0.091 (0.07 - 0.116) | 14 (10 - 18) | 0.104 (0.078 - 0.132) | 0.797 (0.665-0.929) | 0.431  ( 0.347 - 0.515 ) |
|  | Rwanda | 80 (61 - 99) | 2.245 (1.739 - 2.788) | 107 (72 - 155) | 1.365 (0.947 - 1.932) | -2.368 (-2.639to-2.097) | -1.574  ( -1.712 to -1.436 ) |
|  | Somalia | 55 (38 - 77) | 1.644 (1.14 - 2.285) | 129 (84 - 202) | 1.491 (1.001 - 2.298) | -0.329 (-0.379to-0.279) | -0.311  ( -0.372 to -0.249 ) |
|  | South Sudan | 40 (27 - 59) | 1.346 (0.892 - 2.001) | 65 (40 - 102) | 1.27 (0.812 - 1.975) | -0.34 (-0.533to-0.147) | -0.159  ( -0.265 to -0.053 ) |
|  | Uganda | 231 (174 - 302) | 2.979 (2.268 - 3.903) | 521 (375 - 722) | 2.637 (1.942 - 3.613) | -1.058 (-1.341to-0.774) | -0.405  ( -0.616 to -0.194 ) |
|  | United Republic of Tanzania | 206 (151 - 271) | 1.575 (1.168 - 2.079) | 396 (268 - 572) | 1.244 (0.856 - 1.763) | -0.872 (-0.92to-0.823) | -0.765  ( -0.876 to -0.654 ) |
|  | Zambia | 57 (45 - 72) | 1.578 (1.253 - 1.996) | 206 (84 - 362) | 2.118 (0.901 - 3.609) | 1.146 (0.884-1.408) | 1.019  ( 0.872 - 1.167 ) |
| **Southern Sub-Saharan Africa** | Botswana | 4 (3 - 6) | 0.701 (0.501 - 0.978) | 9 (6 - 14) | 0.56 (0.359 - 0.809) | -0.849 (-1.082to-0.616) | -0.757  ( -1.189 to -0.323 ) |
|  | Eswatini | 2 (2 - 3) | 0.712 (0.544 - 0.901) | 5 (3 - 7) | 0.79 (0.549 - 1.071) | 0.541 (0.089-0.994) | 0.347  ( 0.160 - 0.534 ) |
|  | Lesotho | 5 (4 - 7) | 0.564 (0.415 - 0.791) | 11 (8 - 15) | 0.896 (0.654 - 1.215) | 2.058 (1.674-2.443) | 1.571  ( 1.286 - 1.858 ) |
|  | Namibia | 5 (3 - 6) | 0.59 (0.45 - 0.765) | 9 (6 - 12) | 0.52 (0.372 - 0.683) | -0.763 (-1.112to-0.412) | -0.356  ( -0.561 to -0.151 ) |
|  | South Africa | 123 (102 - 146) | 0.543 (0.448 - 0.652) | 239 (212 - 264) | 0.492 (0.44 - 0.541) | -0.512 (-0.761to-0.262) | -0.217  ( -0.456 - 0.023 ) |
|  | Zimbabwe | 25 (20 - 32) | 0.542 (0.436 - 0.671) | 68 (50 - 88) | 0.762 (0.575 - 0.971) | 1.588 (1.155-2.023) | 1.107  ( 0.585 - 1.632 ) |
| **Western Sub-Saharan Africa** | Benin | 3 (2 - 4) | 0.122 (0.095 - 0.155) | 9 (6 - 13) | 0.123 (0.08 - 0.177) | -0.111 (-0.238-0.017) | 0.010  ( -0.252 - 0.272 ) |
|  | Burkina Faso | 7 (5 - 9) | 0.138 (0.103 - 0.181) | 19 (13 - 26) | 0.149 (0.102 - 0.205) | 0.184 (0.042-0.326) | 0.257  ( 0.069 - 0.446 ) |
|  | Cabo Verde | 0 (0 - 0) | 0.03 (0.024 - 0.037) | 1 (1 - 1) | 0.188 (0.141 - 0.243) | 4.754 (3.216-6.315) | 6.116  ( 5.895 - 6.338 ) |
|  | Cameroon | 8 (6 - 10) | 0.143 (0.111 - 0.182) | 29 (19 - 41) | 0.156 (0.103 - 0.216) | 0.172 (0.025-0.319) | 0.236  ( 0.161 - 0.312 ) |
|  | Chad | 4 (3 - 5) | 0.108 (0.079 - 0.143) | 13 (9 - 18) | 0.151 (0.104 - 0.212) | 1.081 (0.885-1.277) | 1.136  ( 0.977 - 1.295 ) |
|  | Côte d'Ivoire | 18 (14 - 24) | 0.332 (0.247 - 0.436) | 49 (32 - 70) | 0.313 (0.21 - 0.447) | -0.317 (-0.447to-0.187) | -0.189  ( -0.393 - 0.015 ) |
|  | Gambia | 1 (1 - 1) | 0.155 (0.116 - 0.198) | 2 (2 - 3) | 0.173 (0.127 - 0.227) | 0.106 (-0.067-0.279) | 0.337  ( -0.688 - 1.372 ) |
|  | Ghana | 7 (5 - 9) | 0.074 (0.052 - 0.102) | 3 (2 - 4) | 0.011 (0.008 - 0.016) | -8.731 (-10.316to-7.117) | -5.898  ( -6.490 to -5.303 ) |
|  | Guinea | 10 (8 - 13) | 0.274 (0.213 - 0.343) | 20 (15 - 28) | 0.291 (0.212 - 0.399) | 0.303 (0.223-0.383) | 0.194  ( 0.087 - 0.300 ) |
|  | Guinea-Bissau | 1 (1 - 1) | 0.187 (0.133 - 0.257) | 2 (2 - 3) | 0.202 (0.147 - 0.268) | 0.275 (0.198-0.351) | 0.264  ( 0.150 - 0.379 ) |
|  | Liberia | 2 (1 - 2) | 0.127 (0.091 - 0.173) | 5 (3 - 8) | 0.139 (0.089 - 0.214) | 0.177 (0.028-0.326) | 0.269  ( -0.094 - 0.633 ) |
|  | Mali | 8 (7 - 10) | 0.17 (0.14 - 0.209) | 17 (12 - 24) | 0.15 (0.105 - 0.212) | -0.31 (-0.47to-0.149) | -0.391  ( -0.482 to -0.299 ) |
|  | Mauritania | 1 (1 - 2) | 0.123 (0.091 - 0.167) | 3 (2 - 5) | 0.107 (0.062 - 0.172) | -0.625 (-0.699to-0.551) | -0.486  ( -0.624 to -0.348 ) |
|  | Niger | 4 (3 - 6) | 0.114 (0.078 - 0.16) | 13 (7 - 21) | 0.111 (0.063 - 0.183) | -0.131 (-0.234to-0.027) | -0.050  ( -0.170 - 0.071 ) |
|  | Nigeria | 547 (421 - 686) | 1.079 (0.835 - 1.349) | 1099 (740 - 1486) | 0.944 (0.66 - 1.253) | -0.5 (-0.604to-0.397) | -0.454  ( -0.537 to -0.372 ) |
|  | Sao Tome and Principe | 0 (0 - 0) | 0.022 (0.017 - 0.029) | 0 (0 - 0) | 0.021 (0.015 - 0.03) | -0.375 (-0.585to-0.164) | -0.143  ( -0.294 - 0.009 ) |
|  | Senegal | 5 (4 - 7) | 0.124 (0.088 - 0.17) | 13 (8 - 20) | 0.133 (0.082 - 0.201) | 0.272 (0.182-0.361) | 0.262  ( 0.170 - 0.353 ) |
|  | Sierra Leone | 3 (2 - 4) | 0.122 (0.089 - 0.161) | 7 (5 - 10) | 0.137 (0.091 - 0.193) | 0.508 (0.448-0.568) | 0.399  ( 0.172 - 0.627 ) |
|  | Togo | 2 (2 - 3) | 0.13 (0.1 - 0.167) | 9 (6 - 12) | 0.155 (0.109 - 0.216) | 0.518 (0.422-0.614) | 0.569  ( 0.461 - 0.677 ) |

*(Table continues on the next page)*

**Table S1 Global Burden of nasopharyngeal carcinoma and temporal trends from 1990 to 2021 across 204 countries or territories.** *(continued)*

| **GBD regions** | **Location** | **DALYs** | | | | | |
| --- | --- | --- | --- | --- | --- | --- | --- |
|  |  | **1990** | | **2021** | | **1990-2021** | |
|  |  | **Number** | **ASR (per 100,000)** | **Number** | **ASR (per 100,000)** | **EAPC (%)** | **AAPC (%)** |
| **Central Asia** | Armenia | 200 (161 - 239) | 6.367 (5.188 - 7.604) | 323 (262 - 389) | 8.308 (6.729 - 10.038) | 1.105 (0.676-1.535) | 0.971  ( 0.404 - 1.542 ) |
|  | Azerbaijan | 398 (267 - 578) | 6.32 (4.386 - 8.97) | 549 (356 - 801) | 4.845 (3.188 - 7.037) | -0.872 (-1.05to-0.694) | -0.883  ( -1.310 to -0.454 ) |
|  | Georgia | 682 (508 - 899) | 11.213 (8.35 - 14.741) | 702 (548 - 885) | 14.307 (10.985 - 17.984) | 1.112 (0.579-1.647) | 0.810  ( -0.131 - 1.759 ) |
|  | Kazakhstan | 2071 (1849 - 2473) | 14.006 (12.481 - 16.701) | 2550 (2023 - 3188) | 13.025 (10.347 - 16.276) | -0.476 (-0.687to-0.266) | -0.146  ( -0.601 - 0.311 ) |
|  | Kyrgyzstan | 443 (335 - 588) | 13.179 (9.922 - 17.756) | 884 (621 - 1205) | 14.589 (10.226 - 19.964) | 0.77 (0.576-0.964) | 0.366  ( 0.193 - 0.539 ) |
|  | Mongolia | 216 (135 - 337) | 14.572 (9.294 - 22.713) | 416 (265 - 597) | 13.643 (8.722 - 19.561) | -0.301 (-0.53to-0.071) | -0.254  ( -0.595 - 0.090 ) |
|  | Tajikistan | 786 (546 - 1099) | 21.502 (15.248 - 30.13) | 1194 (818 - 1747) | 14.484 (9.73 - 21.342) | -1.616 (-1.756to-1.475) | -1.269  ( -1.559 to -0.978 ) |
|  | Turkmenistan | 407 (350 - 465) | 14.957 (13.016 - 16.932) | 807 (610 - 1086) | 16.399 (12.474 - 21.837) | 0.308 (-0.149-0.766) | 0.382  ( -0.359 - 1.129 ) |
|  | Uzbekistan | 2192 (1534 - 3107) | 14.136 (9.935 - 19.976) | 4812 (3380 - 6776) | 14.938 (10.501 - 20.986) | 0.074 (-0.097-0.246) | 0.175  ( -0.203 - 0.555 ) |
| **Central Europe** | Albania | 365 (272 - 492) | 14.04 (10.583 - 18.743) | 429 (291 - 613) | 11.643 (7.822 - 16.773) | -0.259 (-0.582-0.065) | -0.729  ( -1.342 to -0.112 ) |
|  | Bosnia and Herzegovina | 186 (156 - 221) | 3.952 (3.324 - 4.673) | 245 (183 - 317) | 4.652 (3.441 - 6.05) | 1.064 (0.717-1.412) | 0.522  ( 0.130 - 0.915 ) |
|  | Bulgaria | 934 (768 - 1143) | 8.385 (6.958 - 10.192) | 1348 (1012 - 1789) | 12.575 (9.495 - 16.531) | 0.829 (0.486-1.174) | 1.431  ( 0.717 - 2.150 ) |
|  | Croatia | 763 (640 - 904) | 12.287 (10.376 - 14.565) | 567 (458 - 701) | 8.268 (6.663 - 10.338) | -1.241 (-1.708to-0.771) | -1.260  ( -2.275 to -0.234 ) |
|  | Czechia | 1879 (1559 - 2254) | 14.952 (12.414 - 17.885) | 1145 (809 - 1559) | 6.881 (4.871 - 9.421) | -2.624 (-2.798to-2.45) | -2.469  ( -2.595 to -2.342 ) |
|  | Hungary | 2244 (1944 - 2546) | 17.253 (14.949 - 19.527) | 2287 (1790 - 2916) | 15.23 (12.125 - 19.158) | -0.534 (-0.971to-0.094) | -0.482  ( -1.049 - 0.089 ) |
|  | Montenegro | 23 (20 - 28) | 3.536 (2.994 - 4.209) | 29 (22 - 36) | 3.289 (2.543 - 4.176) | -0.275 (-0.464to-0.085) | -0.180  ( -0.403 - 0.044 ) |
|  | North Macedonia | 197 (162 - 243) | 9.662 (7.95 - 11.871) | 288 (211 - 399) | 9.129 (6.697 - 12.638) | -0.055 (-0.376-0.267) | -0.109  ( -0.393 - 0.176 ) |
|  | Poland | 5096 (4812 - 5380) | 12.065 (11.394 - 12.737) | 5678 (5061 - 6234) | 9.713 (8.67 - 10.673) | -0.733 (-1.165to-0.3) | -0.781  ( -1.343 to -0.215 ) |
|  | Romania | 3358 (2695 - 4070) | 12.607 (10.113 - 15.252) | 6032 (4487 - 7851) | 21.015 (15.502 - 27.533) | 1.65 (1.053-2.251) | 1.686  ( 1.366 - 2.007 ) |
|  | Serbia | 1550 (1075 - 2181) | 13.417 (9.359 - 18.601) | 1320 (936 - 1851) | 9.754 (6.873 - 13.723) | -0.966 (-1.189to-0.742) | -0.993  ( -1.351 to -0.633 ) |
|  | Slovakia | 1335 (1040 - 1700) | 23.733 (18.572 - 30.211) | 1102 (766 - 1580) | 13.616 (9.391 - 19.689) | -1.429 (-1.812to-1.044) | -1.748  ( -2.100 to -1.395 ) |
|  | Slovenia | 343 (276 - 425) | 14.437 (11.62 - 17.83) | 135 (93 - 194) | 3.93 (2.702 - 5.652) | -4.362 (-4.949to-3.772) | -4.316  ( -4.950 to -3.679 ) |
| **Eastern Europe** | Belarus | 1558 (1245 - 1980) | 12.679 (10.145 - 15.967) | 1294 (924 - 1777) | 9.083 (6.63 - 12.309) | -1.501 (-1.788to-1.213) | -1.031  ( -1.622 to -0.436 ) |
|  | Estonia | 432 (328 - 572) | 22.698 (17.219 - 30.032) | 154 (110 - 208) | 7.629 (5.431 - 10.332) | -3.885 (-4.046to-3.725) | -3.602  ( -4.218 to -2.983 ) |
|  | Latvia | 809 (657 - 1002) | 24.646 (19.922 - 30.602) | 260 (188 - 352) | 8.82 (6.389 - 11.915) | -4.103 (-4.446to-3.759) | -3.354  ( -4.465 to -2.230 ) |
|  | Lithuania | 958 (776 - 1161) | 22.499 (18.246 - 27.29) | 356 (270 - 455) | 8.26 (6.256 - 10.682) | -3.261 (-3.608to-2.912) | -3.356  ( -4.441 to -2.259 ) |
|  | Republic of Moldova | 1043 (897 - 1177) | 22.778 (19.625 - 25.692) | 1121 (906 - 1384) | 20.951 (16.951 - 25.848) | -0.327 (-0.526to-0.128) | -0.357  ( -1.014 - 0.303 ) |
|  | Russian Federation | 18597 (17540 - 20728) | 10.735 (10.121 - 11.983) | 18683 (16769 - 21182) | 9.024 (8.128 - 10.197) | -1.144 (-1.431to-0.857) | -0.354  ( -1.435 - 0.738 ) |
|  | Ukraine | 6445 (4911 - 9040) | 9.978 (7.618 - 13.78) | 8434 (5621 - 12149) | 13.511 (9.129 - 19.268) | 0.748 (0.454-1.042) | 0.993  ( 0.150 - 1.843 ) |
| **Australasia** | Australia | 3141 (2804 - 3484) | 16.839 (15.042 - 18.699) | 2936 (2272 - 3690) | 8.163 (6.266 - 10.34) | -2.452 (-2.531to-2.372) | -2.318  ( -2.596 to -2.039 ) |
|  | New Zealand | 443 (374 - 534) | 12.153 (10.249 - 14.705) | 376 (292 - 470) | 5.509 (4.278 - 6.894) | -2.559 (-2.751to-2.367) | -2.559  ( -3.144 to -1.971 ) |
| **High-income Asia Pacific** | Brunei Darussalam | 166 (133 - 202) | 106.19 (84.08 - 128.821) | 354 (287 - 431) | 77.669 (62.816 - 94.579) | -0.338 (-0.54to-0.135) | -0.982  ( -1.199 to -0.765 ) |
|  | Japan | 12539 (12101 - 12967) | 7.573 (7.309 - 7.827) | 18684 (17223 - 19866) | 6.998 (6.606 - 7.401) | -0.773 (-1.191to-0.354) | -0.339  ( -0.637 to -0.039 ) |
|  | Republic of Korea | 5676 (4692 - 6588) | 15.06 (12.453 - 17.439) | 5645 (4774 - 6544) | 6.631 (5.615 - 7.666) | -2.613 (-2.785to-2.442) | -2.630  ( -2.794 to -2.465 ) |
|  | Singapore | 5575 (4967 - 6259) | 195.385 (173.105 - 220.528) | 4879 (3777 - 6101) | 59.026 (45.545 - 73.988) | -4.029 (-4.254to-3.802) | -3.786  ( -4.164 to -3.407 ) |
| **High-income North America** | Canada | 4324 (3834 - 4792) | 14.177 (12.588 - 15.715) | 3719 (2888 - 4686) | 6.607 (5.154 - 8.311) | -2.763 (-2.917to-2.609) | -2.375  ( -2.711 to -2.037 ) |
|  | Greenland | 159 (135 - 188) | 330.13 (282.134 - 383.67) | 94 (75 - 118) | 124.028 (99.199 - 154.989) | -3.189 (-3.32to-3.058) | -3.036  ( -3.349 to -2.722 ) |
|  | United States of America | 30674 (29706 - 31668) | 10.918 (10.588 - 11.265) | 30867 (29522 - 32235) | 6.45 (6.182 - 6.737) | -1.802 (-1.884to-1.721) | -1.722  ( -1.897 to -1.547 ) |
| **Southern Latin America** | Argentina | 3744 (3279 - 4279) | 11.576 (10.138 - 13.263) | 2822 (2211 - 3572) | 5.421 (4.241 - 6.855) | -2.114 (-2.225to-2.003) | -2.405  ( -2.732 to -2.076 ) |
|  | Chile | 639 (544 - 751) | 5.705 (4.851 - 6.709) | 541 (426 - 684) | 2.272 (1.788 - 2.874) | -2.541 (-2.806to-2.274) | -2.913  ( -3.612 to -2.209 ) |
|  | Uruguay | 609 (527 - 720) | 17.182 (14.814 - 20.252) | 440 (339 - 556) | 9.783 (7.501 - 12.423) | -1.69 (-1.886to-1.493) | -1.816  ( -2.316 to -1.314 ) |
| **Western Europe** | Andorra | 5 (3 - 7) | 8.111 (5.479 - 11.702) | 6 (4 - 9) | 4.185 (2.681 - 6.295) | -1.877 (-2.023to-1.731) | -2.278  ( -2.563 to -1.991 ) |
|  | Austria | 1100 (942 - 1254) | 11.22 (9.607 - 12.785) | 749 (588 - 934) | 5.232 (4.103 - 6.559) | -2.408 (-2.473to-2.342) | -2.469  ( -2.653 to -2.285 ) |
|  | Belgium | 1805 (1503 - 2129) | 14.051 (11.637 - 16.595) | 1471 (1180 - 1815) | 8.364 (6.653 - 10.3) | -1.966 (-2.215to-1.717) | -1.859  ( -2.217 to -1.499 ) |
|  | Cyprus | 57 (45 - 71) | 7.184 (5.647 - 8.957) | 76 (58 - 97) | 4.04 (3.091 - 5.221) | -1.658 (-1.733to-1.583) | -1.858  ( -2.099 to -1.618 ) |
|  | Denmark | 463 (399 - 537) | 6.97 (5.996 - 8.082) | 335 (255 - 432) | 3.609 (2.741 - 4.657) | -2.467 (-2.663to-2.271) | -2.063  ( -2.473 to -1.652 ) |
|  | Finland | 360 (306 - 422) | 5.686 (4.834 - 6.668) | 213 (163 - 273) | 2.463 (1.881 - 3.159) | -2.758 (-2.905to-2.61) | -2.657  ( -2.820 to -2.494 ) |
|  | France | 22180 (19611 - 25333) | 32.46 (28.381 - 37.225) | 10442 (8151 - 13437) | 10.509 (8.184 - 13.576) | -3.828 (-3.96to-3.696) | -3.542  ( -3.863 to -3.219 ) |
|  | Germany | 12933 (11288 - 14651) | 12.114 (10.585 - 13.676) | 7528 (5700 - 9594) | 5.225 (4.042 - 6.629) | -2.565 (-2.8to-2.329) | -2.878  ( -3.229 to -2.525 ) |
|  | Greece | 1944 (1775 - 2110) | 14.339 (13.098 - 15.537) | 2011 (1780 - 2265) | 11.906 (10.51 - 13.335) | -0.831 (-0.902to-0.761) | -0.594  ( -0.783 to -0.406 ) |
|  | Iceland | 24 (21 - 29) | 9.389 (7.912 - 11.108) | 25 (20 - 32) | 5.288 (4.185 - 6.785) | -2.152 (-2.323to-1.982) | -1.788  ( -2.029 to -1.547 ) |
|  | Ireland | 434 (377 - 498) | 11.602 (10.072 - 13.335) | 342 (271 - 430) | 5.009 (3.968 - 6.293) | -2.517 (-2.63to-2.405) | -2.680  ( -3.071 to -2.286 ) |
|  | Israel | 528 (428 - 641) | 11.524 (9.343 - 14.002) | 663 (516 - 844) | 6.212 (4.835 - 7.919) | -2.258 (-2.472to-2.043) | -2.029  ( -2.366 to -1.690 ) |
|  | Italy | 12223 (11457 - 13116) | 16.303 (15.28 - 17.486) | 7855 (7031 - 8799) | 7.759 (6.977 - 8.703) | -2.521 (-2.644to-2.397) | -2.358  ( -2.645 to -2.069 ) |
|  | Luxembourg | 102 (91 - 114) | 21.077 (18.705 - 23.65) | 77 (66 - 92) | 8.1 (6.947 - 9.615) | -3.265 (-3.382to-3.149) | -3.072  ( -3.369 to -2.773 ) |
|  | Malta | 161 (137 - 191) | 38.553 (32.749 - 45.548) | 152 (116 - 195) | 22.481 (17.345 - 28.633) | -1.82 (-1.882to-1.759) | -1.762  ( -2.180 to -1.343 ) |
|  | Monaco | 4 (3 - 5) | 8.093 (6.083 - 10.412) | 4 (3 - 5) | 6.818 (5.147 - 8.709) | -0.536 (-0.634to-0.438) | -0.554  ( -0.640 to -0.467 ) |
|  | Netherlands | 2236 (1968 - 2526) | 12.523 (11.019 - 14.177) | 1967 (1553 - 2443) | 7.025 (5.565 - 8.816) | -2.092 (-2.282to-1.901) | -1.893  ( -2.109 to -1.676 ) |
|  | Norway | 320 (299 - 344) | 5.974 (5.571 - 6.43) | 190 (166 - 218) | 2.356 (2.059 - 2.714) | -3.09 (-3.263to-2.916) | -2.975  ( -3.451 to -2.496 ) |
|  | Portugal | 2453 (2065 - 2871) | 19.956 (16.75 - 23.394) | 1923 (1418 - 2579) | 11.011 (8.144 - 14.689) | -2.152 (-2.276to-2.027) | -1.936  ( -2.325 to -1.546 ) |
|  | San Marino | 6 (5 - 8) | 20.873 (16.723 - 25.261) | 5 (3 - 8) | 9.589 (5.552 - 14.324) | -1.747 (-2.019to-1.475) | -2.758  ( -3.060 to -2.455 ) |
|  | Spain | 9895 (8837 - 11092) | 21.109 (18.873 - 23.606) | 6869 (5475 - 8644) | 9.168 (7.318 - 11.525) | -2.958 (-3.075to-2.841) | -2.783  ( -3.123 to -2.442 ) |
|  | Sweden | 537 (440 - 637) | 4.575 (3.754 - 5.441) | 392 (303 - 495) | 2.457 (1.903 - 3.108) | -1.989 (-2.241to-1.737) | -1.973  ( -2.889 to -1.048 ) |
|  | Switzerland | 1598 (1377 - 1823) | 18.337 (15.87 - 20.901) | 685 (540 - 871) | 4.777 (3.796 - 6.028) | -4.818 (-5.493to-4.138) | -4.420  ( -4.952 to -3.885 ) |
|  | United Kingdom | 7229 (7021 - 7431) | 9.851 (9.583 - 10.122) | 6510 (6243 - 6809) | 6.595 (6.324 - 6.881) | -1.299 (-1.377to-1.222) | -1.330  ( -1.696 to -0.963 ) |
| **Andean Latin America** | Bolivia  (Plurinational State of) | 323 (247 - 405) | 7.767 (5.962 - 9.845) | 586 (434 - 799) | 5.759 (4.31 - 7.792) | -1.085 (-1.131to-1.038) | -0.960  ( -1.066 to -0.854 ) |
|  | Ecuador | 290 (245 - 340) | 4.243 (3.583 - 4.946) | 534 (378 - 705) | 3.137 (2.231 - 4.138) | -0.667 (-1.125to-0.208) | -1.076  ( -1.559 to -0.591 ) |
|  | Peru | 616 (528 - 739) | 4.061 (3.46 - 4.914) | 1038 (767 - 1389) | 2.967 (2.188 - 3.979) | -1.141 (-1.299to-0.982) | -0.952  ( -2.013 - 0.121 ) |
| **Caribbean** | Antigua and Barbuda | 5 (4 - 5) | 8.842 (8.026 - 9.703) | 11 (9 - 12) | 9.643 (8.589 - 10.749) | 0.363 (0.203-0.523) | 0.365  ( -0.019 - 0.751 ) |
|  | Bahamas | 29 (25 - 33) | 15.387 (13.516 - 17.493) | 77 (60 - 100) | 17.482 (13.516 - 22.484) | 0.502 (0.414-0.591) | 0.470  ( 0.228 - 0.713 ) |
|  | Barbados | 34 (31 - 39) | 13.364 (11.931 - 15.103) | 73 (56 - 93) | 15.809 (12.106 - 20.284) | 0.589 (0.294-0.886) | 0.703  ( 0.119 - 1.291 ) |
|  | Belize | 7 (6 - 8) | 6.203 (5.641 - 6.807) | 34 (28 - 39) | 9.338 (7.907 - 10.976) | 1.285 (0.712-1.86) | 1.347  ( 0.979 - 1.717 ) |
|  | Bermuda | 10 (8 - 12) | 15.499 (12.978 - 18.514) | 14 (10 - 19) | 12.24 (9.06 - 16.884) | -0.635 (-0.717to-0.553) | -0.757  ( -0.931 to -0.583 ) |
|  | Cuba | 1184 (1043 - 1320) | 11.32 (9.923 - 12.659) | 2700 (2117 - 3441) | 14.923 (11.852 - 18.938) | 0.916 (0.781-1.051) | 1.141  ( 0.872 - 1.411 ) |
|  | Dominica | 7 (6 - 7) | 11.095 (9.807 - 12.488) | 12 (10 - 15) | 14.538 (11.646 - 18.252) | 1.042 (0.894-1.189) | 0.866  ( 0.743 - 0.990 ) |
|  | Dominican Republic | 637 (538 - 766) | 13.167 (11.001 - 15.911) | 1269 (957 - 1666) | 11.971 (9.007 - 15.693) | -0.019 (-0.16-0.123) | -0.176  ( -0.716 - 0.367 ) |
|  | Grenada | 11 (9 - 14) | 16.29 (13.141 - 20.304) | 20 (16 - 25) | 16.692 (13.375 - 20.997) | 0.395 (0.142-0.65) | 0.191  ( -0.195 - 0.579 ) |
|  | Guyana | 33 (27 - 40) | 6.83 (5.449 - 8.278) | 69 (47 - 103) | 9.368 (6.468 - 13.956) | 1.351 (1.071-1.631) | 1.072  ( 0.514 - 1.632 ) |
|  | Haiti | 930 (486 - 1250) | 22.241 (11.971 - 29.514) | 1953 (1197 - 2795) | 20.825 (12.793 - 29.677) | -0.035 (-0.112-0.042) | -0.129  ( -0.275 - 0.016 ) |
|  | Jamaica | 149 (120 - 183) | 8.205 (6.581 - 10.102) | 403 (276 - 565) | 13.081 (8.94 - 18.3) | 1.405 (0.891-1.922) | 2.218  ( 0.931 - 3.522 ) |
|  | Puerto Rico | 450 (384 - 526) | 12.619 (10.782 - 14.751) | 514 (398 - 649) | 9.699 (7.516 - 12.182) | -0.914 (-1.189to-0.639) | -0.872  ( -1.244 to -0.499 ) |
|  | Saint Kitts and Nevis | 7 (6 - 8) | 20.809 (18.158 - 23.941) | 14 (11 - 18) | 19.184 (15.476 - 23.629) | -0.059 (-0.179-0.061) | -0.287  ( -0.781 - 0.209 ) |
|  | Saint Lucia | 19 (17 - 21) | 20.59 (18.494 - 22.703) | 49 (38 - 60) | 20.869 (16.588 - 25.501) | -0.116 (-0.223to-0.009) | 0.112  ( -0.088 - 0.313 ) |
|  | Saint Vincent and the Grenadines | 12 (10 - 13) | 14.861 (12.949 - 17.34) | 25 (21 - 30) | 18.347 (15.322 - 22.035) | 0.592 (0.47-0.715) | 0.657  ( 0.449 - 0.865 ) |
|  | Suriname | 58 (49 - 66) | 19.207 (16.492 - 21.62) | 139 (105 - 176) | 21.32 (16.168 - 26.802) | 0.469 (0.251-0.688) | 0.438  ( -0.051 - 0.930 ) |
|  | Trinidad and Tobago | 92 (85 - 100) | 9.996 (9.181 - 10.872) | 210 (154 - 276) | 11.564 (8.464 - 15.158) | 0.316 (0.113-0.519) | 0.511  ( 0.269 - 0.754 ) |
|  | United States Virgin Islands | 19 (15 - 23) | 19.53 (16.127 - 23.232) | 15 (12 - 20) | 12.07 (9.262 - 15.584) | -1.116 (-1.296to-0.934) | -1.498  ( -1.777 to -1.217 ) |
| **Central Latin America** | Colombia | 2236 (1960 - 2550) | 9.56 (8.389 - 10.953) | 2690 (2015 - 3562) | 4.937 (3.7 - 6.547) | -2.733 (-2.938to-2.528) | -2.261  ( -2.523 to -1.999 ) |
|  | Costa Rica | 312 (272 - 357) | 15.085 (13.15 - 17.312) | 549 (446 - 681) | 10.101 (8.226 - 12.514) | -1.95 (-2.198to-1.701) | -1.378  ( -1.699 to -1.056 ) |
|  | El Salvador | 240 (219 - 264) | 6.518 (5.917 - 7.215) | 383 (303 - 476) | 6.198 (4.882 - 7.723) | -0.299 (-0.425to-0.173) | -0.316  ( -1.190 - 0.566 ) |
|  | Guatemala | 441 (405 - 479) | 8.78 (8.06 - 9.496) | 768 (630 - 910) | 6.003 (4.936 - 7.113) | -1.442 (-1.718to-1.164) | -1.342  ( -1.940 to -0.740 ) |
|  | Honduras | 191 (157 - 228) | 6.717 (5.476 - 7.983) | 480 (368 - 624) | 6.534 (5.172 - 8.358) | -0.072 (-0.142to-0.001) | -0.068  ( -0.385 - 0.249 ) |
|  | Mexico | 2861 (2757 - 2969) | 5.235 (5.043 - 5.443) | 5315 (4552 - 6086) | 3.982 (3.412 - 4.555) | -1.074 (-1.159to-0.989) | -0.918  ( -1.264 to -0.572 ) |
|  | Nicaragua | 130 (112 - 149) | 5.903 (5.039 - 6.83) | 296 (235 - 373) | 5.309 (4.196 - 6.713) | -0.298 (-0.568to-0.028) | -0.244  ( -0.426 to -0.061 ) |
|  | Panama | 152 (137 - 169) | 8.69 (7.79 - 9.618) | 341 (260 - 423) | 7.718 (5.89 - 9.59) | -0.54 (-0.766to-0.315) | -0.411  ( -0.689 to -0.133 ) |
|  | Venezuela  (Bolivarian Republic of) | 869 (785 - 951) | 7.11 (6.402 - 7.814) | 2538 (1825 - 3444) | 8.434 (6.068 - 11.425) | 0.192 (-0.079-0.462) | 0.622  ( 0.244 - 1.001 ) |
| **Tropical Latin America** | Brazil | 7918 (7370 - 8559) | 6.801 (6.335 - 7.345) | 18388 (16916 - 20040) | 7.297 (6.71 - 7.946) | -0.076 (-0.516-0.366) | 0.195  ( -0.135 - 0.526 ) |
|  | Paraguay | 97 (80 - 119) | 3.423 (2.784 - 4.219) | 356 (261 - 474) | 5.413 (3.942 - 7.217) | 1.46 (1.062-1.859) | 1.661  ( 1.381 - 1.942 ) |
| **North Africa and Middle East** | Afghanistan | 2125 (910 - 3123) | 27.346 (11.645 - 40.144) | 3081 (1518 - 4442) | 18.402 (8.78 - 26.267) | -1.548 (-1.669to-1.427) | -1.274  ( -1.359 to -1.188 ) |
|  | Algeria | 14403 (11753 - 17561) | 85.025 (68.754 - 104.037) | 24517 (18714 - 31423) | 58.346 (44.515 - 73.417) | -1.183 (-1.271to-1.096) | -1.217  ( -1.280 to -1.154 ) |
|  | Bahrain | 69 (57 - 84) | 24.645 (20.216 - 29.942) | 159 (111 - 219) | 11.667 (8.23 - 16.252) | -2.962 (-3.259to-2.664) | -2.334  ( -2.558 to -2.109 ) |
|  | Egypt | 1243 (1084 - 1433) | 3.334 (2.881 - 3.859) | 1689 (1323 - 2128) | 2.082 (1.651 - 2.604) | -1.524 (-1.808to-1.239) | -1.512  ( -1.790 to -1.233 ) |
|  | Iran  (Islamic Republic of) | 2360 (2058 - 2631) | 6.443 (5.645 - 7.222) | 3409 (3120 - 3796) | 3.957 (3.613 - 4.389) | -1.576 (-1.742to-1.411) | -1.571  ( -1.810 to -1.332 ) |
|  | Iraq | 1840 (1437 - 2295) | 16.94 (13.347 - 21.311) | 3181 (2348 - 4215) | 9.728 (7.141 - 12.889) | -2.278 (-2.556to-2) | -1.793  ( -2.046 to -1.539 ) |
|  | Jordan | 614 (495 - 756) | 29.159 (23.45 - 35.807) | 1417 (1034 - 1957) | 13.748 (10.104 - 18.668) | -2.965 (-3.267to-2.662) | -2.391  ( -2.589 to -2.194 ) |
|  | Kuwait | 235 (193 - 280) | 21.737 (18.071 - 25.826) | 252 (189 - 331) | 5.665 (4.333 - 7.34) | -3.738 (-4.17to-3.305) | -4.447  ( -5.924 to -2.947 ) |
|  | Lebanon | 416 (289 - 573) | 16.55 (11.548 - 22.848) | 530 (418 - 664) | 9.078 (7.113 - 11.387) | -1.83 (-1.935to-1.725) | -1.881  ( -2.028 to -1.734 ) |
|  | Libya | 2234 (1653 - 2860) | 87.097 (64.089 - 112.319) | 5637 (4086 - 7580) | 79.027 (58.151 - 104.05) | -0.219 (-0.299to-0.139) | -0.314  ( -0.505 to -0.124 ) |
|  | Morocco | 8184 (6600 - 9887) | 46.36 (37.398 - 56.284) | 14120 (10039 - 19215) | 36.947 (26.361 - 49.695) | -0.687 (-0.733to-0.64) | -0.719  ( -0.779 to -0.659 ) |
|  | Oman | 170 (121 - 231) | 16.285 (11.691 - 22.28) | 269 (188 - 364) | 7.943 (5.737 - 10.41) | -2.22 (-2.375to-2.064) | -2.291  ( -2.751 to -1.829 ) |
|  | Palestine | 113 (84 - 149) | 10.071 (7.463 - 13.314) | 218 (178 - 259) | 6.35 (5.226 - 7.598) | -1.583 (-1.763to-1.404) | -1.491  ( -1.699 to -1.282 ) |
|  | Qatar | 30 (23 - 39) | 13.239 (10.233 - 16.47) | 134 (89 - 201) | 6.563 (4.454 - 9.702) | -2.329 (-2.874to-1.78) | -2.263  ( -2.823 to -1.700 ) |
|  | Saudi Arabia | 4448 (3266 - 5836) | 48.371 (35.729 - 63.103) | 10228 (7583 - 13801) | 28.29 (21.764 - 36.783) | -2.007 (-2.158to-1.856) | -1.749  ( -1.878 to -1.620 ) |
|  | Sudan | 2118 (1290 - 3089) | 17.083 (10.549 - 25.165) | 2593 (1757 - 3673) | 9.028 (6.317 - 12.662) | -2.253 (-2.343to-2.164) | -2.036  ( -2.092 to -1.979 ) |
|  | Syrian Arab Republic | 395 (314 - 479) | 5.237 (4.083 - 6.404) | 439 (323 - 582) | 2.955 (2.197 - 3.899) | -2.369 (-2.672to-2.066) | -1.849  ( -1.968 to -1.729 ) |
|  | Tunisia | 5065 (4069 - 6220) | 82.408 (65.818 - 102.497) | 8163 (5766 - 11064) | 59.311 (42.096 - 80.041) | -1.28 (-1.364to-1.197) | -1.067  ( -1.171 to -0.963 ) |
|  | Türkiye | 12843 (10424 - 15911) | 28.991 (23.506 - 36.021) | 13386 (10683 - 16377) | 13.968 (11.188 - 17.01) | -2.57 (-2.695to-2.445) | -2.358  ( -2.718 to -1.997 ) |
|  | United Arab Emirates | 264 (178 - 387) | 25.716 (17.303 - 38.05) | 1064 (812 - 1375) | 13.452 (10.557 - 16.622) | -1.285 (-1.6to-0.969) | -2.098  ( -2.480 to -1.715 ) |
|  | Yemen | 1096 (704 - 1530) | 16.164 (10.659 - 22.827) | 2050 (1402 - 2827) | 10.349 (7.112 - 14.086) | -1.695 (-1.803to-1.586) | -1.396  ( -1.611 to -1.181 ) |
| **South Asia** | Bangladesh | 37674 (26915 - 47400) | 57.006 (41.282 - 71.378) | 47898 (30340 - 71857) | 30.804 (19.67 - 46.118) | -1.979 (-2.072to-1.887) | -1.920  ( -2.171 to -1.668 ) |
|  | Bhutan | 181 (116 - 261) | 48.774 (31.962 - 69.829) | 202 (126 - 323) | 28.783 (18.306 - 45.925) | -1.888 (-2.023to-1.754) | -1.704  ( -1.850 to -1.558 ) |
|  | India | 295498 (251035 - 341652) | 46.739 (39.723 - 54.226) | 431223 (376460 - 488651) | 31.619 (27.685 - 35.798) | -1.46 (-1.685to-1.233) | -1.248  ( -1.411 to -1.084 ) |
|  | Nepal | 5965 (4327 - 8086) | 45.557 (33.19 - 61.743) | 8708 (6196 - 12044) | 32.087 (22.946 - 44.236) | -1.019 (-1.301to-0.737) | -1.110  ( -1.208 to -1.013 ) |
|  | Pakistan | 33886 (28459 - 40754) | 46.232 (38.903 - 56.055) | 79876 (63047 - 100968) | 44.798 (35.257 - 56.359) | -0.374 (-0.549to-0.199) | -0.067  ( -0.166 - 0.032 ) |
| **East Asia** | China | 1359140 (1149643 - 1576295) | 134.121 (113.494 - 155.503) | 982657 (797644 - 1210379) | 48.674 (39.591 - 59.482) | -3.917 (-4.25to-3.584) | -3.247  ( -3.353 to -3.141 ) |
|  | Democratic People's Republic of Korea | 13150 (9169 - 17574) | 66.898 (46.54 - 88.948) | 17042 (12328 - 22964) | 49.996 (36.475 - 67.01) | -1.027 (-1.068to-0.986) | -0.938  ( -0.983 to -0.893 ) |
|  | Taiwan  (Province of China) | 31551 (28408 - 34427) | 168.833 (152.478 - 184.22) | 24948 (19941 - 30799) | 66.677 (53.222 - 82.498) | -3.411 (-3.565to-3.257) | -2.978  ( -3.353 to -2.602 ) |
| **Oceania** | American Samoa | 12 (10 - 15) | 42.483 (34.11 - 51.976) | 25 (19 - 31) | 45.033 (35.816 - 56.695) | 0.636 (0.455-0.817) | 0.198  ( -0.283 - 0.682 ) |
|  | Cook Islands | 1 (1 - 1) | 6.355 (4.975 - 7.869) | 1 (1 - 2) | 4.97 (3.739 - 6.354) | -0.596 (-0.785to-0.406) | -0.786  ( -1.084 to -0.487 ) |
|  | Fiji | 57 (46 - 70) | 11.657 (9.324 - 14.513) | 85 (61 - 116) | 9.407 (6.874 - 12.762) | -0.704 (-0.909to-0.499) | -0.699  ( -0.954 to -0.443 ) |
|  | Guam | 70 (63 - 78) | 72.067 (64.181 - 79.565) | 91 (78 - 105) | 45.883 (39.344 - 53.108) | -1.072 (-1.297to-0.846) | -1.540  ( -1.892 to -1.186 ) |
|  | Kiribati | 13 (10 - 16) | 27.601 (21.064 - 34.51) | 25 (17 - 34) | 25.981 (18.715 - 36.146) | -0.111 (-0.164to-0.058) | -0.188  ( -0.229 to -0.148 ) |
|  | Marshall Islands | 8 (6 - 10) | 38.863 (28.847 - 49.784) | 16 (11 - 22) | 34.089 (24.241 - 45.364) | -0.462 (-0.536to-0.388) | -0.426  ( -0.472 to -0.380 ) |
|  | Micronesia  (Federated States of) | 25 (18 - 32) | 43.987 (32.043 - 57.073) | 29 (22 - 40) | 31.96 (23.795 - 43.142) | -1.091 (-1.198to-0.985) | -1.033  ( -1.096 to -0.971 ) |
|  | Nauru | 3 (2 - 4) | 48.026 (31.76 - 67.646) | 3 (2 - 4) | 37.325 (23.678 - 55.826) | -0.859 (-1.008to-0.71) | -0.817  ( -0.872 to -0.762 ) |
|  | Niue | 1 (0 - 1) | 31.152 (22.794 - 40.815) | 1 (0 - 1) | 24.837 (18.667 - 32.219) | -1.07 (-1.19to-0.95) | -0.712  ( -0.800 to -0.624 ) |
|  | Northern Mariana Islands | 14 (10 - 18) | 49.203 (38.853 - 62.818) | 27 (22 - 32) | 42.615 (35.316 - 49.99) | -0.184 (-0.376-0.01) | -0.434  ( -1.104 - 0.241 ) |
|  | Palau | 0 (0 - 0) | 1.691 (1.113 - 2.525) | 0 (0 - 0) | 1.195 (0.842 - 1.658) | -0.898 (-1.061to-0.735) | -1.107  ( -1.271 to -0.942 ) |
|  | Papua New Guinea | 718 (422 - 1098) | 29.86 (17.728 - 45.515) | 1691 (1016 - 2703) | 24.256 (14.623 - 38.624) | -0.648 (-0.752to-0.544) | -0.640  ( -0.704 to -0.576 ) |
|  | Samoa | 63 (49 - 81) | 57.671 (45.338 - 73.273) | 89 (67 - 118) | 51.398 (39.093 - 67.999) | -0.369 (-0.471to-0.267) | -0.373  ( -0.405 to -0.341 ) |
|  | Solomon Islands | 66 (36 - 97) | 37.379 (20.727 - 54.063) | 152 (109 - 208) | 32.557 (23.146 - 44.305) | -0.376 (-0.502to-0.251) | -0.462  ( -0.604 to -0.319 ) |
|  | Tokelau | 0 (0 - 1) | 34.052 (24.002 - 46.488) | 0 (0 - 0) | 24.791 (17.038 - 34.526) | -1.234 (-1.336to-1.133) | -0.974  ( -1.055 to -0.894 ) |
|  | Tonga | 15 (11 - 21) | 24.491 (17.6 - 33.818) | 18 (12 - 25) | 20.564 (13.813 - 29.437) | -0.559 (-0.613to-0.506) | -0.557  ( -0.706 to -0.408 ) |
|  | Tuvalu | 3 (2 - 4) | 37.71 (27.248 - 48.357) | 3 (3 - 4) | 28.279 (22.644 - 35.599) | -0.908 (-0.983to-0.833) | -0.921  ( -0.968 to -0.873 ) |
|  | Vanuatu | 27 (19 - 37) | 32.721 (22.969 - 45.248) | 62 (45 - 81) | 27.628 (20.448 - 36.194) | -0.668 (-0.791to-0.545) | -0.518  ( -0.771 to -0.264 ) |
| **Southeast Asia** | Cambodia | 4045 (3026 - 5109) | 68.355 (51.274 - 86.543) | 8868 (6484 - 11849) | 60.291 (44.064 - 80.876) | -0.553 (-0.668to-0.438) | -0.368  ( -0.461 to -0.275 ) |
|  | Indonesia | 62125 (49206 - 77402) | 47.397 (37.535 - 59.016) | 110166 (77572 - 150123) | 38.404 (27.458 - 51.324) | -0.752 (-0.83to-0.673) | -0.666  ( -0.729 to -0.603 ) |
|  | Lao People's Democratic Republic | 1948 (1243 - 2682) | 74.721 (48.021 - 101.775) | 2840 (2000 - 3905) | 48.174 (34.417 - 65.56) | -1.587 (-1.667to-1.507) | -1.406  ( -1.452 to -1.360 ) |
|  | Malaysia | 26831 (22846 - 31545) | 219.481 (186.088 - 256.919) | 50755 (42963 - 60013) | 158.226 (134.379 - 187.142) | -1.465 (-1.83to-1.099) | -1.028  ( -1.314 to -0.741 ) |
|  | Maldives | 18 (11 - 24) | 15.18 (9.619 - 19.807) | 26 (20 - 33) | 5.609 (4.333 - 7.224) | -3.593 (-3.737to-3.448) | -3.257  ( -3.449 to -3.064 ) |
|  | Mauritius | 202 (184 - 223) | 23.574 (21.477 - 25.999) | 392 (341 - 443) | 22.122 (19.249 - 24.999) | -0.468 (-0.713to-0.223) | -0.147  ( -0.816 - 0.527 ) |
|  | Myanmar | 18715 (13244 - 24458) | 64.39 (46.589 - 84.282) | 21398 (15943 - 28155) | 39.015 (29.339 - 50.742) | -1.925 (-2.039to-1.811) | -1.627  ( -1.722 to -1.531 ) |
|  | Philippines | 20634 (18465 - 23369) | 50.758 (44.696 - 58.321) | 47336 (39184 - 56739) | 47.83 (39.657 - 57.22) | -0.18 (-0.262to-0.098) | -0.182  ( -0.238 to -0.125 ) |
|  | Seychelles | 29 (24 - 34) | 50.758 (42.538 - 59.799) | 56 (46 - 68) | 43.777 (35.818 - 53.078) | -0.422 (-0.709to-0.135) | -0.399  ( -0.597 to -0.200 ) |
|  | Sri Lanka | 4062 (3394 - 4918) | 30.764 (25.576 - 36.898) | 5049 (3271 - 7246) | 18.804 (12.212 - 26.754) | -1.735 (-2.019to-1.45) | -1.418  ( -1.826 to -1.009 ) |
|  | Thailand | 25397 (21509 - 30029) | 55.667 (47.058 - 64.935) | 40553 (30858 - 51858) | 41.3 (31.904 - 52.361) | -1.502 (-1.732to-1.271) | -0.947  ( -1.102 to -0.793 ) |
|  | Timor-Leste | 200 (132 - 302) | 46.16 (30.638 - 69.437) | 386 (275 - 541) | 39.848 (28.311 - 55.684) | -0.514 (-0.743to-0.284) | -0.512  ( -0.865 to -0.157 ) |
|  | Viet Nam | 41926 (31907 - 54596) | 90.992 (68.516 - 118.214) | 88504 (64537 - 116712) | 79.217 (58.336 - 103.274) | -0.357 (-0.447to-0.267) | -0.434  ( -0.509 to -0.359 ) |
| **Central Sub-Saharan Africa** | Angola | 774 (557 - 1053) | 13.836 (9.997 - 18.795) | 2069 (1436 - 2877) | 11.939 (8.343 - 16.598) | -0.445 (-0.513to-0.376) | -0.477  ( -0.936 to -0.016 ) |
|  | Central African Republic | 266 (195 - 358) | 16.958 (12.482 - 22.262) | 468 (318 - 690) | 14.196 (9.845 - 20.498) | -0.701 (-0.771to-0.632) | -0.573  ( -0.734 to -0.411 ) |
|  | Congo | 225 (172 - 291) | 16.536 (12.725 - 21.306) | 492 (351 - 678) | 12.885 (9.404 - 17.177) | -1.019 (-1.16to-0.877) | -0.788  ( -1.102 to -0.474 ) |
|  | Democratic Republic of the Congo | 2529 (1824 - 3412) | 12.034 (8.621 - 16.47) | 5724 (3893 - 8627) | 11.075 (7.624 - 16.815) | -0.248 (-0.354to-0.143) | -0.288  ( -0.428 to -0.148 ) |
|  | Equatorial Guinea | 37 (27 - 51) | 14.901 (10.741 - 20.158) | 79 (47 - 126) | 9.875 (6.046 - 15.482) | -1.573 (-1.832to-1.313) | -1.261  ( -1.465 to -1.058 ) |
|  | Gabon | 100 (81 - 121) | 15.477 (12.426 - 18.677) | 158 (113 - 219) | 11.864 (8.511 - 16.093) | -1.021 (-1.137to-0.904) | -0.843  ( -1.024 to -0.662 ) |
| **Eastern Sub-Saharan Africa** | Burundi | 2145 (1565 - 2850) | 67.751 (48.885 - 89.811) | 3600 (2356 - 5297) | 46.557 (30.819 - 68.359) | -1.569 (-1.733to-1.404) | -1.221  ( -1.411 to -1.030 ) |
|  | Comoros | 137 (79 - 202) | 48.87 (29.979 - 72.072) | 255 (158 - 392) | 40.835 (25.521 - 62.261) | -0.894 (-1.13to-0.657) | -0.658  ( -1.513 - 0.205 ) |
|  | Djibouti | 110 (65 - 171) | 47.176 (28.618 - 72.496) | 413 (220 - 689) | 42.577 (23.908 - 69.493) | -0.439 (-0.593to-0.285) | -0.304  ( -0.423 to -0.185 ) |
|  | Eritrea | 1182 (943 - 1480) | 61.304 (49.057 - 76.743) | 2343 (1624 - 3343) | 52.612 (37.707 - 74.022) | -0.521 (-0.558to-0.484) | -0.516  ( -0.688 to -0.344 ) |
|  | Ethiopia | 19732 (12880 - 25561) | 67.517 (44.543 - 86.146) | 27805 (18898 - 43138) | 42.073 (28.434 - 64.527) | -1.864 (-2.032to-1.697) | -1.518  ( -1.575 to -1.461 ) |
|  | Kenya | 6785 (4825 - 9401) | 55.807 (39.652 - 78.702) | 20534 (14941 - 27785) | 61.148 (44.731 - 82.516) | 0.578 (0.39-0.766) | 0.302  ( 0.176 - 0.427 ) |
|  | Madagascar | 3231 (2519 - 4095) | 45.638 (34.582 - 58.976) | 6690 (4311 - 9880) | 35.947 (23.105 - 52.901) | -0.799 (-0.896to-0.703) | -0.776  ( -0.845 to -0.706 ) |
|  | Malawi | 666 (503 - 847) | 11.572 (8.883 - 14.646) | 1270 (883 - 1819) | 10.799 (7.766 - 15.201) | -0.443 (-0.568to-0.318) | -0.210  ( -0.304 to -0.116 ) |
|  | Mozambique | 205 (150 - 269) | 2.749 (2.045 - 3.563) | 492 (352 - 654) | 3.224 (2.371 - 4.216) | 0.924 (0.783-1.066) | 0.527  ( 0.437 - 0.617 ) |
|  | Rwanda | 3237 (2537 - 4046) | 78.388 (59.759 - 97.959) | 4163 (2758 - 6023) | 45.695 (30.614 - 66.294) | -2.537 (-2.818to-2.256) | -1.702  ( -1.876 to -1.528 ) |
|  | Somalia | 2302 (1563 - 3238) | 55.86 (38.242 - 77.999) | 5398 (3457 - 8486) | 50.282 (32.926 - 78.431) | -0.38 (-0.437to-0.323) | -0.342  ( -0.417 to -0.268 ) |
|  | South Sudan | 1464 (990 - 2179) | 44.393 (29.533 - 66.55) | 2585 (1611 - 4064) | 42.51 (26.826 - 67.094) | -0.298 (-0.519to-0.076) | -0.104  ( -0.246 - 0.039 ) |
|  | Uganda | 9010 (6761 - 11960) | 99.354 (74.789 - 130.778) | 21765 (15325 - 30100) | 89.548 (64.142 - 124.616) | -1.073 (-1.382to-0.763) | -0.378  ( -0.499 to -0.257 ) |
|  | United Republic of Tanzania | 7971 (5873 - 10579) | 53.292 (38.871 - 70.503) | 15579 (10263 - 22788) | 41.869 (28.136 - 60.703) | -0.881 (-0.931to-0.832) | -0.786  ( -0.899 to -0.672 ) |
|  | Zambia | 2333 (1854 - 2941) | 53.92 (42.599 - 68.214) | 8545 (3435 - 15135) | 74.084 (30.201 - 130.105) | 1.251 (0.964-1.538) | 1.070  ( 0.911 - 1.230 ) |
| **Southern Sub-Saharan Africa** | Botswana | 154 (106 - 225) | 21.253 (14.907 - 30.544) | 314 (187 - 481) | 16.789 (10.315 - 24.996) | -0.916 (-1.158to-0.674) | -0.746  ( -1.122 to -0.369 ) |
|  | Eswatini | 83 (64 - 108) | 21.452 (16.351 - 27.606) | 186 (124 - 259) | 24.689 (16.712 - 34.232) | 0.655 (0.17-1.142) | 0.433  ( 0.250 - 0.616 ) |
|  | Lesotho | 160 (117 - 232) | 16.556 (12.054 - 23.761) | 365 (262 - 503) | 27.637 (19.829 - 38.047) | 2.227 (1.812-2.644) | 1.718  ( 1.464 - 1.973 ) |
|  | Namibia | 175 (130 - 231) | 20.078 (14.975 - 26.444) | 330 (226 - 452) | 17.716 (12.333 - 23.786) | -0.772 (-1.135to-0.408) | -0.358  ( -0.578 to -0.137 ) |
|  | South Africa | 4322 (3679 - 5081) | 16.996 (14.275 - 20.172) | 7666 (6738 - 8536) | 14.612 (12.893 - 16.244) | -0.674 (-0.906to-0.441) | -0.509  ( -0.761 to -0.258 ) |
|  | Zimbabwe | 928 (735 - 1182) | 16.928 (13.56 - 21.345) | 2728 (1961 - 3568) | 26.199 (19.265 - 34.247) | 2.001 (1.482-2.523) | 1.384  ( 0.793 - 1.978 ) |
| **Western Sub-Saharan Africa** | Benin | 119 (90 - 151) | 4.523 (3.45 - 5.739) | 388 (248 - 572) | 4.716 (3.019 - 6.873) | 0.034 (-0.091-0.159) | 0.123  ( -0.091 - 0.338 ) |
|  | Burkina Faso | 286 (208 - 373) | 5.086 (3.73 - 6.67) | 788 (534 - 1082) | 5.708 (3.861 - 7.897) | 0.355 (0.215-0.495) | 0.386  ( 0.161 - 0.611 ) |
|  | Cabo Verde | 3 (2 - 3) | 1.304 (1.052 - 1.603) | 42 (32 - 57) | 7.609 (5.651 - 10.118) | 4.577 (3.024-6.154) | 5.905  ( 5.626 - 6.185 ) |
|  | Cameroon | 340 (259 - 434) | 5.343 (4.067 - 6.804) | 1262 (824 - 1800) | 6.063 (3.93 - 8.516) | 0.35 (0.2-0.499) | 0.381  ( 0.306 - 0.455 ) |
|  | Chad | 141 (102 - 190) | 3.999 (2.905 - 5.346) | 549 (375 - 777) | 5.843 (3.972 - 8.218) | 1.269 (1.068-1.471) | 1.289  ( 1.104 - 1.475 ) |
|  | Côte d'Ivoire | 766 (559 - 999) | 11.54 (8.504 - 15.183) | 2008 (1280 - 2926) | 11.068 (7.2 - 16.12) | -0.241 (-0.376to-0.106) | -0.132  ( -0.367 - 0.105 ) |
|  | Gambia | 29 (22 - 37) | 5.372 (3.983 - 6.894) | 90 (64 - 120) | 6.059 (4.377 - 8.015) | 0.118 (-0.075-0.312) | 0.370  ( -0.796 - 1.550 ) |
|  | Ghana | 307 (217 - 413) | 3.15 (2.21 - 4.283) | 123 (84 - 179) | 0.456 (0.317 - 0.663) | -8.773 (-10.367to-7.151) | -5.921  ( -6.493 to -5.344 ) |
|  | Guinea | 387 (304 - 490) | 9.586 (7.518 - 12.128) | 830 (586 - 1165) | 10.212 (7.242 - 14.217) | 0.325 (0.243-0.407) | 0.209  ( 0.106 - 0.312 ) |
|  | Guinea-Bissau | 42 (30 - 58) | 7.14 (5.083 - 9.915) | 104 (74 - 142) | 7.944 (5.681 - 10.794) | 0.405 (0.323-0.487) | 0.382  ( 0.261 - 0.502 ) |
|  | Liberia | 70 (49 - 96) | 4.725 (3.301 - 6.542) | 214 (134 - 334) | 5.41 (3.416 - 8.497) | 0.412 (0.262-0.562) | 0.457  ( 0.027 - 0.888 ) |
|  | Mali | 325 (264 - 399) | 5.933 (4.858 - 7.358) | 700 (483 - 978) | 5.218 (3.635 - 7.394) | -0.324 (-0.482to-0.166) | -0.400  ( -0.518 to -0.282 ) |
|  | Mauritania | 58 (42 - 80) | 4.488 (3.272 - 6.134) | 118 (66 - 194) | 4.033 (2.248 - 6.626) | -0.5 (-0.563to-0.437) | -0.371  ( -0.521 to -0.221 ) |
|  | Niger | 188 (127 - 268) | 4.229 (2.827 - 6.03) | 550 (304 - 927) | 4.228 (2.363 - 7.089) | -0.035 (-0.136-0.065) | 0.023  ( -0.113 - 0.160 ) |
|  | Nigeria | 20436 (15541 - 25860) | 36.135 (27.745 - 45.74) | 43116 (28166 - 59119) | 31.312 (20.986 - 42.471) | -0.537 (-0.649to-0.424) | -0.484  ( -0.573 to -0.394 ) |
|  | Sao Tome and Principe | 1 (0 - 1) | 0.87 (0.621 - 1.168) | 1 (1 - 2) | 0.827 (0.549 - 1.193) | -0.478 (-0.741to-0.214) | -0.187  ( -0.375 - 0.000 ) |
|  | Senegal | 210 (146 - 291) | 4.667 (3.276 - 6.453) | 571 (340 - 861) | 5.156 (3.074 - 7.778) | 0.421 (0.317-0.526) | 0.414  ( 0.308 - 0.519 ) |
|  | Sierra Leone | 116 (82 - 151) | 4.487 (3.231 - 5.901) | 313 (200 - 447) | 5.297 (3.408 - 7.561) | 0.726 (0.65-0.803) | 0.540  ( 0.297 - 0.783 ) |
|  | Togo | 97 (73 - 125) | 4.868 (3.693 - 6.3) | 368 (249 - 523) | 5.94 (4.049 - 8.363) | 0.649 (0.551-0.747) | 0.666  ( 0.523 - 0.809 ) |

***Abbreviations:*** *ASR, age-standardized rate; EAPC, estimated annual percentage change; AAPC, average annual percent change.*

**Table S2. Average annual percent change (AAPC) estimates derived from joinpoint regression using group comparison across the five SDI regions, stratified by sex**

| **SDI regions** | **Sex** | **Measure** | **AAPC (95%CI)** | **P-Value** |
| --- | --- | --- | --- | --- |
| High SDI | Female | Incidence | -0.868 (-0.978 to -0.759) | 0.000444 |
| High SDI | Male | Incidence | -0.718 (-0.793 to -0.643) |  |
| High-middle SDI | Female | Incidence | -0.419 (-0.786 to -0.05) | 0.008 |
| High-middle SDI | Male | Incidence | -0.112 (-0.454 to 0.23) |  |
| Low SDI | Female | Incidence | -1.178 (-1.272 to -1.084) | 0.000222 |
| Low SDI | Male | Incidence | -0.66 (-0.742 to -0.578) |  |
| Low-middle SDI | Female | Incidence | -0.984 (-1.161 to -0.807) | 0.000222 |
| Low-middle SDI | Male | Incidence | -0.414 (-0.501 to -0.327) |  |
| Middle SDI | Female | Incidence | -2.502 (-2.726 to -2.277) | 0.000222 |
| Middle SDI | Male | Incidence | -1.145 (-1.231 to -1.058) |  |
| High SDI | Female | Deaths | -1.882 (-1.956 to -1.809) | 0.000222 |
| High SDI | Male | Deaths | -1.649 (-1.738 to -1.561) |  |
| High-middle SDI | Female | Deaths | -2.464 (-2.726 to -2.202) | 0.000222 |
| High-middle SDI | Male | Deaths | -2.241 (-2.478 to -2.004) |  |
| Low SDI | Female | Deaths | -1.199 (-1.3 to -1.097) | 0.000222 |
| Low SDI | Male | Deaths | -0.686 (-0.771 to -0.601) |  |
| Low-middle SDI | Female | Deaths | -1.107 (-1.29 to -0.923) | 0.000222 |
| Low-middle SDI | Male | Deaths | -0.5 (-0.59 to -0.41) |  |
| Middle SDI | Female | Deaths | -3.462 (-3.681 to -3.242) | 0.000222 |
| Middle SDI | Male | Deaths | -2.22 (-2.296 to -2.143) |  |
| High SDI | Female | DALYs | -2.034 (-2.106 to -1.962) | 0.000222 |
| High SDI | Male | DALYs | -1.897 (-1.973 to -1.82) |  |
| High-middle SDI | Female | DALYs | -2.617 (-2.899 to -2.335) | 0.000222 |
| High-middle SDI | Male | DALYs | -2.304 (-2.573 to -2.034) |  |
| Low SDI | Female | DALYs | -1.376 (-1.471 to -1.281) | 0.000222 |
| Low SDI | Male | DALYs | -0.785 (-0.863 to -0.707) |  |
| Low-middle SDI | Female | DALYs | -1.22 (-1.398 to -1.041) | 0.000222 |
| Low-middle SDI | Male | DALYs | -0.576 (-0.655 to -0.498) |  |
| Middle SDI | Female | DALYs | -3.644 (-3.861 to -3.426) | 0.000222 |
| Middle SDI | Male | DALYs | -2.356 (-2.437 to -2.274) |  |
| ***Abbreviations:*** *AAPC,average annual percent change; SDI, Socio-demographic index. Joinpoint regression was performed with a maximum of 2 joinpoints by group comparison* | | | | |

**Table S3. Age-standardised incidence, mortality, and disability-adjusted life years (DALY) rates across SDI regions, 1990–2023, with annual percentage changes (APC) by sex**

| **Location** | **Measure** | **Segment** | **Both** | | **Female** | | **Male** | |
| --- | --- | --- | --- | --- | --- | --- | --- | --- |
|  |  |  | **APC  (year range, 95% CI)** | **P.Value** | **APC  (year range, 95% CI)** | **P.Value** | **APC  (year range, 95% CI)** | **P.Value** |
| Global | Incidence | 0 | (1990-1993)  0.19 (-0.36 to 0.743) | 0.474 | (1990-1994)  -0.722 (-1.14 to -0.303) | 0.002 | (1990-1995)  -0.046 (-0.295 to 0.204) | 0.7 |
|  |  | 1 | (1993-1999)  -1.168 (-1.368 to -0.968) | <0.001 | (1994-2000)  -1.964 (-2.178 to -1.749) | <0.001 | (1995-1999)  -1.205 (-1.666 to -0.743) | <0.001 |
|  |  | 2 | (1999-2004)  -3.563 (-3.789 to -3.336) | <0.001 | (2000-2004)  -4.262 (-4.708 to -3.814) | <0.001 | (1999-2004)  -3.348 (-3.586 to -3.11) | <0.001 |
|  |  | 3 | (2004-2009)  -0.772 (-0.984 to -0.56) | <0.001 | (2004-2013)  -1.234 (-1.324 to -1.144) | <0.001 | (2004-2007)  -0.725 (-1.396 to -0.05) | 0.037 |
|  |  | 4 | (2009-2013)  -0.193 (-0.586 to 0.202) | 0.314 | (2013-2021)  0.495 (0.353 to 0.636) | <0.001 | (2007-2012)  -0.123 (-0.356 to 0.111) | 0.28 |
|  |  | 5 | (2013-2021)  0.826 (0.693 to 0.959) | <0.001 |  |  | (2012-2021)  0.884 (0.776 to 0.993) | <0.001 |
|  | Deaths | 0 | (1990-1995)  -0.923 (-1.123 to -0.723) | <0.001 | (1990-1993)  -1.141 (-1.809 to -0.469) | 0.003 | (1990-1995)  -0.556 (-0.817 to -0.295) | <0.001 |
|  |  | 1 | (1995-2000)  -2.296 (-2.529 to -2.062) | <0.001 | (1993-2000)  -2.521 (-2.691 to -2.35) | <0.001 | (1995-2000)  -2.124 (-2.417 to -1.831) | <0.001 |
|  |  | 2 | (2000-2004)  -4.647 (-4.956 to -4.337) | <0.001 | (2000-2004)  -5.062 (-5.5 to -4.622) | <0.001 | (2000-2003)  -4.616 (-5.389 to -3.838) | <0.001 |
|  |  | 3 | (2004-2007)  -2.946 (-3.526 to -2.361) | <0.001 | (2004-2007)  -3.583 (-4.351 to -2.809) | <0.001 | (2003-2006)  -3.574 (-4.245 to -2.898) | <0.001 |
|  |  | 4 | (2007-2014)  -1.395 (-1.501 to -1.288) | <0.001 | (2007-2013)  -2.123 (-2.313 to -1.934) | <0.001 | (2006-2014)  -1.227 (-1.326 to -1.127) | <0.001 |
|  |  | 5 | (2014-2021)  -0.312 (-0.435 to -0.188) | <0.001 | (2013-2021)  -0.296 (-0.414 to -0.177) | <0.001 | (2014-2021)  -0.385 (-0.538 to -0.231) | <0.001 |
|  | DALYs | 0 | (1990-1994)  -0.894 (-1.118 to -0.67) | <0.001 | (1990-1993)  -1.244 (-1.875 to -0.609) | 0.001 | (1990-1995)  -0.857 (-1.116 to -0.597) | <0.001 |
|  |  | 1 | (1994-2000)  -2.314 (-2.43 to -2.198) | <0.001 | (1993-2000)  -2.637 (-2.794 to -2.478) | <0.001 | (1995-2000)  -2.279 (-2.561 to -1.997) | <0.001 |
|  |  | 2 | (2000-2003)  -4.926 (-5.387 to -4.463) | <0.001 | (2000-2004)  -5.275 (-5.679 to -4.87) | <0.001 | (2000-2003)  -4.657 (-5.44 to -3.869) | <0.001 |
|  |  | 3 | (2003-2006)  -3.781 (-4.206 to -3.354) | <0.001 | (2004-2007)  -3.567 (-4.291 to -2.837) | <0.001 | (2003-2006)  -3.618 (-4.3 to -2.932) | <0.001 |
|  |  | 4 | (2006-2013)  -1.716 (-1.795 to -1.636) | <0.001 | (2007-2013)  -2.218 (-2.4 to -2.036) | <0.001 | (2006-2014)  -1.376 (-1.478 to -1.275) | <0.001 |
|  |  | 5 | (2013-2021)  -0.415 (-0.491 to -0.34) | <0.001 | (2013-2021)  -0.415 (-0.534 to -0.296) | <0.001 | (2014-2021)  -0.302 (-0.464 to -0.14) | 0.001 |
| Low SDI | Incidence | 0 | (1990-1997)  -0.295 (-0.448 to -0.143) | 0.001 | (1990-1994)  -0.023 (-0.298 to 0.253) | 0.861 | (1990-1997)  -0.229 (-0.387 to -0.069) | 0.007 |
|  |  | 1 | (1997-2004)  -2.627 (-2.812 to -2.443) | <0.001 | (1994-1998)  -1.155 (-1.557 to -0.751) | <0.001 | (1997-2004)  -2.28 (-2.475 to -2.084) | <0.001 |
|  |  | 2 | (2004-2011)  -0.966 (-1.17 to -0.761) | <0.001 | (1998-2005)  -3.105 (-3.233 to -2.978) | <0.001 | (2004-2012)  -0.436 (-0.608 to -0.264) | <0.001 |
|  |  | 3 | (2011-2015)  0.453 (-0.153 to 1.061) | 0.134 | (2005-2011)  -1.543 (-1.742 to -1.344) | <0.001 | (2012-2015)  0.622 (-0.692 to 1.954) | 0.335 |
|  |  | 4 | (2015-2021)  -0.321 (-0.531 to -0.11) | 0.005 | (2011-2014)  0.784 (-0.171 to 1.748) | 0.101 | (2015-2021)  -0.205 (-0.438 to 0.029) | 0.082 |
|  |  | 5 |  |  | (2014-2021)  -0.387 (-0.519 to -0.254) | <0.001 |  |  |
|  | Deaths | 0 | (1990-1997)  -0.291 (-0.45 to -0.132) | 0.001 | (1990-1994)  -0.102 (-0.397 to 0.194) | 0.474 | (1990-1997)  -0.217 (-0.38 to -0.054) | 0.012 |
|  |  | 1 | (1997-2004)  -2.636 (-2.829 to -2.442) | <0.001 | (1994-1997)  -0.692 (-1.529 to 0.152) | 0.101 | (1997-2004)  -2.288 (-2.488 to -2.087) | <0.001 |
|  |  | 2 | (2004-2011)  -1.001 (-1.215 to -0.787) | <0.001 | (1997-2005)  -2.987 (-3.095 to -2.878) | <0.001 | (2004-2012)  -0.471 (-0.648 to -0.294) | <0.001 |
|  |  | 3 | (2011-2015)  0.453 (-0.178 to 1.088) | 0.149 | (2005-2011)  -1.642 (-1.854 to -1.431) | <0.001 | (2012-2015)  0.633 (-0.709 to 1.994) | 0.337 |
|  |  | 4 | (2015-2021)  -0.491 (-0.709 to -0.273) | <0.001 | (2011-2014)  0.874 (-0.13 to 1.888) | 0.083 | (2015-2021)  -0.355 (-0.59 to -0.119) | 0.005 |
|  |  | 5 |  |  | (2014-2021)  -0.579 (-0.718 to -0.44) | <0.001 |  |  |
|  | DALYs | 0 | (1990-1994)  -0.005 (-0.26 to 0.251) | 0.969 | (1990-1994)  -0.096 (-0.32 to 0.128) | 0.374 | (1990-1997)  -0.242 (-0.424 to -0.06) | 0.012 |
|  |  | 1 | (1994-1998)  -0.936 (-1.314 to -0.557) | <0.001 | (1994-1998)  -0.962 (-1.3 to -0.623) | <0.001 | (1997-2004)  -2.322 (-2.545 to -2.099) | <0.001 |
|  |  | 2 | (1998-2004)  -2.801 (-2.973 to -2.628) | <0.001 | (1998-2004)  -3.396 (-3.535 to -3.256) | <0.001 | (2004-2011)  -0.62 (-0.868 to -0.372) | <0.001 |
|  |  | 3 | (2004-2011)  -1.192 (-1.334 to -1.049) | <0.001 | (2004-2011)  -2.037 (-2.159 to -1.915) | <0.001 | (2011-2021)  -0.122 (-0.25 to 0.006) | 0.061 |
|  |  | 4 | (2011-2015)  0.117 (-0.316 to 0.551) | 0.575 | (2011-2014)  0.402 (-0.411 to 1.221) | 0.31 |  |  |
|  |  | 5 | (2015-2021)  -0.484 (-0.639 to -0.329) | <0.001 | (2014-2021)  -0.628 (-0.742 to -0.513) | <0.001 |  |  |
| Low-middle SDI | Incidence | 0 | (1990-1997)  -0.495 (-0.694 to -0.295) | <0.001 | (1990-1998)  -0.859 (-1.022 to -0.695) | <0.001 | (1990-1997)  -0.333 (-0.589 to -0.077) | 0.013 |
|  |  | 1 | (1997-2004)  -2.067 (-2.286 to -1.847) | <0.001 | (1998-2004)  -2.894 (-3.19 to -2.598) | <0.001 | (1997-2004)  -1.647 (-1.93 to -1.364) | <0.001 |
|  |  | 2 | (2004-2012)  -0.295 (-0.453 to -0.137) | 0.001 | (2004-2012)  -0.804 (-0.979 to -0.629) | <0.001 | (2004-2021)  0.065 (0.01 to 0.12) | 0.023 |
|  |  | 3 | (2012-2018)  0.192 (-0.058 to 0.444) | 0.124 | (2012-2017)  0.696 (0.317 to 1.076) | 0.001 |  |  |
|  |  | 4 | (2018-2021)  -0.553 (-1.143 to 0.041) | 0.066 | (2017-2021)  -1.147 (-1.565 to -0.728) | <0.001 |  |  |
|  | Deaths | 0 | (1990-1997)  -0.471 (-0.681 to -0.261) | <0.001 | (1990-1998)  -0.857 (-1.016 to -0.697) | <0.001 | (1990-1997)  -0.306 (-0.571 to -0.04) | 0.026 |
|  |  | 1 | (1997-2004)  -2.136 (-2.367 to -1.903) | <0.001 | (1998-2004)  -2.978 (-3.269 to -2.686) | <0.001 | (1997-2004)  -1.705 (-1.995 to -1.414) | <0.001 |
|  |  | 2 | (2004-2012)  -0.443 (-0.609 to -0.278) | <0.001 | (2004-2012)  -0.972 (-1.145 to -0.8) | <0.001 | (2004-2021)  -0.079 (-0.136 to -0.022) | 0.008 |
|  |  | 3 | (2012-2018)  0.023 (-0.237 to 0.283) | 0.857 | (2012-2017)  0.485 (0.117 to 0.855) | 0.013 |  |  |
|  |  | 4 | (2018-2021)  -0.799 (-1.409 to -0.185) | 0.014 | (2017-2021)  -1.433 (-1.834 to -1.031) | <0.001 |  |  |
|  | DALYs | 0 | (1990-1998)  -0.714 (-0.869 to -0.56) | <0.001 | (1990-1998)  -0.91 (-1.086 to -0.733) | <0.001 | (1990-1997)  -0.406 (-0.621 to -0.191) | 0.001 |
|  |  | 1 | (1998-2004)  -2.395 (-2.677 to -2.112) | <0.001 | (1998-2004)  -3.11 (-3.427 to -2.792) | <0.001 | (1997-2004)  -1.805 (-2.04 to -1.569) | <0.001 |
|  |  | 2 | (2004-2008)  -0.248 (-0.836 to 0.344) | 0.386 | (2004-2012)  -1.116 (-1.303 to -0.929) | <0.001 | (2004-2007)  0.207 (-1.171 to 1.604) | 0.759 |
|  |  | 3 | (2008-2012)  -0.73 (-1.277 to -0.18) | 0.013 | (2012-2017)  0.389 (-0.034 to 0.813) | 0.069 | (2007-2021)  -0.226 (-0.288 to -0.164) | <0.001 |
|  |  | 4 | (2012-2018)  -0.034 (-0.287 to 0.219) | 0.777 | (2017-2021)  -1.625 (-2.094 to -1.153) | <0.001 |  |  |
|  |  | 5 | (2018-2021)  -0.966 (-1.573 to -0.356) | 0.004 |  |  |  |  |
| Middle SDI | Incidence | 0 | (1990-1993)  -0.875 (-1.306 to -0.442) | 0.001 | (1990-1993)  -1.82 (-2.853 to -0.777) | 0.002 | (1990-1993)  -0.284 (-1.006 to 0.444) | 0.418 |
|  |  | 1 | (1993-1999)  -2.253 (-2.408 to -2.099) | <0.001 | (1993-2000)  -3.458 (-3.707 to -3.208) | <0.001 | (1993-1999)  -1.701 (-1.95 to -1.452) | <0.001 |
|  |  | 2 | (1999-2004)  -4.759 (-4.928 to -4.589) | <0.001 | (2000-2004)  -5.933 (-6.559 to -5.302) | <0.001 | (1999-2003)  -4.435 (-4.841 to -4.028) | <0.001 |
|  |  | 3 | (2004-2007)  -2.634 (-3.119 to -2.146) | <0.001 | (2004-2007)  -3.52 (-4.625 to -2.403) | <0.001 | (2003-2006)  -3.15 (-3.939 to -2.353) | <0.001 |
|  |  | 4 | (2007-2016)  -0.11 (-0.176 to -0.043) | 0.003 | (2007-2015)  -1.273 (-1.437 to -1.11) | <0.001 | (2006-2009)  -0.183 (-0.971 to 0.612) | 0.63 |
|  |  | 5 | (2016-2021)  0.837 (0.602 to 1.072) | <0.001 | (2015-2021)  0.676 (0.35 to 1.002) | <0.001 | (2009-2021)  0.488 (0.414 to 0.561) | <0.001 |
|  | Deaths | 0 | (1990-1993)  -1.216 (-1.926 to -0.501) | 0.003 | (1990-1993)  -2.34 (-3.179 to -1.494) | <0.001 | (1990-1993)  -0.523 (-1.365 to 0.326) | 0.211 |
|  |  | 1 | (1993-1999)  -2.716 (-2.981 to -2.451) | <0.001 | (1993-2000)  -3.887 (-4.092 to -3.681) | <0.001 | (1993-1999)  -2.195 (-2.49 to -1.899) | <0.001 |
|  |  | 2 | (1999-2006)  -5.375 (-5.521 to -5.228) | <0.001 | (2000-2004)  -6.668 (-7.184 to -6.15) | <0.001 | (1999-2006)  -4.957 (-5.113 to -4.799) | <0.001 |
|  |  | 3 | (2006-2009)  -2.295 (-3.18 to -1.402) | <0.001 | (2004-2007)  -5.477 (-6.342 to -4.604) | <0.001 | (2006-2009)  -1.866 (-2.779 to -0.944) | <0.001 |
|  |  | 4 | (2009-2014)  -1.575 (-1.853 to -1.297) | <0.001 | (2007-2014)  -2.652 (-2.802 to -2.501) | <0.001 | (2009-2021)  -0.972 (-1.051 to -0.894) | <0.001 |
|  |  | 5 | (2014-2021)  -0.831 (-1.019 to -0.642) | <0.001 | (2014-2021)  -0.696 (-0.868 to -0.524) | <0.001 |  |  |
|  | DALYs | 0 | (1990-1992)  -0.873 (-2.126 to 0.395) | 0.162 | (1990-1993)  -2.562 (-3.343 to -1.774) | <0.001 | (1990-1993)  -0.989 (-1.612 to -0.361) | 0.004 |
|  |  | 1 | (1992-1999)  -2.92 (-3.091 to -2.747) | <0.001 | (1993-2000)  -4.186 (-4.376 to -3.997) | <0.001 | (1993-1999)  -2.45 (-2.663 to -2.236) | <0.001 |
|  |  | 2 | (1999-2006)  -5.454 (-5.579 to -5.328) | <0.001 | (2000-2004)  -6.852 (-7.319 to -6.382) | <0.001 | (1999-2006)  -4.996 (-5.113 to -4.879) | <0.001 |
|  |  | 3 | (2006-2009)  -2.458 (-3.176 to -1.734) | <0.001 | (2004-2007)  -5.498 (-6.297 to -4.692) | <0.001 | (2006-2009)  -1.942 (-2.605 to -1.275) | <0.001 |
|  |  | 4 | (2009-2014)  -1.665 (-1.907 to -1.422) | <0.001 | (2007-2014)  -2.804 (-2.943 to -2.665) | <0.001 | (2009-2014)  -1.201 (-1.428 to -0.973) | <0.001 |
|  |  | 5 | (2014-2021)  -0.843 (-1.006 to -0.681) | <0.001 | (2014-2021)  -0.81 (-0.969 to -0.65) | <0.001 | (2014-2021)  -0.82 (-0.969 to -0.67) | <0.001 |
| High-middle SDI | Incidence | 0 | (1990-1994)  0.848 (0.334 to 1.364) | 0.003 | (1990-1994)  0.391 (-0.546 to 1.337) | 0.393 | (1990-1994)  0.931 (0.376 to 1.489) | 0.002 |
|  |  | 1 | (1994-2000)  -0.976 (-1.264 to -0.686) | <0.001 | (1994-2000)  -0.838 (-1.382 to -0.292) | 0.005 | (1994-2000)  -1.087 (-1.4 to -0.773) | <0.001 |
|  |  | 2 | (2000-2004)  -4.06 (-4.604 to -3.514) | <0.001 | (2000-2003)  -4.415 (-6.407 to -2.38) | <0.001 | (2000-2004)  -4.21 (-4.775 to -3.641) | <0.001 |
|  |  | 3 | (2004-2007)  0.377 (-0.641 to 1.405) | 0.443 | (2003-2013)  -0.669 (-0.847 to -0.49) | <0.001 | (2004-2012)  0.087 (-0.061 to 0.235) | 0.232 |
|  |  | 4 | (2007-2012)  -0.303 (-0.637 to 0.032) | 0.073 | (2013-2021)  1.644 (1.303 to 1.986) | <0.001 | (2012-2021)  2.743 (2.565 to 2.922) | <0.001 |
|  |  | 5 | (2012-2021)  2.568 (2.398 to 2.738) | <0.001 |  |  |  |  |
|  | Deaths | 0 | (1990-1993)  0.205 (-0.374 to 0.788) | 0.463 | (1990-1994)  -1.175 (-1.99 to -0.353) | 0.008 | (1990-1993)  0.59 (-0.153 to 1.339) | 0.112 |
|  |  | 1 | (1993-2000)  -1.892 (-2.046 to -1.738) | <0.001 | (1994-2000)  -2.069 (-2.529 to -1.606) | <0.001 | (1993-2000)  -1.904 (-2.105 to -1.702) | <0.001 |
|  |  | 2 | (2000-2003)  -5.929 (-6.678 to -5.175) | <0.001 | (2000-2003)  -5.743 (-7.453 to -4.002) | <0.001 | (2000-2003)  -6.178 (-7.125 to -5.222) | <0.001 |
|  |  | 3 | (2003-2006)  -4.597 (-5.263 to -3.925) | <0.001 | (2003-2007)  -4.547 (-5.279 to -3.808) | <0.001 | (2003-2006)  -4.594 (-5.404 to -3.777) | <0.001 |
|  |  | 4 | (2006-2014)  -2.23 (-2.324 to -2.136) | <0.001 | (2007-2014)  -3.107 (-3.365 to -2.848) | <0.001 | (2006-2014)  -1.927 (-2.043 to -1.81) | <0.001 |
|  |  | 5 | (2014-2021)  0.04 (-0.128 to 0.207) | 0.621 | (2014-2021)  0.157 (-0.165 to 0.48) | 0.316 | (2014-2021)  -0.146 (-0.348 to 0.056) | 0.144 |
|  | DALYs | 0 | (1990-1993)  0.123 (-0.473 to 0.722) | 0.668 | (1990-2000)  -1.816 (-2.017 to -1.613) | <0.001 | (1990-1993)  0.418 (-0.105 to 0.943) | 0.109 |
|  |  | 1 | (1993-2000)  -2.037 (-2.196 to -1.877) | <0.001 | (2000-2003)  -6.428 (-8.328 to -4.489) | <0.001 | (1993-2000)  -2.076 (-2.216 to -1.937) | <0.001 |
|  |  | 2 | (2000-2003)  -6.055 (-6.826 to -5.277) | <0.001 | (2003-2007)  -4.855 (-5.684 to -4.02) | <0.001 | (2000-2003)  -6.079 (-6.752 to -5.402) | <0.001 |
|  |  | 3 | (2003-2006)  -4.869 (-5.558 to -4.174) | <0.001 | (2007-2010)  -2.887 (-4.456 to -1.293) | 0.002 | (2003-2006)  -4.779 (-5.357 to -4.199) | <0.001 |
|  |  | 4 | (2006-2014)  -2.459 (-2.554 to -2.364) | <0.001 | (2010-2013)  -4.264 (-5.897 to -2.603) | <0.001 | (2006-2014)  -2.153 (-2.237 to -2.07) | <0.001 |
|  |  | 5 | (2014-2021)  0.361 (0.191 to 0.531) | <0.001 | (2013-2021)  -0.213 (-0.497 to 0.071) | 0.131 | (2014-2021)  0.307 (0.164 to 0.45) | <0.001 |
| High SDI | Incidence | 0 | (1990-1997)  0.844 (0.675 to 1.012) | <0.001 | (1990-1997)  0.716 (0.528 to 0.903) | <0.001 | (1990-1997)  0.799 (0.614 to 0.983) | <0.001 |
|  |  | 1 | (1997-2003)  -1.419 (-1.693 to -1.144) | <0.001 | (1997-2004)  -1.29 (-1.527 to -1.052) | <0.001 | (1997-2003)  -1.51 (-1.786 to -1.233) | <0.001 |
|  |  | 2 | (2003-2007)  -0.445 (-1.033 to 0.146) | 0.131 | (2004-2007)  -0.27 (-1.64 to 1.12) | 0.683 | (2003-2006)  -0.43 (-1.591 to 0.745) | 0.453 |
|  |  | 3 | (2007-2013)  -1.479 (-1.762 to -1.196) | <0.001 | (2007-2013)  -1.998 (-2.325 to -1.671) | <0.001 | (2006-2021)  -1.24 (-1.314 to -1.166) | <0.001 |
|  |  | 4 | (2013-2021)  -1.016 (-1.231 to -0.802) | <0.001 | (2013-2016)  -0.543 (-2.234 to 1.178) | 0.509 |  |  |
|  |  | 5 |  |  | (2016-2021)  -1.593 (-2.088 to -1.096) | <0.001 |  |  |
|  | Deaths | 0 | (1990-1996)  -0.582 (-0.72 to -0.445) | <0.001 | (1990-1996)  -0.88 (-1.038 to -0.721) | <0.001 | (1990-1996)  -0.545 (-0.74 to -0.349) | <0.001 |
|  |  | 1 | (1996-2000)  -1.76 (-2.148 to -1.371) | <0.001 | (1996-2000)  -1.89 (-2.348 to -1.43) | <0.001 | (1996-2000)  -1.716 (-2.228 to -1.202) | <0.001 |
|  |  | 2 | (2000-2004)  -2.528 (-2.91 to -2.145) | <0.001 | (2000-2013)  -2.642 (-2.701 to -2.582) | <0.001 | (2000-2004)  -2.554 (-3.026 to -2.079) | <0.001 |
|  |  | 3 | (2004-2014)  -2.118 (-2.194 to -2.042) | <0.001 | (2013-2016)  -0.89 (-2.047 to 0.279) | 0.127 | (2004-2015)  -2.057 (-2.141 to -1.973) | <0.001 |
|  |  | 4 | (2014-2021)  -1.357 (-1.533 to -1.181) | <0.001 | (2016-2021)  -1.83 (-2.166 to -1.493) | <0.001 | (2015-2021)  -1.363 (-1.661 to -1.065) | <0.001 |
|  | DALYs | 0 | (1990-1995)  -0.56 (-0.765 to -0.355) | <0.001 | (1990-1996)  -1.019 (-1.206 to -0.832) | <0.001 | (1990-1995)  -0.529 (-0.759 to -0.298) | <0.001 |
|  |  | 1 | (1995-2000)  -1.988 (-2.25 to -1.725) | <0.001 | (1996-2000)  -2.161 (-2.697 to -1.622) | <0.001 | (1995-2000)  -2.02 (-2.308 to -1.731) | <0.001 |
|  |  | 2 | (2000-2003)  -2.949 (-3.759 to -2.133) | <0.001 | (2000-2013)  -2.756 (-2.817 to -2.695) | <0.001 | (2000-2003)  -3.008 (-3.879 to -2.13) | <0.001 |
|  |  | 3 | (2003-2013)  -2.381 (-2.458 to -2.304) | <0.001 | (2013-2016)  -1.149 (-2.369 to 0.087) | 0.066 | (2003-2014)  -2.325 (-2.395 to -2.256) | <0.001 |
|  |  | 4 | (2013-2021)  -1.603 (-1.753 to -1.453) | <0.001 | (2016-2021)  -1.975 (-2.347 to -1.602) | <0.001 | (2014-2021)  -1.569 (-1.771 to -1.365) | <0.001 |

***Abbreviations:*** *APC, annual percent change; DALY,disability-adjusted life years.*

**Table S4. Peak age group and corresponding incidence, deaths, and DALYs rates in 2021 for major regions**

| **Loation** | **Sex** | **Incidence*** | **Deaths*** | **DALYs*** |
| --- | --- | --- | --- | --- |
| **Global** | Both | 65-69  4.23 (3.66, 4.9) | 85-89  4.17 (3.55, 4.66) | 55-59  90.17 (80.03, 100.91) |
|  | Female | 70-74  2.07 (1.78, 2.4) | 95+  3.05 (2.17, 3.65) | 55-59  46.82 (41.11, 53.65) |
|  | Male | 65-69  6.61 (5.53, 7.97) | 85-89  6.75 (5.88, 7.64) | 55-59  134.92 (117.18, 155.93) |
| **Central Asia** | Both | 95+  2.06 (1.56, 2.49) | 95+  3.3 (2.52, 4.04) | 65-69  38.59 (32.53, 45.91) |
|  | Female | 95+  1.45 (1.07, 1.79) | 95+  2.32 (1.73, 2.89) | 65-69  21.64 (18.06, 26.11) |
|  | Male | 95+  5.62 (4.37, 6.87) | 95+  9.03 (7.06, 11.03) | 95+  75.34 (59.05, 92.11) |
| **Central Europe** | Both | 95+  2.07 (1.56, 2.44) | 95+  3.3 (2.52, 3.9) | 55-59  47.21 (41.33, 54.03) |
|  | Female | 95+  2.27 (1.7, 2.68) | 95+  3.63 (2.75, 4.27) | 95+  30.23 (22.84, 35.51) |
|  | Male | 55-59  2.58 (2.26, 2.95) | 90-94  3.12 (2.56, 3.57) | 55-59  78.58 (68.74, 89.41) |
| **Eastern Europe** | Both | 55-59  1.2 (1.03, 1.39) | 65-69  1.19 (1.04, 1.38) | 55-59  38.96 (33.59, 45.45) |
|  | Female | 70-74  0.54 (0.48, 0.63) | 95+  0.8 (0.6, 0.94) | 55-59  14.33 (12.29, 16.99) |
|  | Male | 65-69  2.18 (1.84, 2.61) | 65-69  2.28 (1.93, 2.73) | 55-59  69.29 (58.27, 82.45) |
| **Australasia** | Both | 65-69  2.06 (1.51, 2.7) | 90-94  2.29 (1.56, 3.18) | 55-59  25.35 (19.72, 31.59) |
|  | Female | 95+  1.75 (1.06, 2.49) | 95+  2.12 (1.33, 3.06) | 95+  17.55 (10.99, 25.23) |
|  | Male | 65-69  3.45 (2.52, 4.55) | 90-94  3.63 (2.44, 5.05) | 65-69  40.5 (29.04, 52.94) |
| **High-income Asia Pacific** | Both | 90-94  2.86 (2.22, 3.25) | 90-94  4.16 (3.24, 4.72) | 90-94  36.71 (28.4, 41.53) |
|  | Female | 95+  1.68 (1.07, 2.06) | 95+  2.67 (1.7, 3.25) | 95+  21.2 (13.56, 25.87) |
|  | Male | 90-94  6.54 (5.41, 7.21) | 90-94  9.51 (7.9, 10.45) | 90-94  83.85 (69.61, 92.26) |
| **High-income North America** | Both | 65-69  1.57 (1.46, 1.67) | 95+  1.27 (0.9, 1.51) | 55-59  24.84 (23.7, 26.05) |
|  | Female | 55-59  0.79 (0.74, 0.85) | 95+  1.03 (0.71, 1.23) | 55-59  12.39 (11.7, 13.17) |
|  | Male | 65-69  2.51 (2.34, 2.69) | 95+  1.94 (1.39, 2.29) | 55-59  37.87 (35.97, 39.74) |
| **Southern Latin America** | Both | 95+  1.61 (1.11, 2.25) | 95+  2.56 (1.77, 3.56) | 95+  21.19 (14.67, 29.46) |
|  | Female | 95+  1.57 (1.04, 2.22) | 95+  2.5 (1.66, 3.5) | 95+  20.83 (13.82, 29.16) |
|  | Male | 90-94  1.68 (1.28, 2.19) | 95+  2.66 (1.85, 3.67) | 55-59  26.46 (20.73, 33.31) |
| **Western Europe** | Both | 95+  1.8 (1.35, 2.14) | 95+  2.64 (1.98, 3.13) | 55-59  26.46 (23.64, 30.14) |
|  | Female | 95+  1.48 (1.05, 1.79) | 95+  2.21 (1.56, 2.66) | 95+  18.26 (12.94, 21.99) |
|  | Male | 95+  2.8 (2.17, 3.47) | 95+  3.99 (3.09, 4.87) | 55-59  42.1 (37.65, 47.87) |
| **Andean Latin America** | Both | 90-94  1.67 (1.26, 2.18) | 90-94  2.45 (1.86, 3.19) | 90-94  21.62 (16.37, 28.15) |
|  | Female | 90-94  1.51 (1.07, 2.07) | 90-94  2.2 (1.58, 3.03) | 90-94  19.47 (13.96, 26.59) |
|  | Male | 90-94  1.91 (1.34, 2.69) | 90-94  2.8 (1.97, 3.94) | 90-94  24.7 (17.32, 34.62) |
| **Caribbean** | Both | 85-89  4.07 (3.39, 4.81) | 85-89  5.47 (4.57, 6.44) | 85-89  55.46 (46.47, 65.34) |
|  | Female | 90-94  2.63 (2.03, 3.27) | 90-94  3.83 (2.99, 4.79) | 85-89  35.44 (27.86, 43.9) |
|  | Male | 85-89  6.12 (4.97, 7.48) | 85-89  8.19 (6.65, 10.03) | 55-59  84.79 (67.22, 106.19) |
| **Central Latin America** | Both | 90-94  1.24 (1.01, 1.42) | 90-94  1.81 (1.49, 2.07) | 70-74  20.1 (17.37, 23.62) |
|  | Female | 90-94  0.74 (0.57, 0.89) | 95+  1.14 (0.78, 1.45) | 70-74  9.94 (8.49, 11.72) |
|  | Male | 85-89  1.99 (1.69, 2.28) | 90-94  2.89 (2.4, 3.33) | 70-74  32.03 (27.49, 37.46) |
| **Tropical Latin America** | Both | 55-59  0.86 (0.77, 0.95) | 90-94  1.13 (0.86, 1.35) | 55-59  27.16 (24.37, 30.15) |
|  | Female | 90-94  0.7 (0.51, 0.85) | 90-94  1.03 (0.75, 1.25) | 55-59  11.51 (10.1, 13.08) |
|  | Male | 55-59  1.41 (1.26, 1.57) | 90-94  1.34 (1.05, 1.57) | 55-59  44.7 (39.98, 49.65) |
| **North Africa and Middle East** | Both | 95+  3.59 (2.53, 4.77) | 95+  5.7 (4, 7.61) | 65-69  53.55 (43.84, 64.1) |
|  | Female | 95+  3.26 (2.1, 4.76) | 95+  5.19 (3.32, 7.69) | 95+  43.32 (27.79, 63.83) |
|  | Male | 90-94  4.17 (3.16, 5.34) | 95+  6.26 (4.19, 9.25) | 65-69  78.84 (61.65, 97.52) |
| **South Asia** | Both | 70-74  3.94 (3.38, 4.61) | 70-74  4.33 (3.73, 5.08) | 55-59  108.59 (95, 123.44) |
|  | Female | 70-74  2.24 (1.86, 2.71) | 85-89  2.7 (2.21, 3.2) | 55-59  67.93 (56.09, 81.01) |
|  | Male | 70-74  5.71 (4.69, 6.98) | 90-94  6.3 (5.26, 7.49) | 55-59  149.19 (124.96, 175.24) |
| **East Asia** | Both | 65-69  8.39 (6.55, 10.65) | 95+  10.11 (7.36, 12.56) | 60-64  149.63 (118.48, 190.86) |
|  | Female | 95+  5.07 (3.41, 6.73) | 95+  7.8 (5.27, 10.36) | 70-74  76.07 (58.81, 96.49) |
|  | Male | 85-89  13.61 (10.81, 16.58) | 95+  18.71 (13.79, 24.63) | 60-64  228.72 (170.61, 305.53) |
| **Oceania** | Both | 65-69  4.04 (2.7, 6.34) | 65-69  4.22 (2.86, 6.5) | 65-69  104.46 (70.81, 160.99) |
|  | Female | 75-79  2.33 (1.56, 3.65) | 75-79  2.74 (1.85, 4.29) | 50-54  54.96 (32.35, 116.47) |
|  | Male | 65-69  6.05 (3.72, 10.01) | 65-69  6.32 (3.91, 10.21) | 65-69  156.49 (97.09, 252.39) |
| **Southeast Asia** | Both | 70-74  6.75 (5.83, 7.85) | 85-89  8.97 (7.41, 10.46) | 65-69  158.08 (136.53, 184.07) |
|  | Female | 85-89  5.49 (4.3, 6.72) | 85-89  7.43 (5.86, 8.98) | 70-74  86.22 (71.85, 101.25) |
|  | Male | 70-74  10.23 (8.4, 12.19) | 80-84  11.85 (10, 14.05) | 55-59  243.78 (199.6, 300.86) |
| **Central Sub-Saharan Africa** | Both | 70-74  1.61 (1.12, 2.34) | 90-94  2.14 (1.21, 4.04) | 55-59  43.42 (29.61, 65.16) |
|  | Female | 95+  1.26 (0.55, 3.27) | 95+  2.03 (0.9, 5.46) | 65-69  28.78 (17.93, 45.79) |
|  | Male | 85-89  2.23 (1.16, 3.83) | 90-94  3.11 (1.47, 5.88) | 55-59  61.18 (37.6, 97.2) |
| **Eastern Sub-Saharan Africa** | Both | 70-74  5.3 (4.08, 6.75) | 70-74  5.86 (4.5, 7.5) | 55-59  150.59 (109.22, 200.36) |
|  | Female | 70-74  3.53 (2.51, 5) | 85-89  4.67 (3.25, 6.63) | 55-59  101.21 (71.4, 154.7) |
|  | Male | 70-74  7.3 (5.25, 10.04) | 70-74  8.08 (5.82, 11.12) | 55-59  202.1 (138, 293.86) |
| **Southern Sub-Saharan Africa** | Both | 85-89  2.43 (2.15, 2.73) | 85-89  3.29 (2.92, 3.69) | 60-64  60.49 (53.78, 68.73) |
|  | Female | 80-84  1.97 (1.64, 2.31) | 95+  2.88 (2.18, 3.55) | 70-74  37.32 (31.82, 44.41) |
|  | Male | 65-69  3.67 (3.14, 4.2) | 90-94  4.95 (4.18, 5.77) | 60-64  94.54 (81.19, 110.77) |
| **Western Sub-Saharan Africa** | Both | 55-59  2.37 (1.62, 3.15) | 85-89  2.71 (2.32, 3.16) | 55-59  78.58 (53.73, 105.2) |
|  | Female | 70-74  2.04 (1.45, 2.82) | 85-89  2.76 (2.21, 3.53) | 70-74  46.08 (33, 64.46) |
|  | Male | 55-59  3.55 (2.3, 4.94) | 55-59  3.46 (2.26, 4.87) | 55-59  117.7 (76.73, 165.35) |
| ** Values represent the rate in the highest age group in 2021 (highest age group; val [95% UI] per 100,000).* ***Abbreviations:*** *DALYs, Disability-adjusted life years.* | | | | |
