# Supplementary figures and images for: Shifting burden of nasopharyngeal carcinoma: global patterns and forecasts to 2050 from the GBD 2021
Source: Front Oncol. 2026 Jan 6;15:1687320. doi: 10.3389/fonc.2025.1687320 (PMC12815733; doi:10.3389/fonc.2025.1687320)

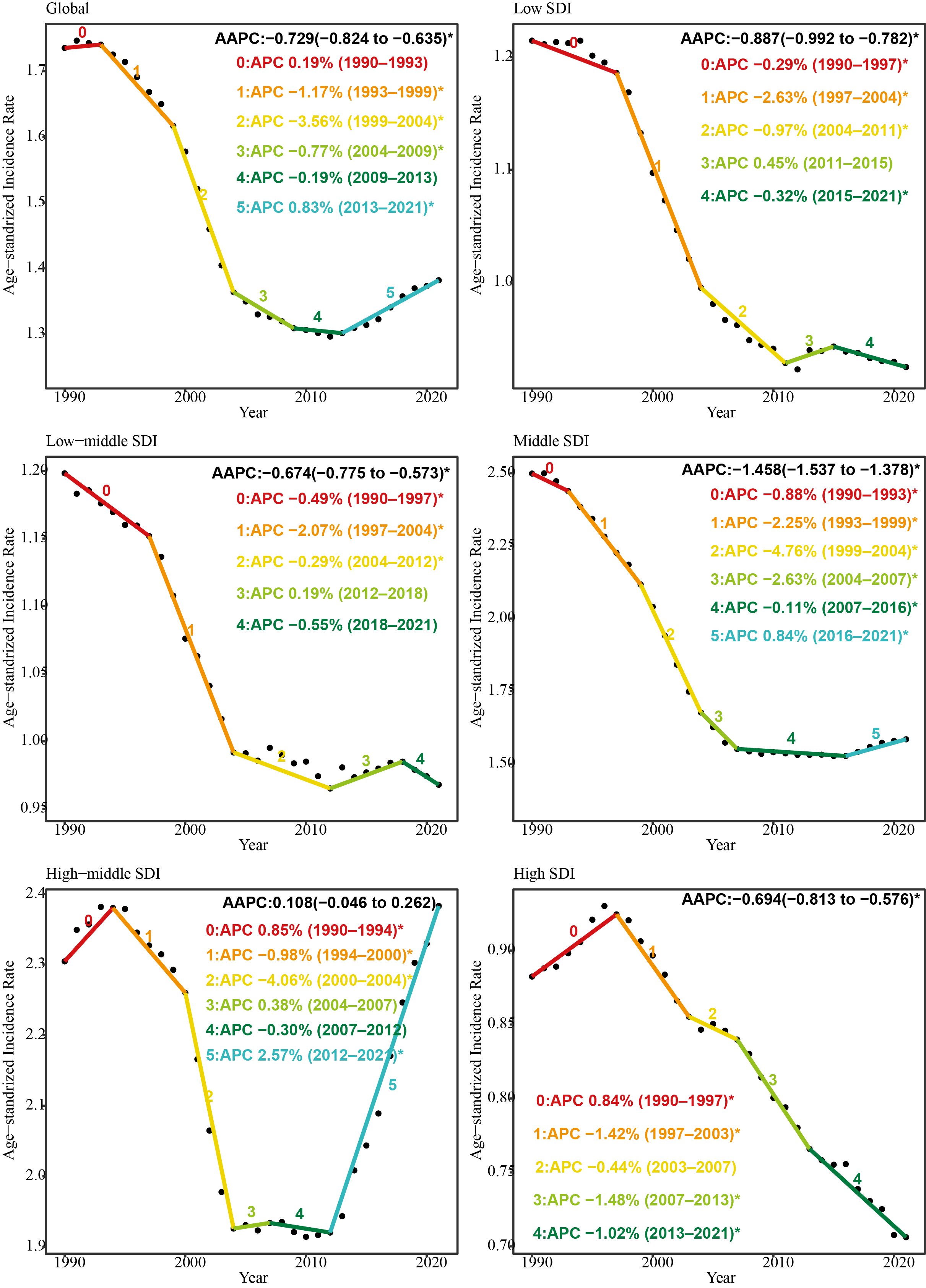

Supplement: Supplementary Figure 1 — Annual percent change (APC) and average annual percent change (AAPC) in age-standardized incidence rate (ASIR) at the global level and across five SDI regions, both sexes combined, 1990–2021. ASIR, age-standardized incidence rate; APC, annual percent change; AAPC, average annual percent change. *, P value <0.01. [file Image1.png]

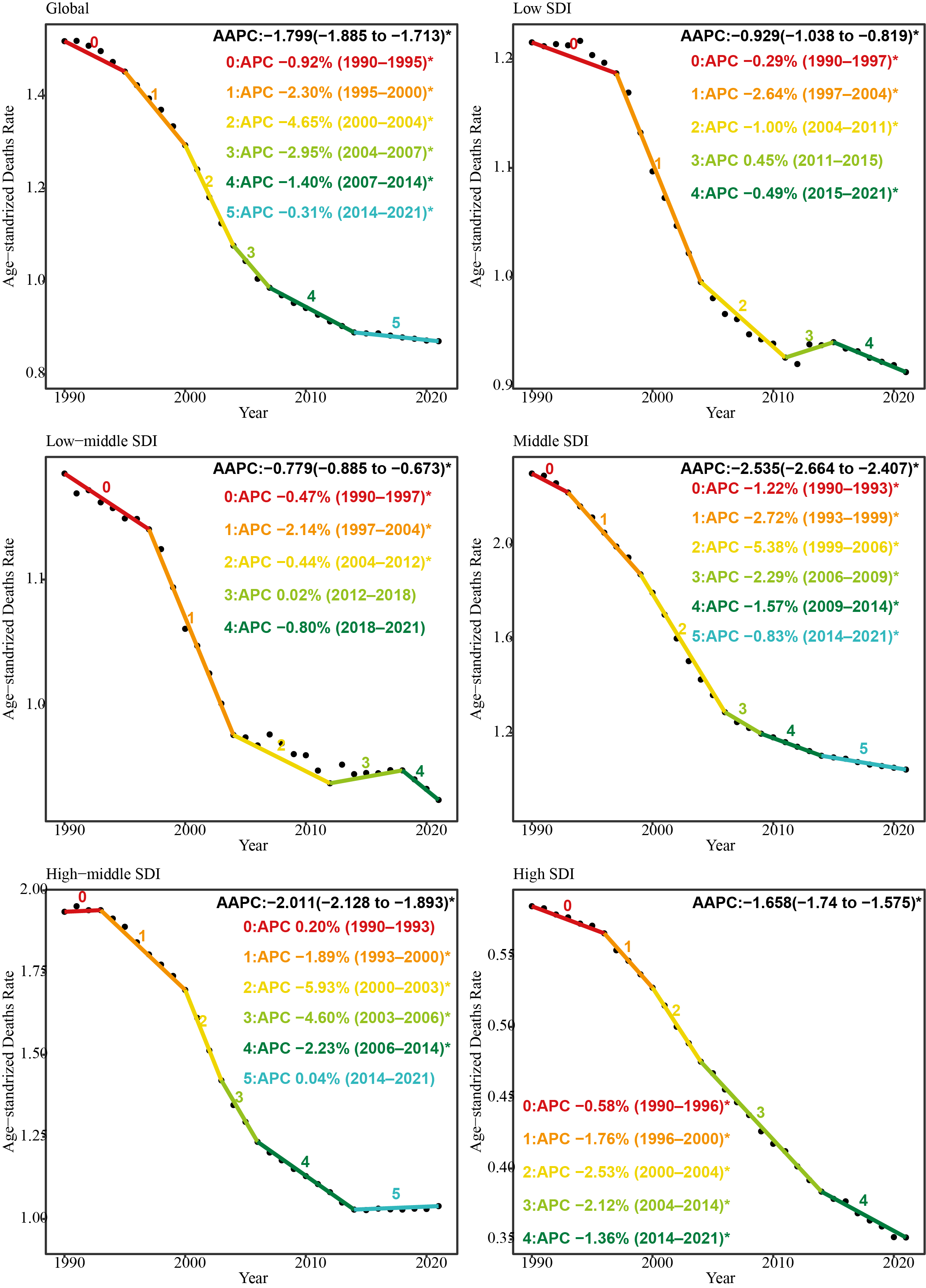

Supplement: Supplementary Figure 2 — Annual percent change (APC) and average annual percent change (AAPC) in age-standardized death rate (ASDR) for the global level and five SDI regions, both sexes combined, 1990–2021. ASDR, age-standardized death rate; APC, annual percent change; AAPC, average annual percent change. *, P value <0.01. [file Image2.png]

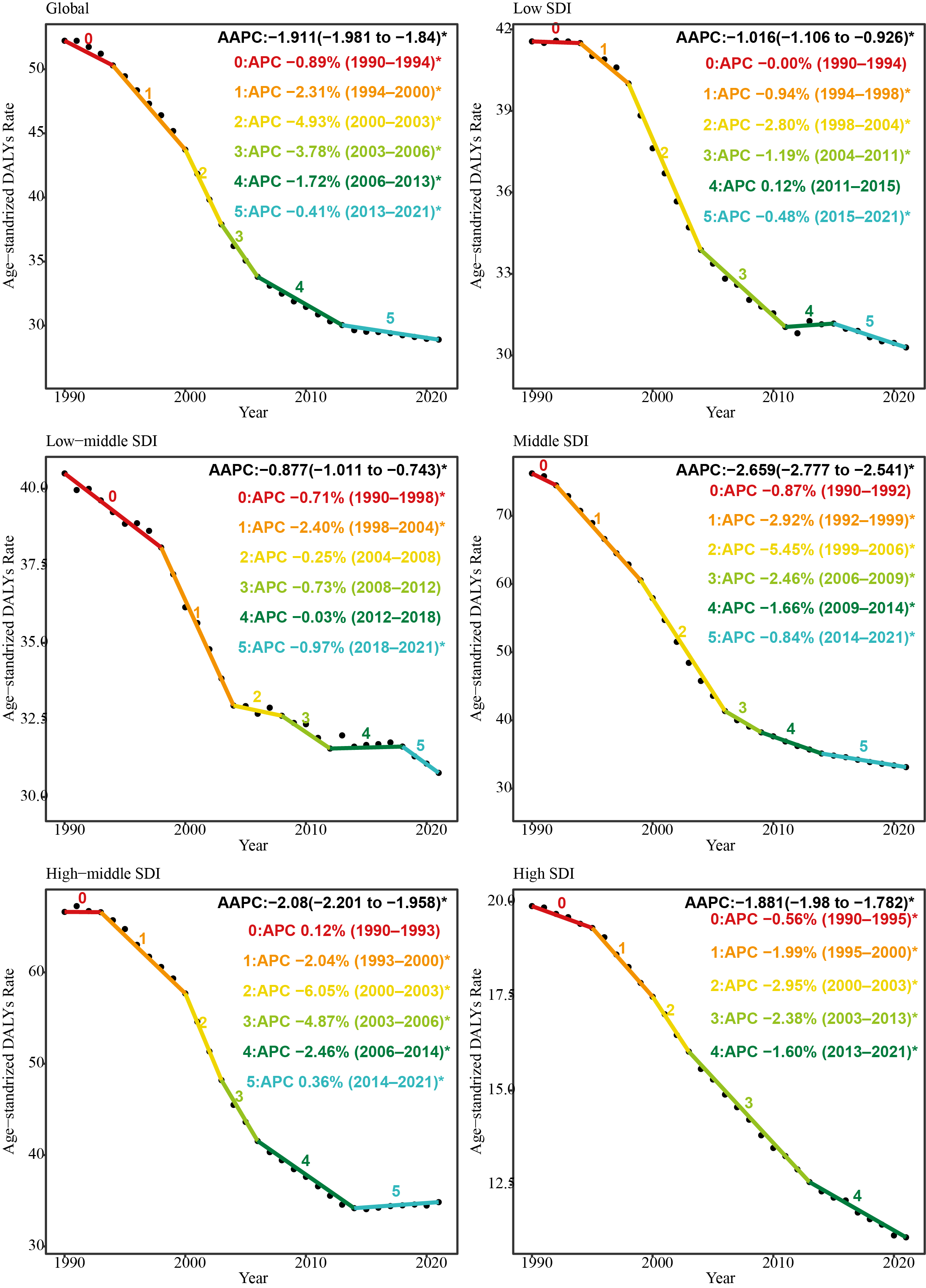

Supplement: Supplementary Figure 3 — Annual percent change (APC) and average annual percent change (AAPC) in age-standardized DALYs rate for the global level and five SDI regions, both sexes combined, 1990–2021. DALYs, Disability-adjusted life year; APC, annual percent change; AAPC, average annual percent change. *, P value <0.01. [file Image3.png]

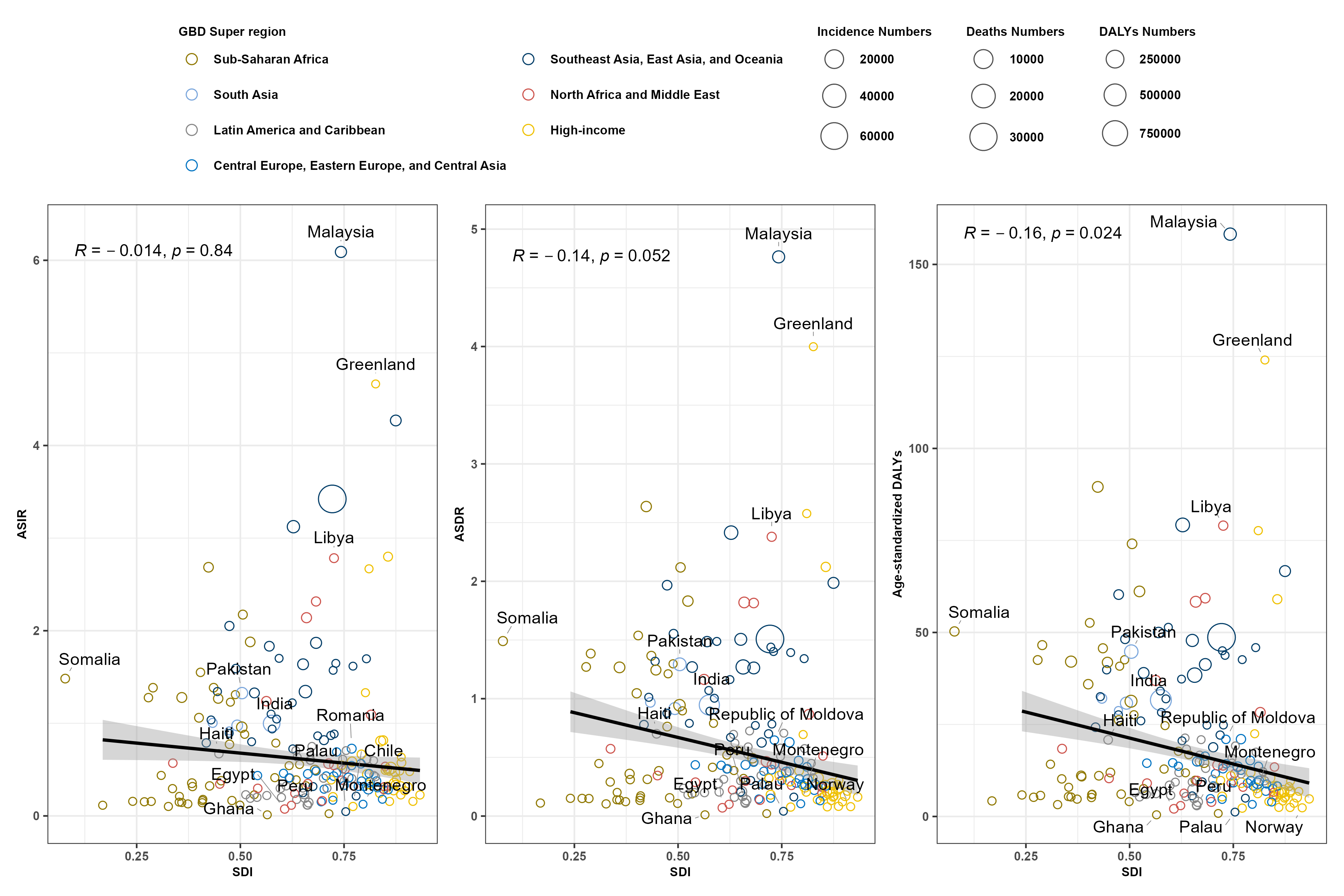

Supplement: Supplementary Figure 4 — Disease burden of nasopharyngeal carcinoma by socio-demographic index (SDI) across 204 countries or territories in 2021. ASIR, age-standardized incidence rate; ASDR, age-standardized death rate; DALYs, disability-adjusted life years; SDI, Socio-demographic Index. [file Image4.png]

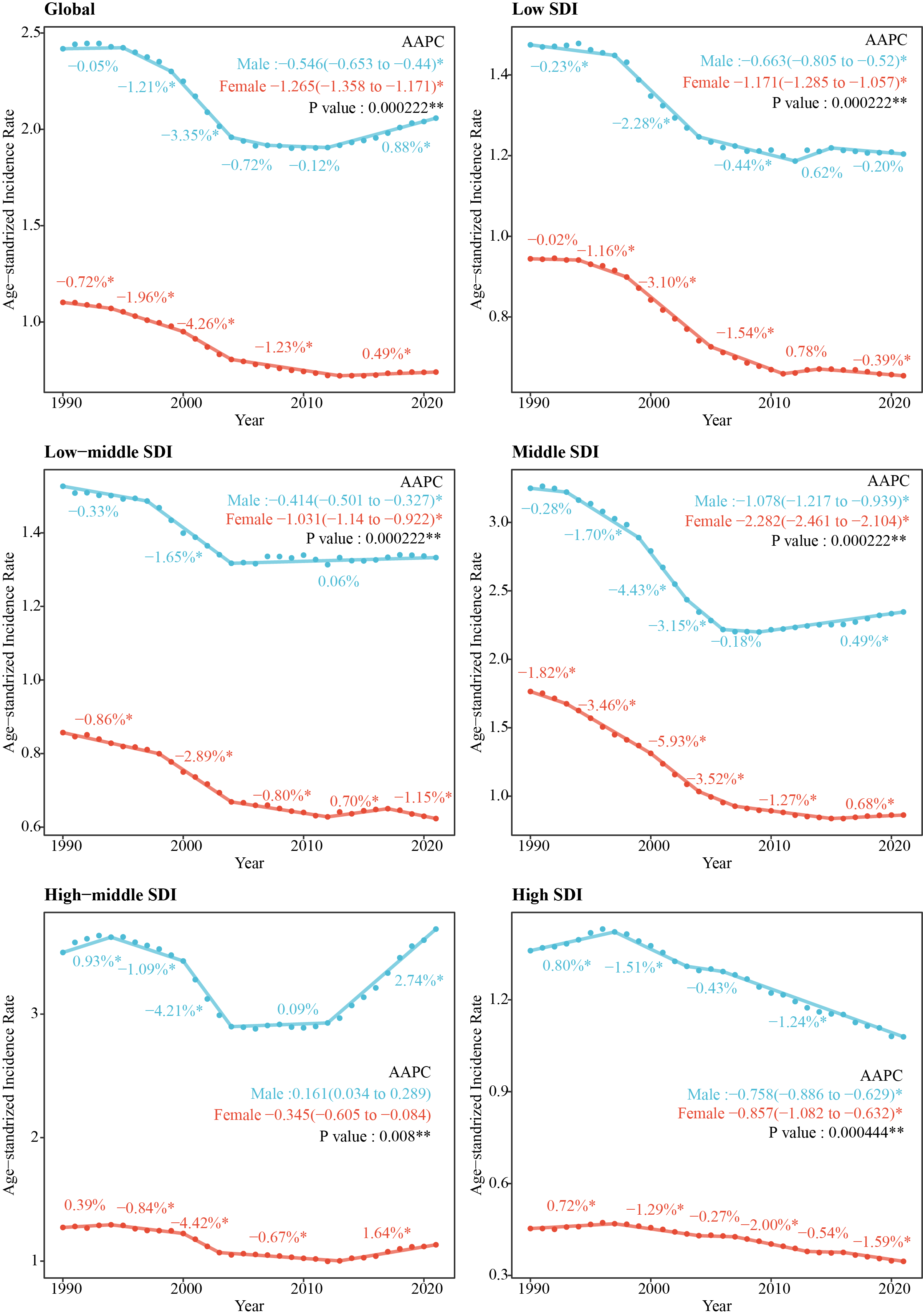

Supplement: Supplementary Figure 5 — Annual percent change (APC) and average annual percent change (AAPC) in age-standardized incidence rate across five SDI regions, by sex, 1990–2021. Joinpoint regression was conducted using a sex-stratified group comparison approach, with male and female trends modelled concurrently. * P value of APC <0.01; ** P value of AAPC between female and males <0.01. APC, annual percent change; AAPC, average annual percent change [file Image5.png]

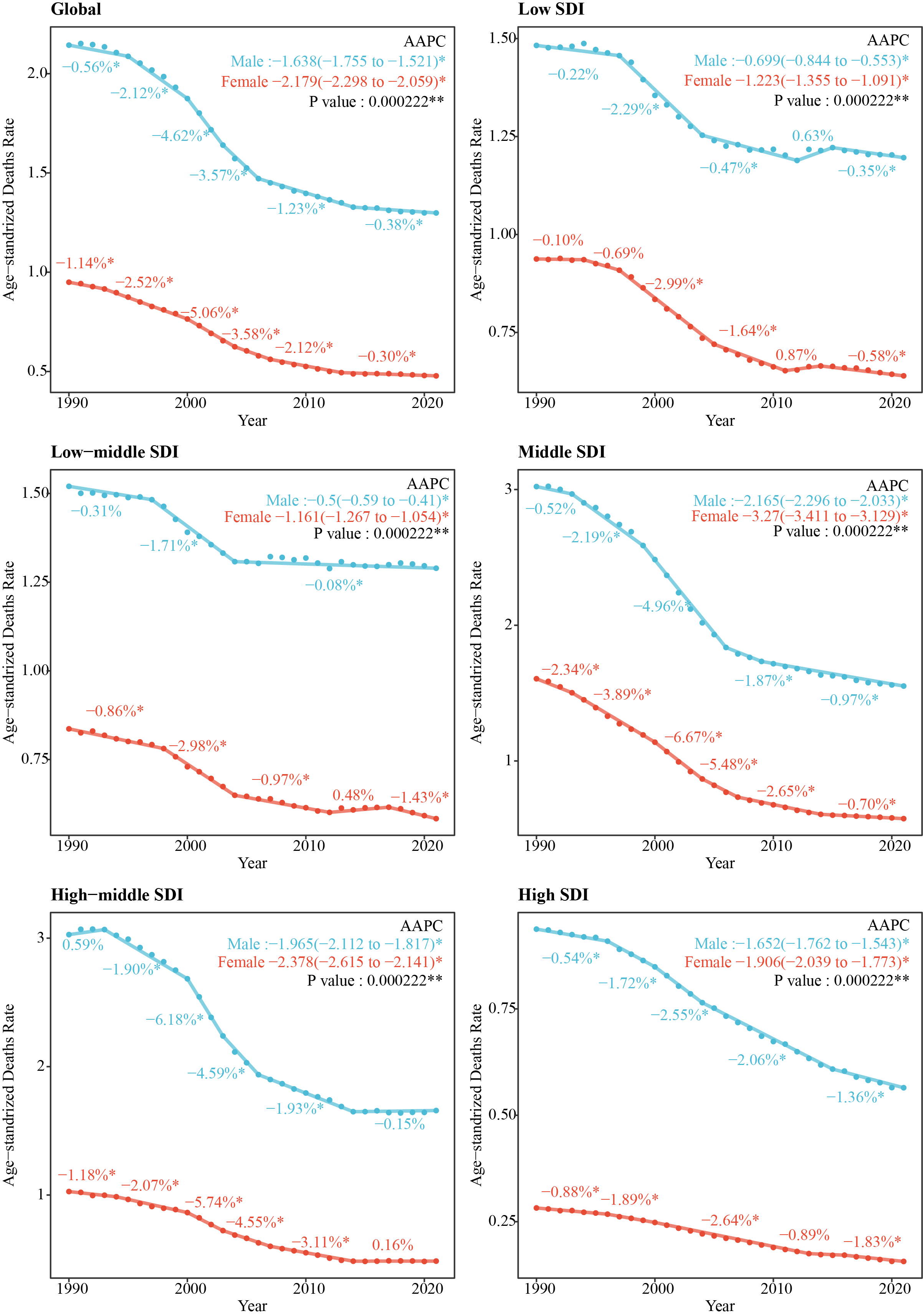

Supplement: Supplementary Figure 6 — Annual percent change (APC) and average annual percent change (AAPC) in age-standardized death rate across five SDI regions, by sex, 1990–2021. Joinpoint regression was conducted using a sex-stratified group comparison approach, with male and female trends modelled concurrently. * P value of APC <0.01; ** P value of AAPC between female and males <0.01. APC, annual percent change; AAPC, average annual percent change [file Image6.png]

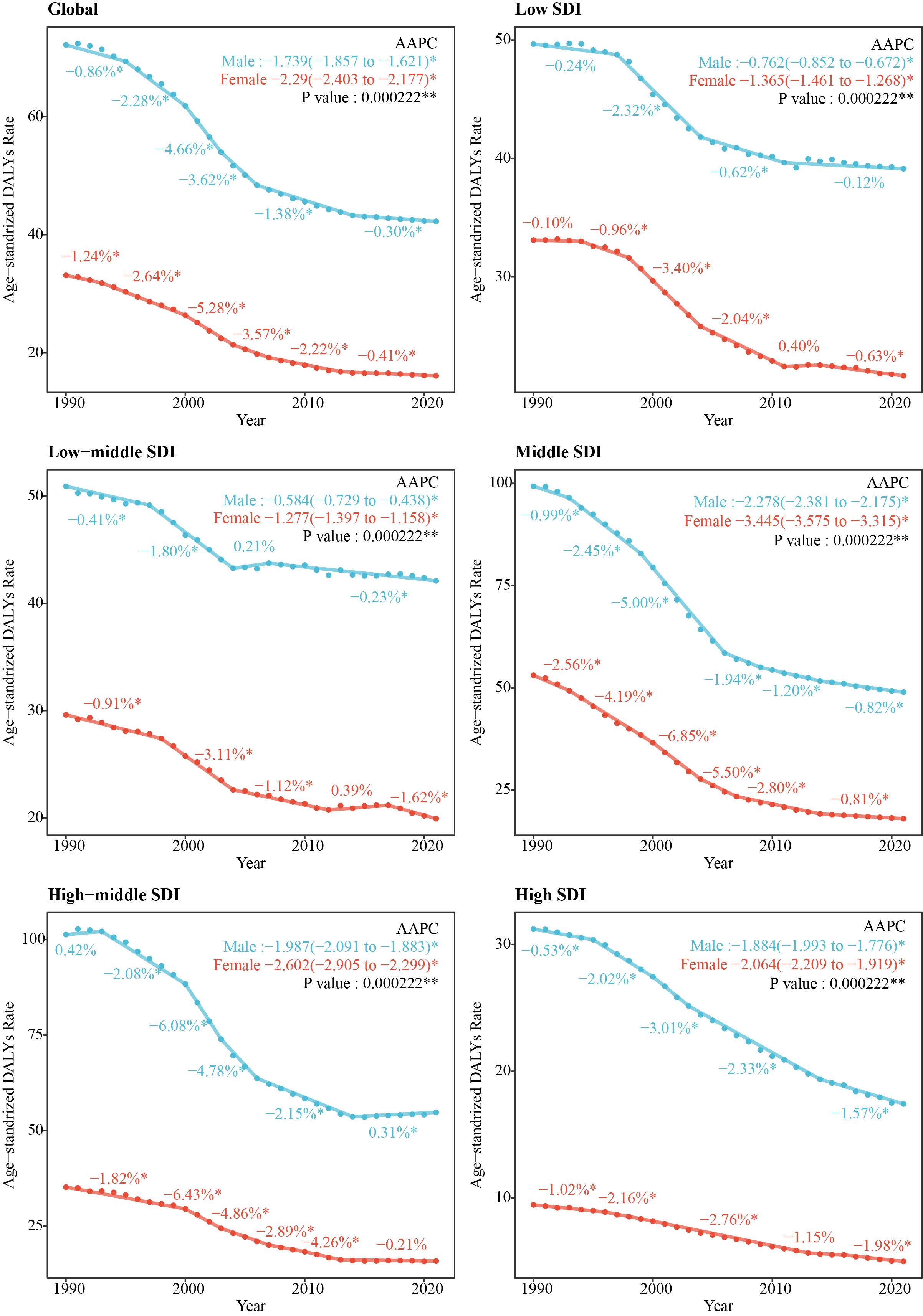

Supplement: Supplementary Figure 7 — Annual percent change (APC) and average annual percent change (AAPC) in age-standardized DALYs rate across five SDI regions, by sex, 1990–2021. Joinpoint regression was conducted using a sex-stratified group comparison approach, with male and female trends modelled concurrently. * P value of APC <0.01; ** P value of AAPC between female and males <0.01. DALYs, Disability-adjusted life year; APC, annual percent change; AAPC, average annual percent change. [file Image7.png]

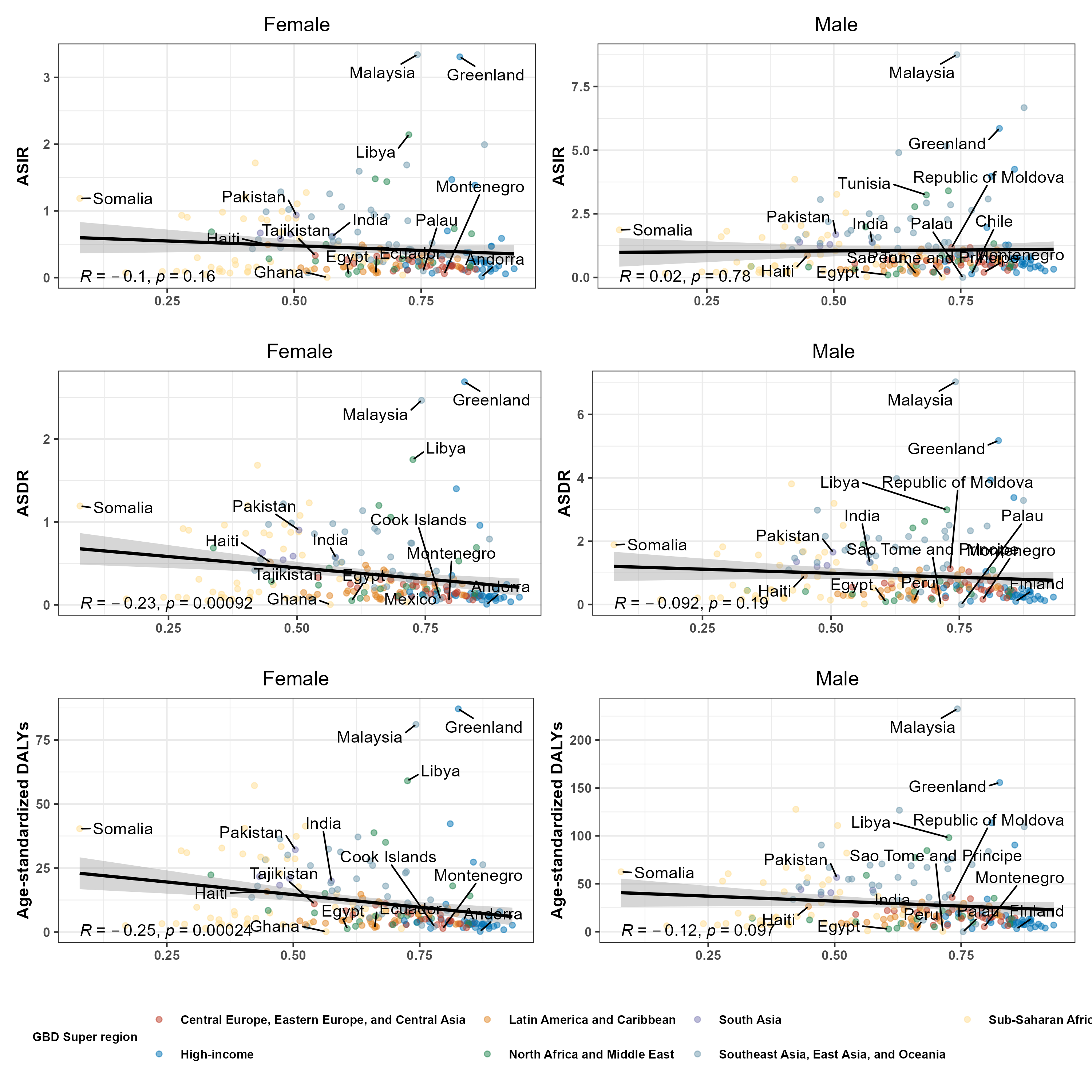

Supplement: Supplementary Figure 8 — Disease burden of nasopharyngeal carcinoma by different gender across 204 countries or territories and socio-demographic index level in 2021. ASIR, age-standardized incidence rate; ASDR, age-standardized death rate; DALYs, disability-adjusted life years; SDI, Socio-demographic Index. [file Image8.png]

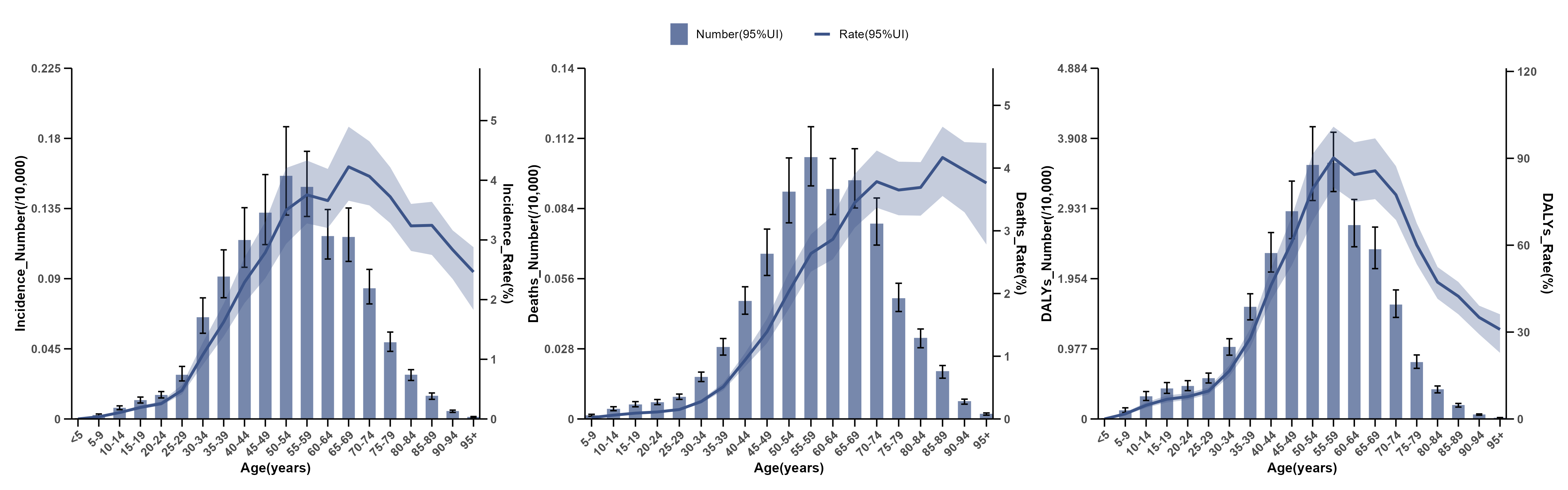

Supplement: Supplementary Figure 9 — Number and rate for incidence, death, and disability-adjusted life years (DALYs) of nasopharyngeal carcinoma across different age groups in 2021. DALYs, disability-adjusted life years [file Image9.png]

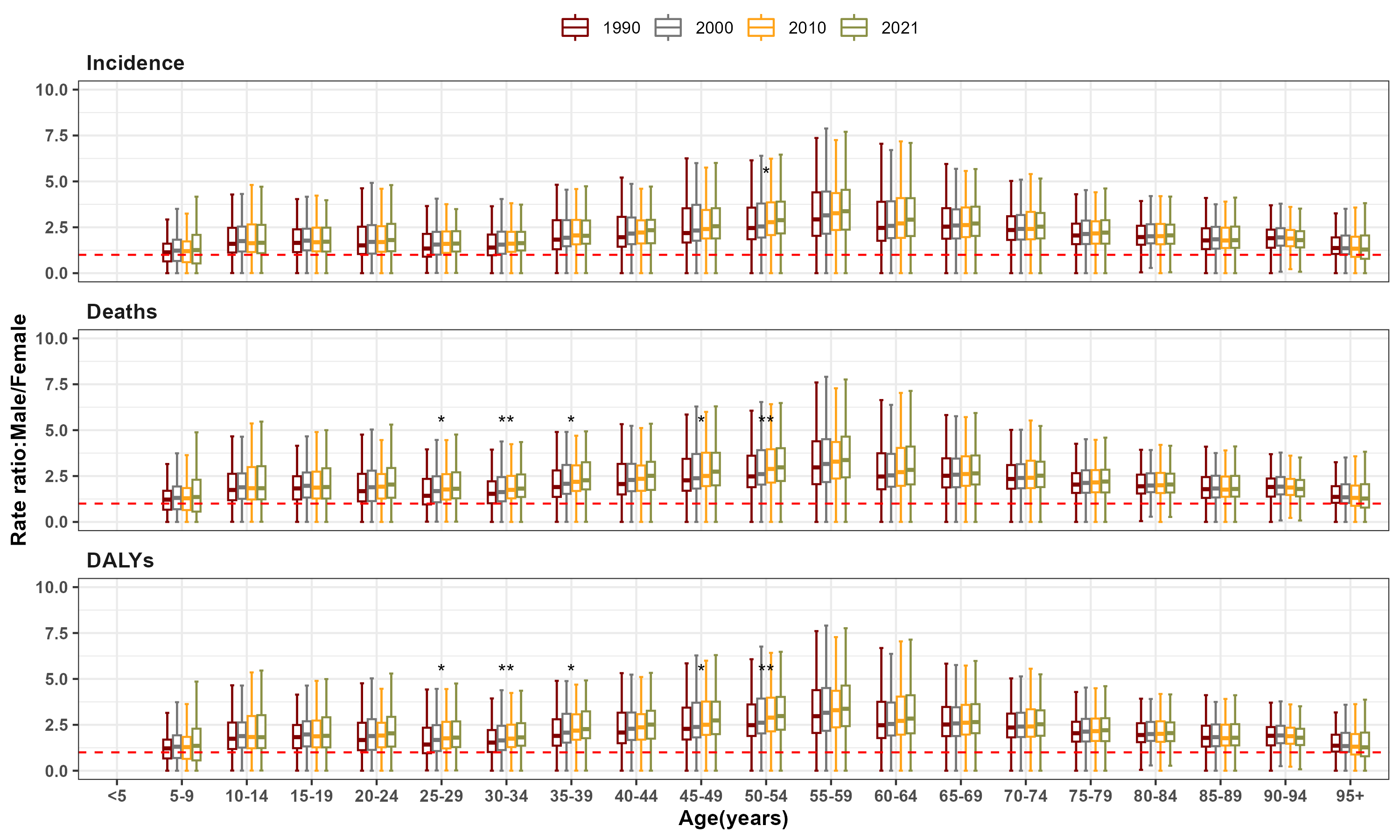

Supplement: Supplementary Figure 10 — Rate ratio for male to female in incidence, death, and disability-adjusted life years (DALYs) of nasopharyngeal carcinoma across different age groups in 1990, 2000, 2010, and 2021. DALYs, disability-adjusted life years. [file Image10.png]

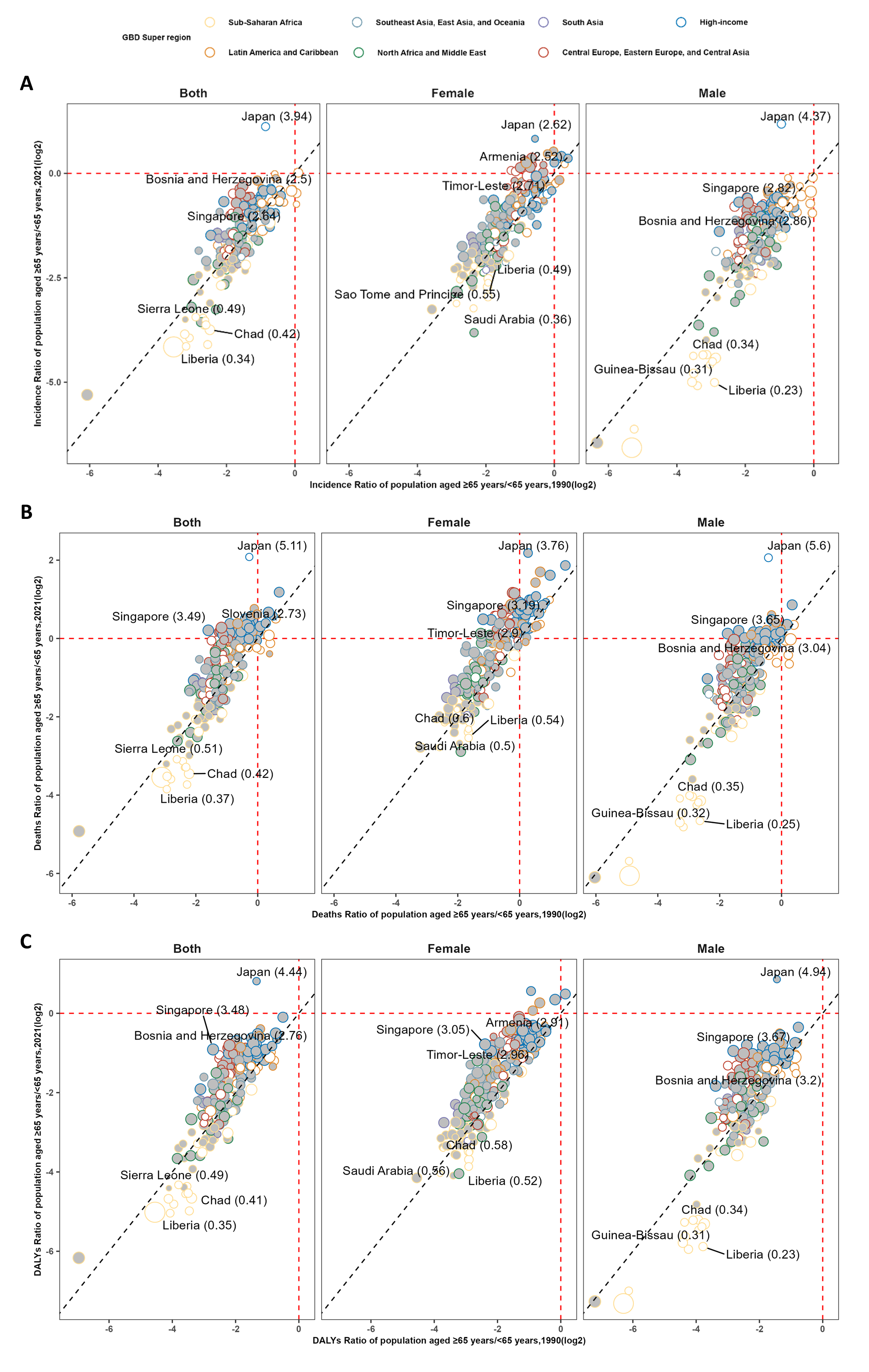

Supplement: Supplementary Figure 11 — Aging-related ratio for incidence, death, and disability-adjusted life years (DALYs) of nasopharyngeal carcinoma by gender in 204 countries or territories. DALYs, disability-adjusted life years. [file Image11.png]

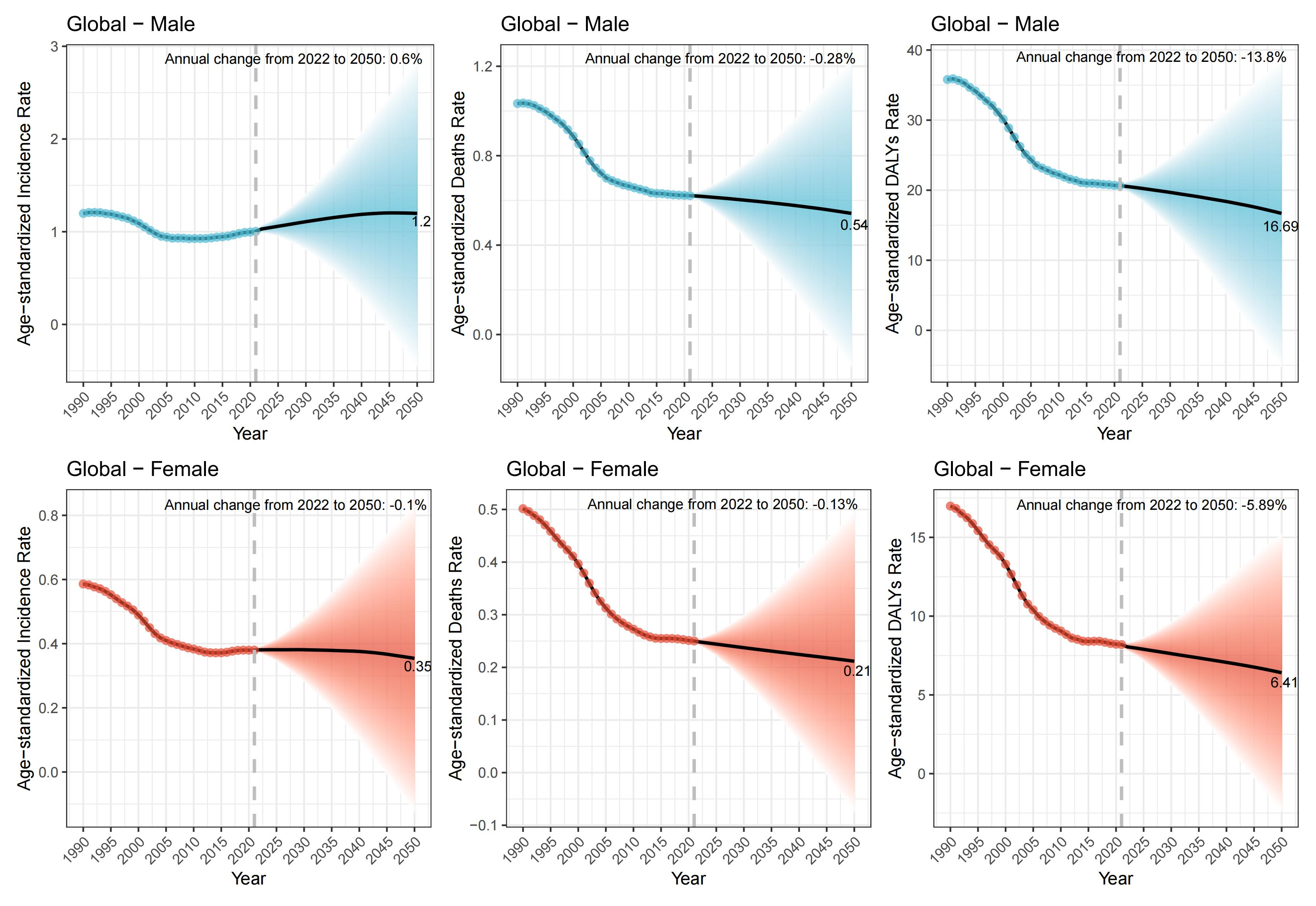

Supplement: Supplementary Figure 12 — The temporal and predicted trends for disease burden of nasopharyngeal carcinoma by different gender from 1990 to 2050. Blue spots represent the male levels from 1990 to 2021. Red spots represent the female levels from 1990 to 2021. The dotted line represents the beginning of the prediction (from 2022). Shading indicates the upper and lower limits of the 95% UI. DALYs, disability-adjusted life years [file Image12.png]
